# Supplementary material for: Uranium-stibinidiide, -stibinidene, and -stibido multiple bonds and uranium-nitride formation from multimetallic diuranium-distibene-mediated dinitrogen cleavage
Source: Nat Commun. 2025 Aug 4;16:7136. doi: 10.1038/s41467-025-61612-5 (PMC12322283; doi:10.1038/s41467-025-61612-5)
Supplement: Supplementary file 1 — Supplementary Information [file 41467_2025_61612_MOESM1_ESM.pdf]

– Supplementary Information –

Uranium-stibinidiide, -stibinidene, and -stibido multiple bonds and uranium-nitride formation from multimetallic diuranium-distibene-mediated dinitrogen cleavage

Rebecca F. Sheppard,[1] Kevin Dollberg,[2] Nick Michel,[2] John A. Seed,[1] Ashley J. Wooles,[1] Jingzhen Du,[1,3*] Carsten von Hänisch,[2,*] Stephen T. Liddle[1,*]

[1] Department of Chemistry and Centre for Radiochemistry Research, The University of Manchester, Manchester, UK.

[2] Fachbereich Chemie and Marburg Center for Quantum Materials and Sustainable Technologies (mar.quest), Philipps-Universität Marburg, Marburg, Germany.

[3] Present address: College of Chemistry, Zhengzhou University, Zhengzhou, China.

*Email: jingzhendu@zzu.edu.cn; haenisch@chemie.uni-marburg.de; steve.liddle@manchester.ac.uk

Figures

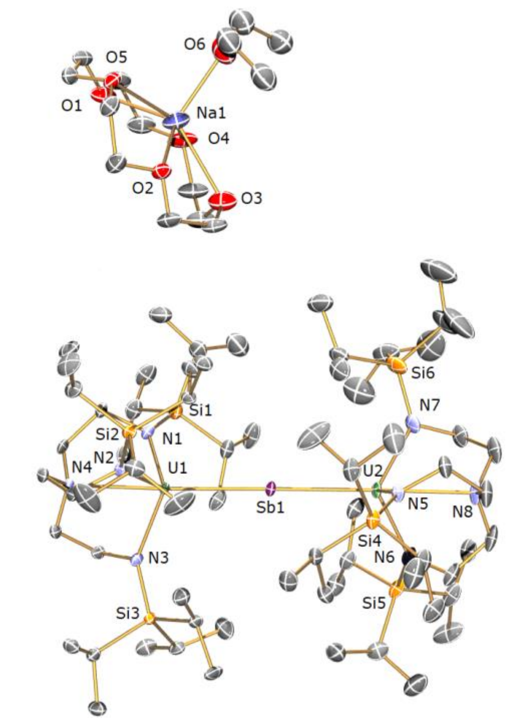

Supplementary Figure 1. Solid-state structure of complex **5UNa** at 100 K. Thermal ellipsoids are set at 40% probability and hydrogen atoms, disordered components and lattice solvent are omitted for clarity. CCDC number 2404124.

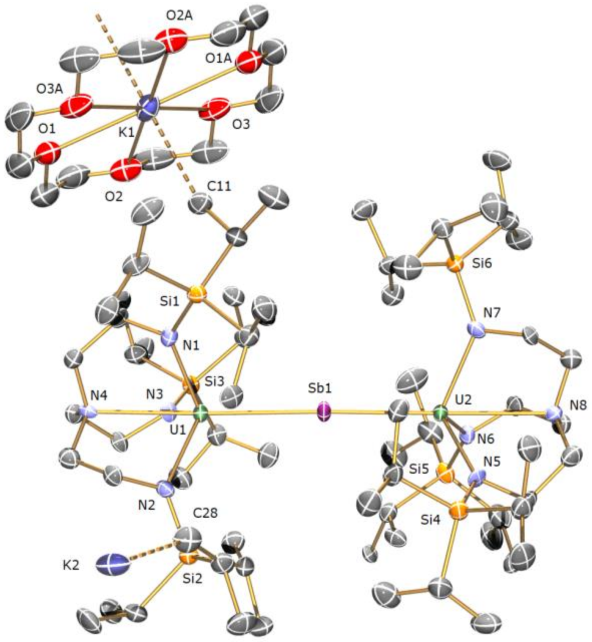

Supplementary Figure 2. Solid-state structure of complex **5UK** at 150 K. Thermal ellipsoids are set at 40% probability. Hydrogen atoms, lattice solvent and the bridging crown unit bound to K2 are omitted for clarity. CCDC number 2404125.

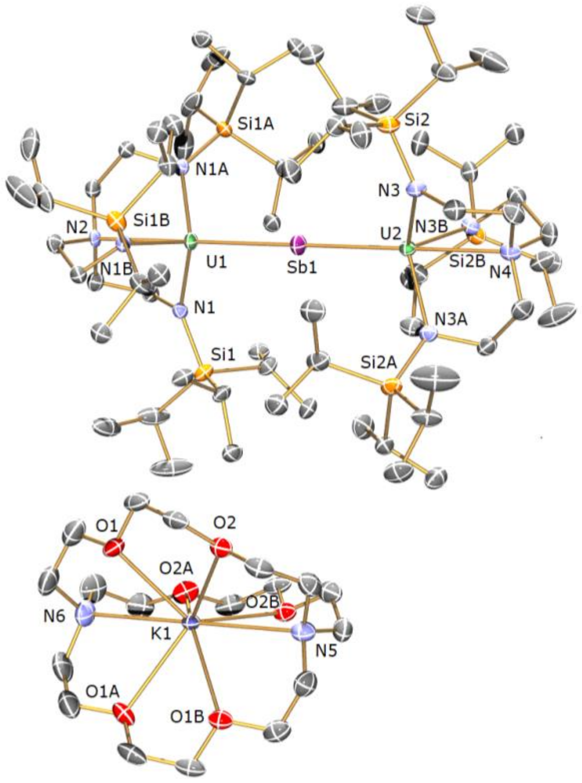

Supplementary Figure 3. Solid-state structure of complex **5UK′** at 100 K. Thermal ellipsoids are set at 40% probability and hydrogen atoms, disordered components and lattice solvent are omitted for clarity. CCDC number 2404126.

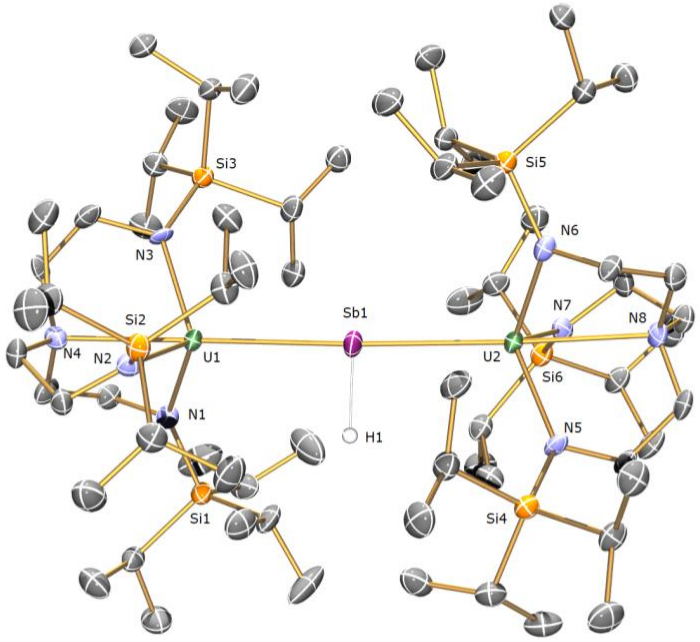

Supplementary Figure 4. Solid-state structure of complex **6U** at 150 K. Thermal ellipsoids are set at 40% probability and hydrogen atoms, except H1, and disordered components are omitted for clarity. CCDC number 2404127.

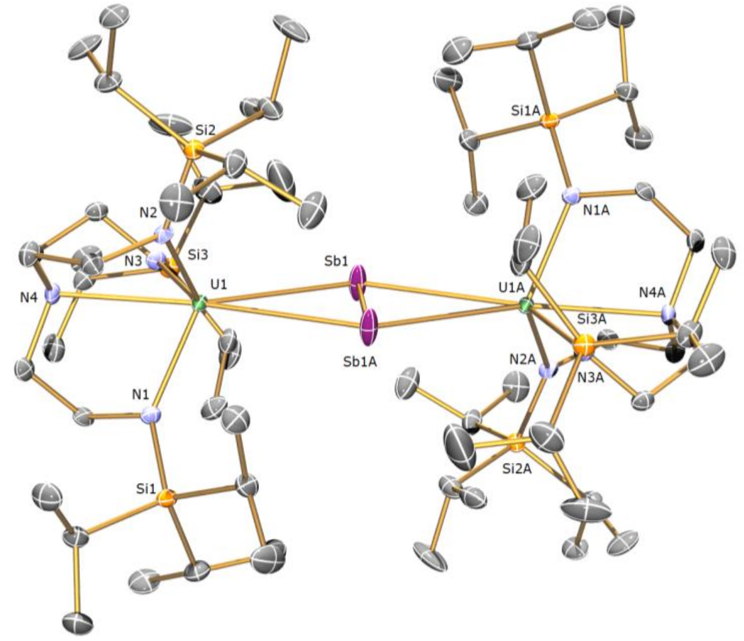

Supplementary Figure 5. Solid-state structure of complex **7U** at 150 K. Thermal ellipsoids are set at 40% probability and hydrogen atoms and the minor disordered components of **6U** are omitted for clarity. CCDC number 2404128.

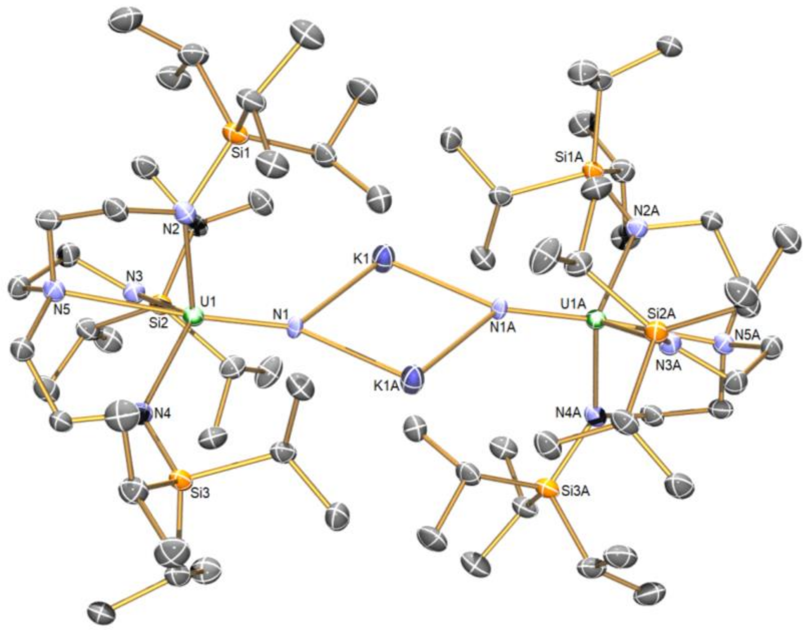

Supplementary Figure 6. Solid-state structure of complex **8U** at 100 K. Thermal ellipsoids are set at 40% probability and hydrogen atom and K···H_3C interactions are omitted for clarity. CCDC number 2434801.

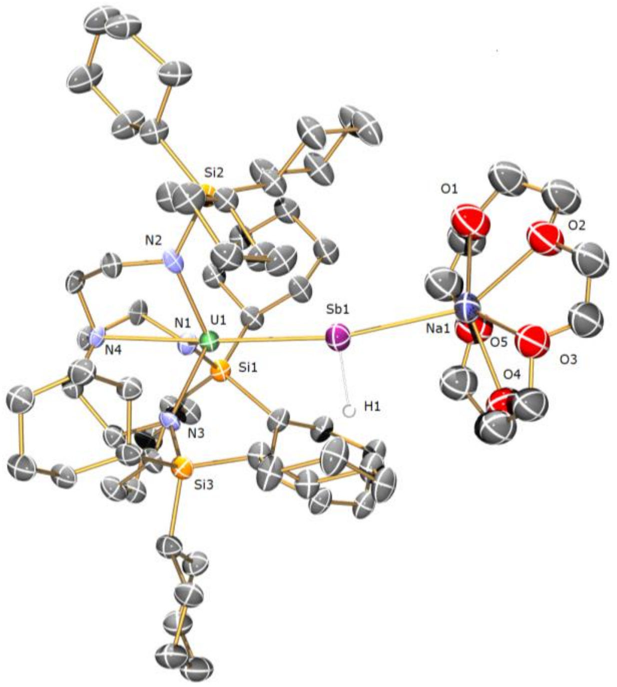

Supplementary Figure 7. Solid-state structure of complex **9UNa** at 100 K. Thermal ellipsoids are set at 40% probability and hydrogen atoms except H1 and disordered components are omitted for clarity. The second unit of **9UNa** in the asymmetric unit is very similar and not shown for clarity. CCDC number 2404129.

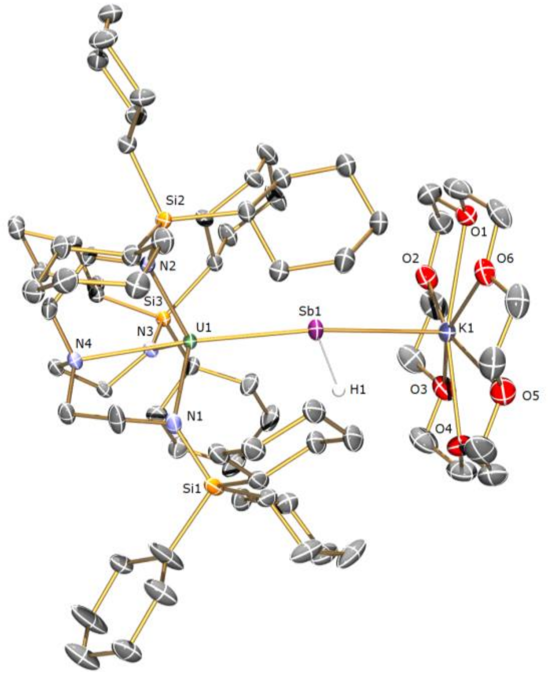

Supplementary Figure 8. Solid-state structure of complex **9UK** at 150 K. Thermal ellipsoids are set at 40% probability and hydrogen atoms, except H1, and lattice solvent are omitted for clarity. CCDC number 2404130.

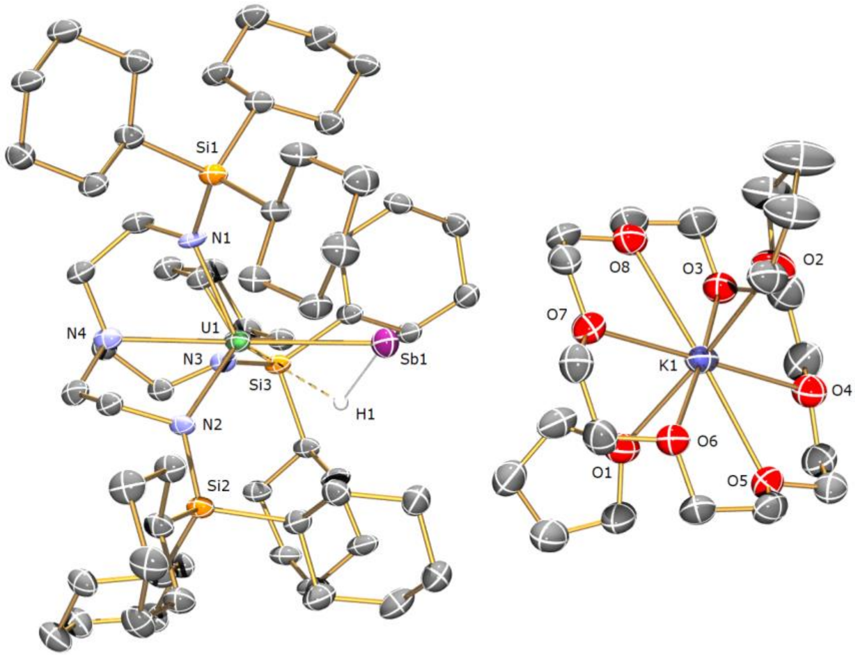

Supplementary Figure 9. Solid-state structure of complex **10UK** at 100 K. Thermal ellipsoids are set at 40% probability and hydrogen atoms, except H1, disordered components and lattice solvent are omitted for clarity. CCDC number 2404131.

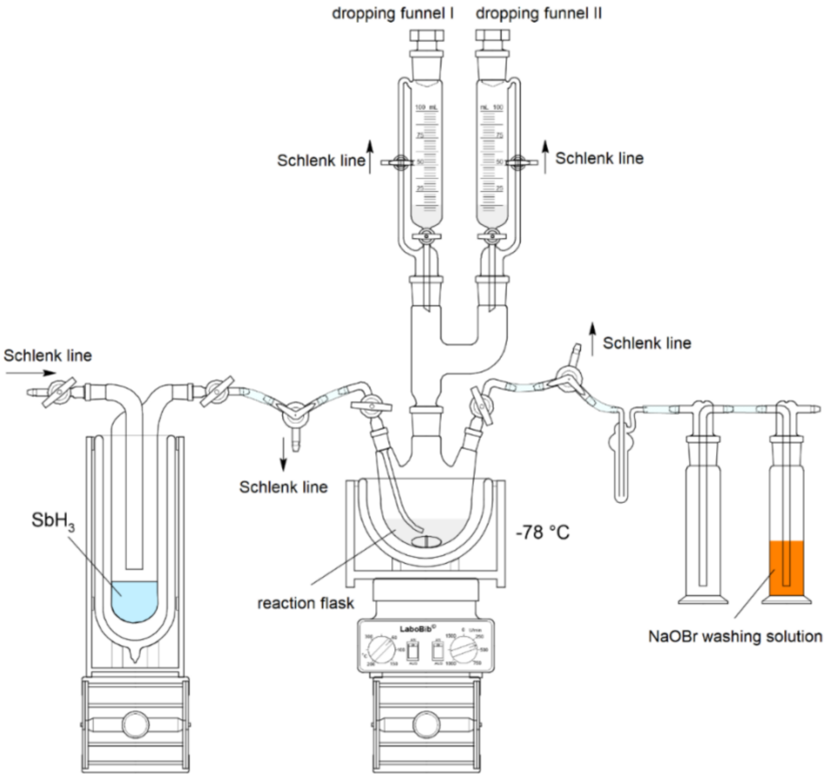

Supplementary Figure 10. Schematic illustration of the apparatus used for the synthesis of **1M** (M = Na, K).

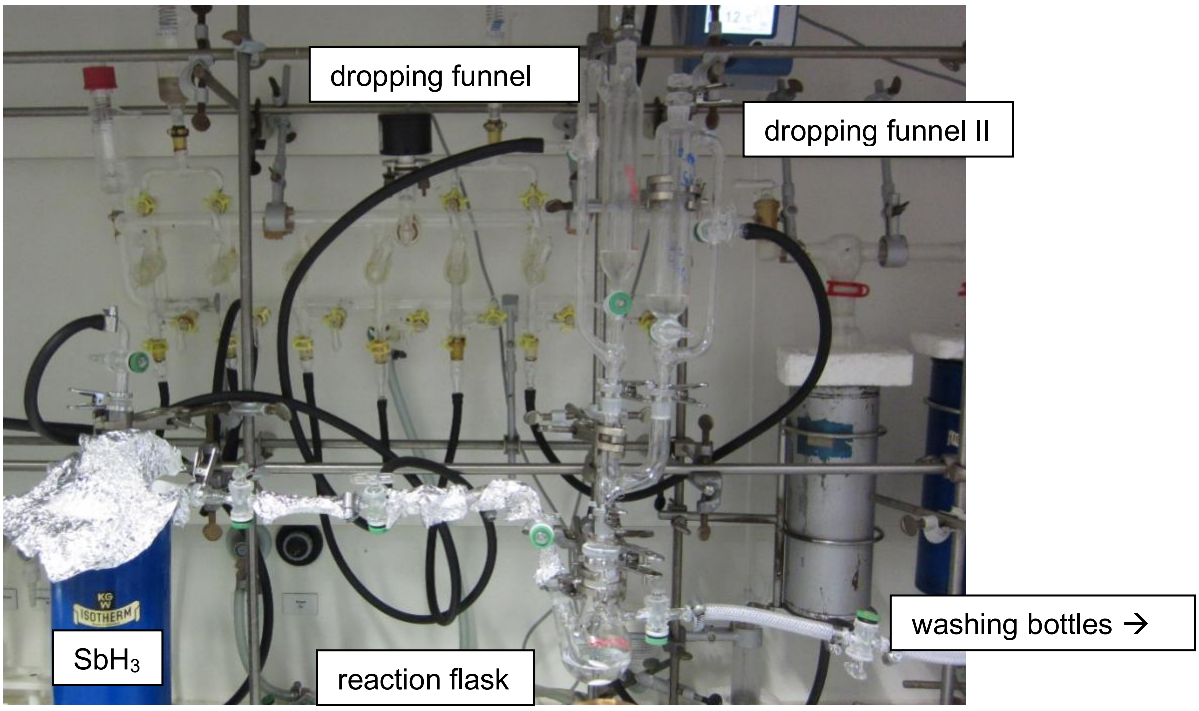

Supplementary Figure 11. Apparatus used for the synthesis of **1M** (M = Na, K).

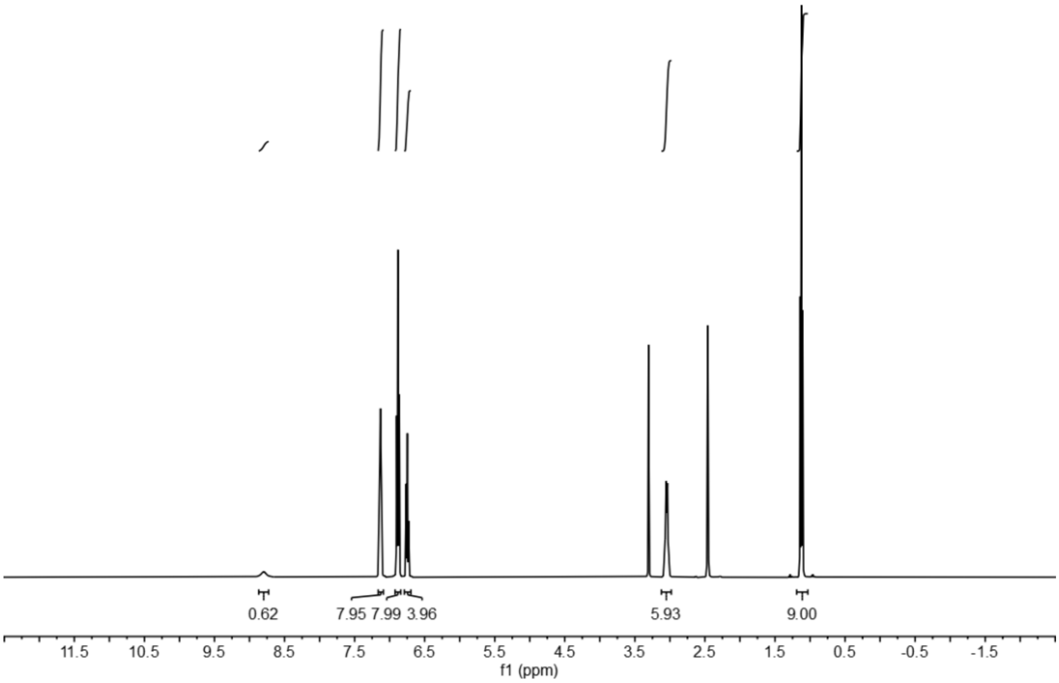

Supplementary Figure 12. ^{1}H NMR (D_6-DMSO, 298 K) spectrum of $[HNEt_3][BPh_4]$.

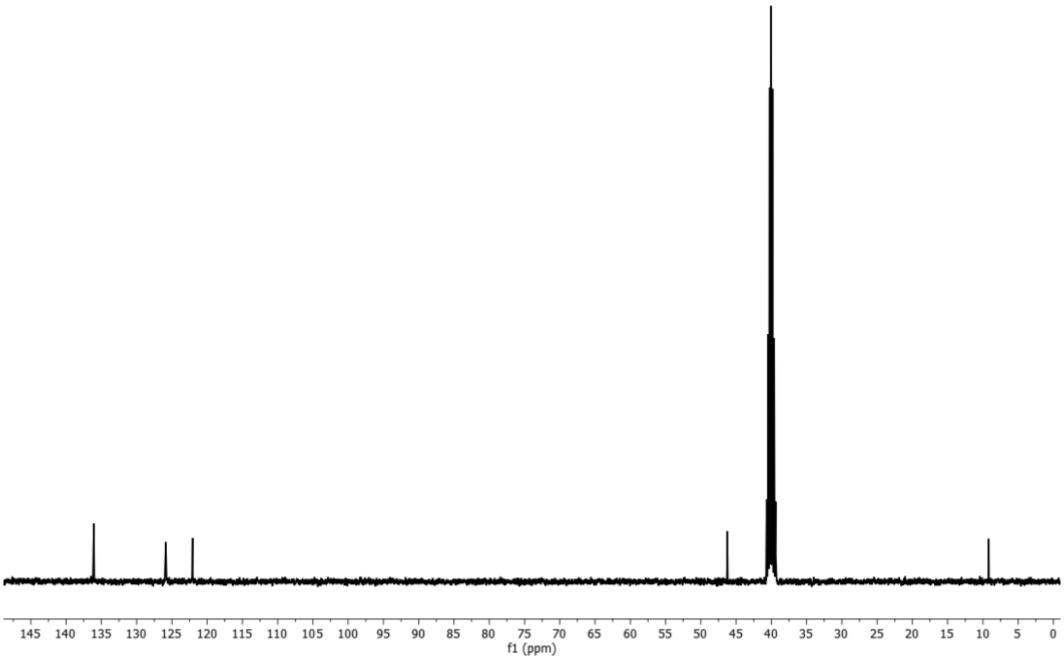

Supplementary Figure 13. $^{13}C\{^{1}H\}$ NMR (D_6-DMSO, 298 K) spectrum of $[HNEt_3][BPh_4]$.

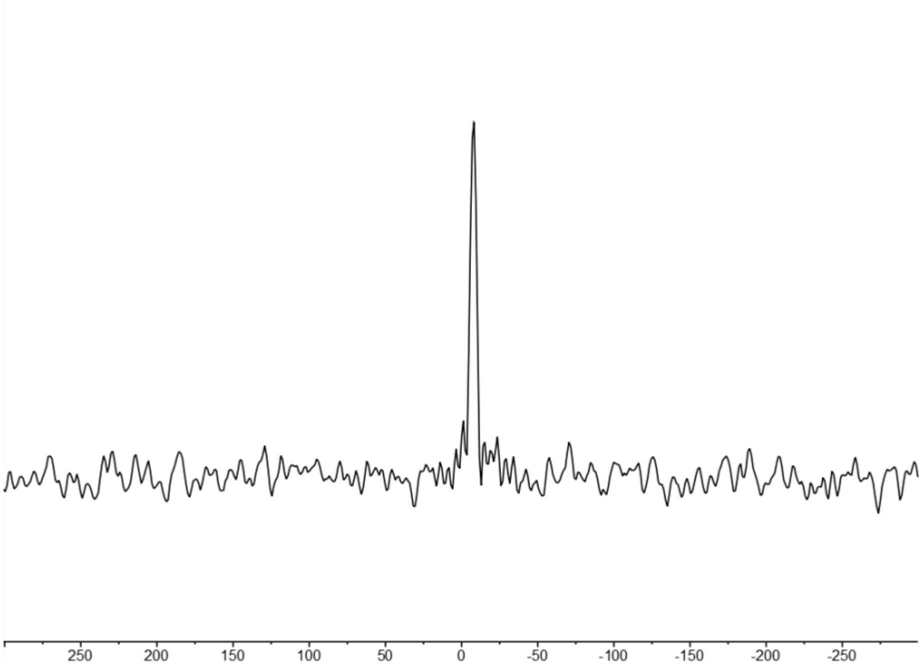

Supplementary Figure 14. ^{11}B NMR (D_6-DMSO, 298 K) spectrum of $[HNEt_3][BPh_4]$.

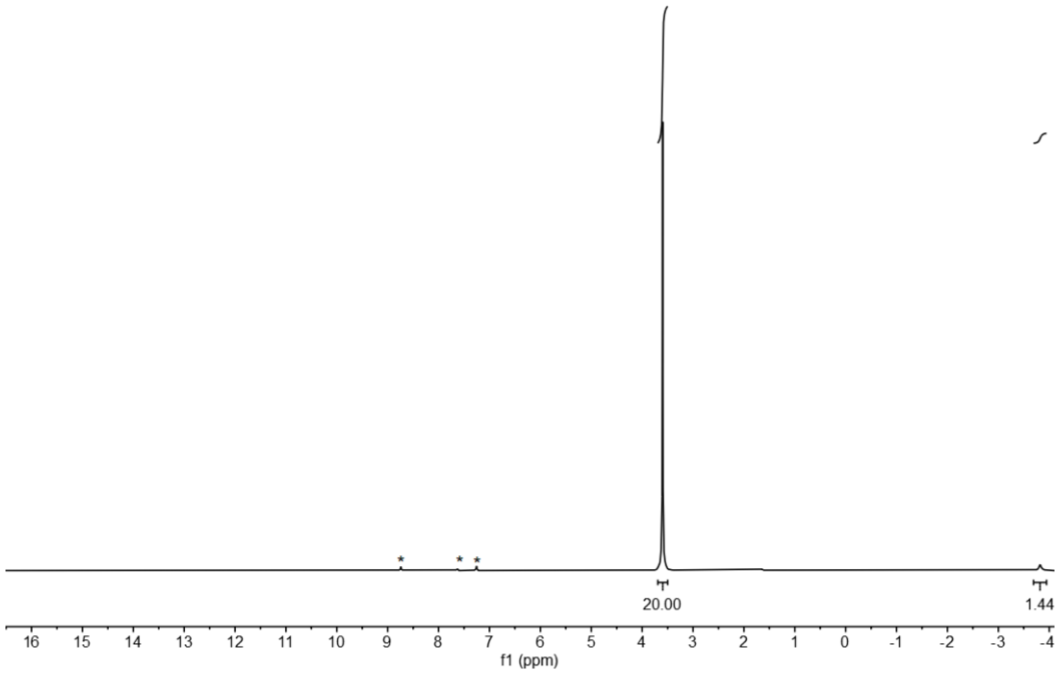

Supplementary Figure 15. ^{1}H NMR (D_5-pyridine, 298 K) of **1Na**. * = D_5-pyridine.

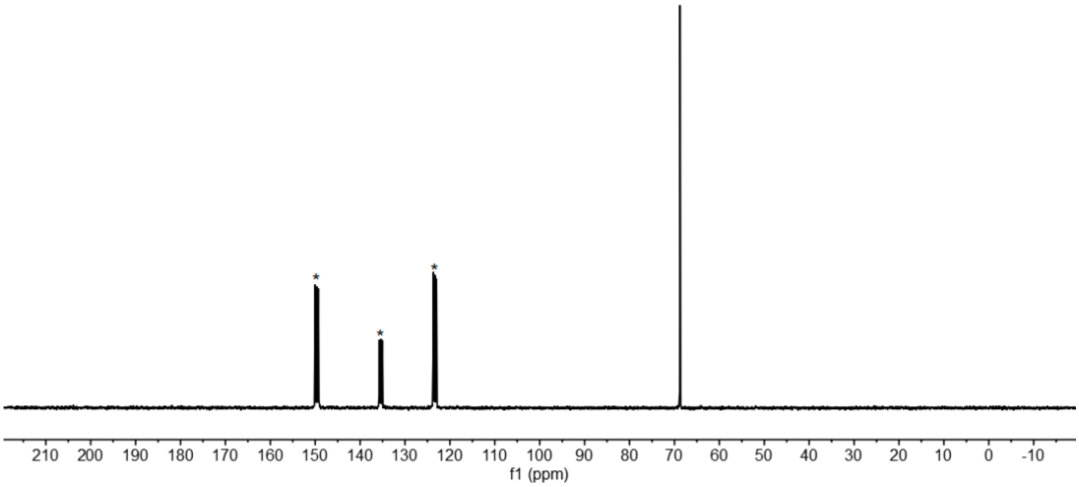

Supplementary Figure 16. $^{13}C\{^{1}H\}$ NMR (D_5-pyridine, 298 K) of **1Na**. * = D_5-pyridine.

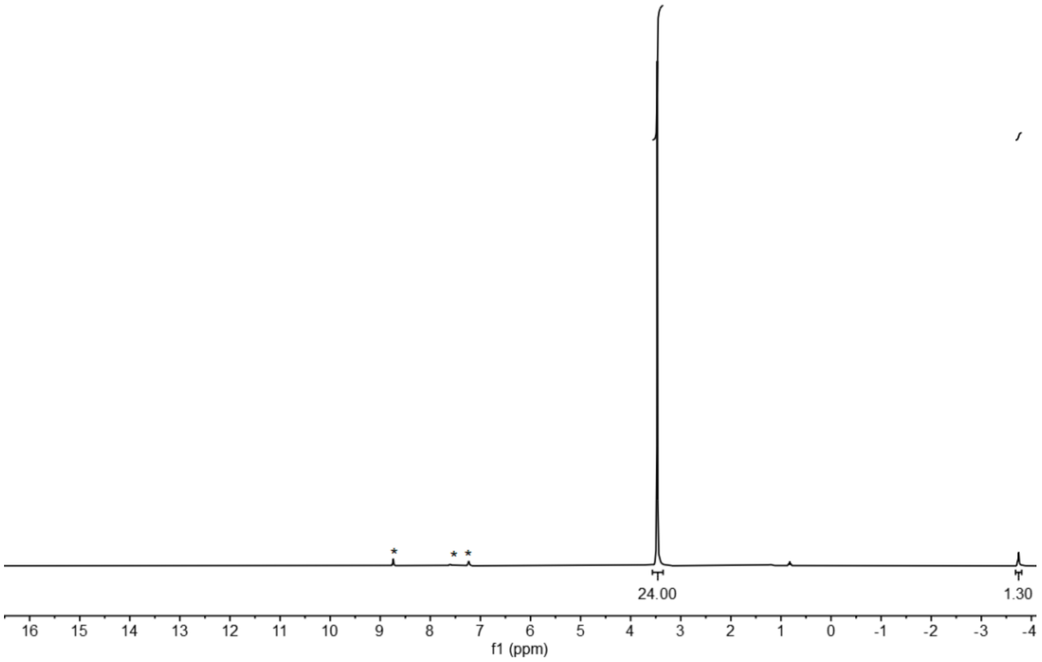

Supplementary Figure 17. ^{1}H NMR (D_5-pyridine, 298 K) of **1K**. * = D_5-pyridine.

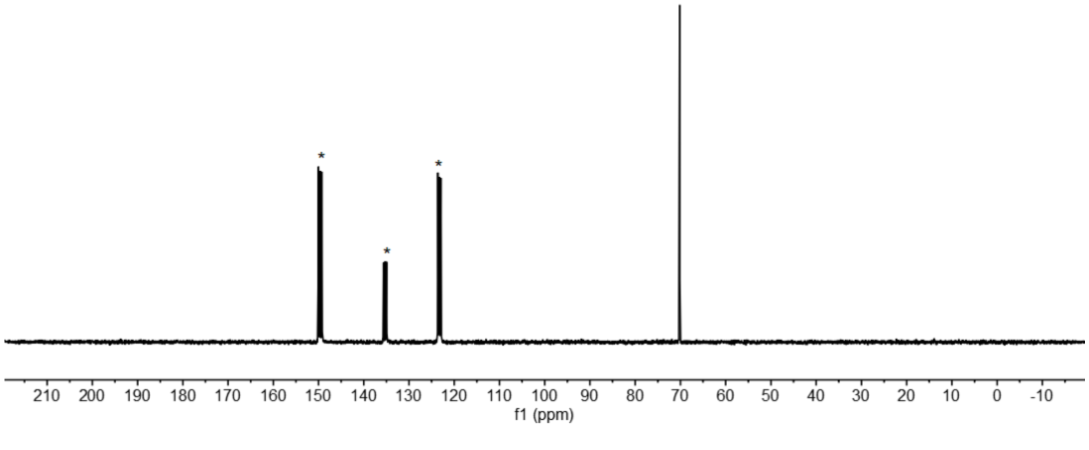

Supplementary Figure 18. ^{13}C{^{1}H} NMR (D_5-pyridine, 298 K) of **1K**. * = D_5-pyridine.

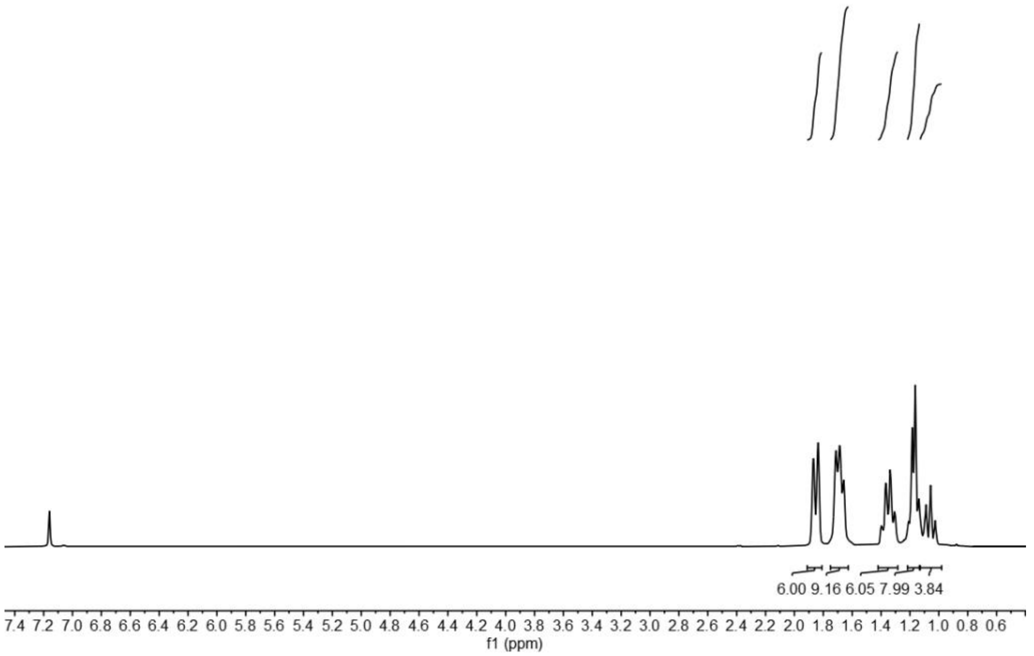

Supplementary Figure 19. ^{1}H NMR (C_6D_6, 298 K) spectrum of Cy_3SiCl.

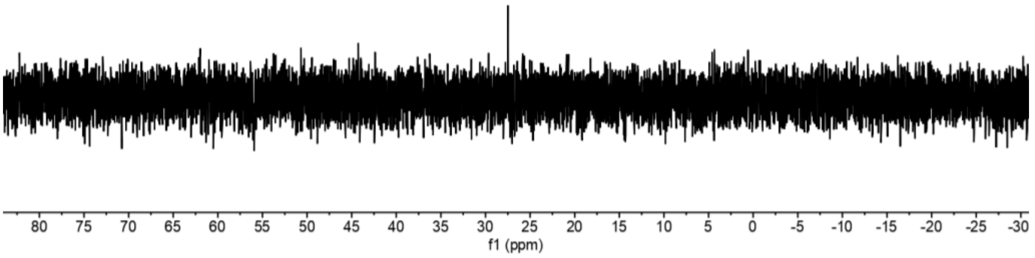

Supplementary Figure 20. ^{29}Si NMR (C_6D_6, 298 K) spectrum of Cy_3SiCl.

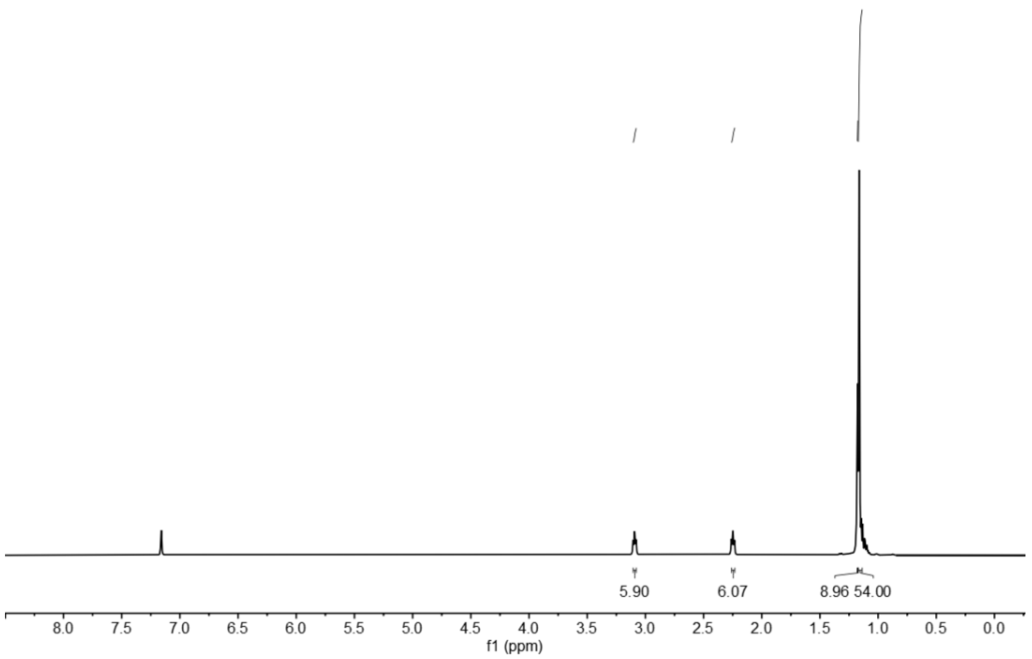

Supplementary Figure 21. ^{1}H NMR (C_6D_6, 298 K) spectrum of $Tren^{TIPS}Li_3$.

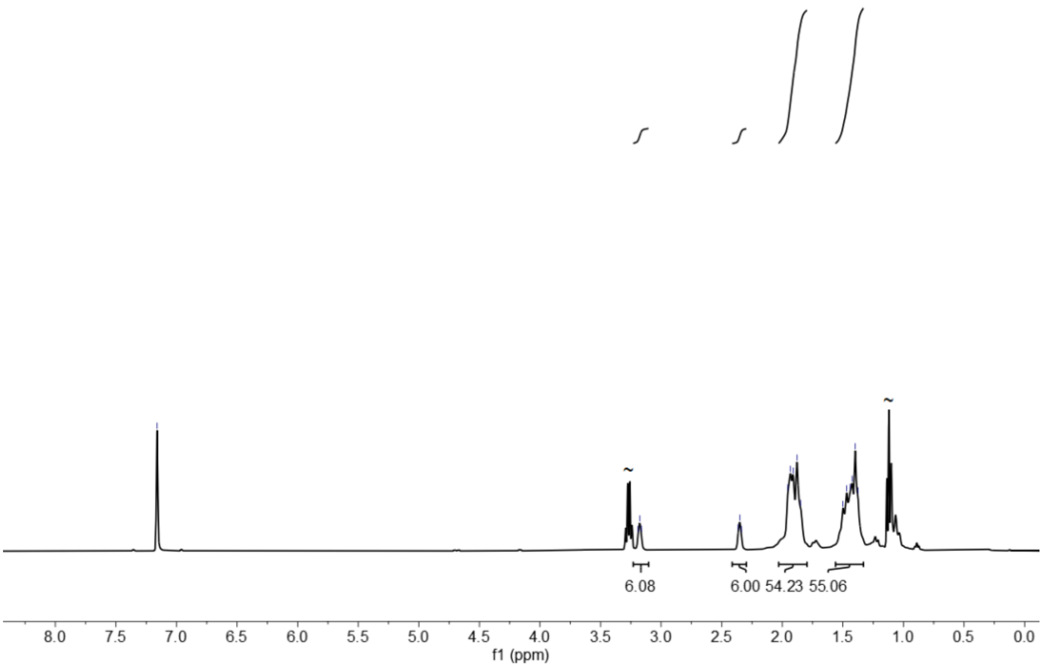

Supplementary Figure 22. ^{1}H NMR (C_6D_6, 298 K) spectrum of $Tren^{TCHS}Li_3$. ~ = diethyl ether (t, 1.11 ppm and q, 3.26 ppm).

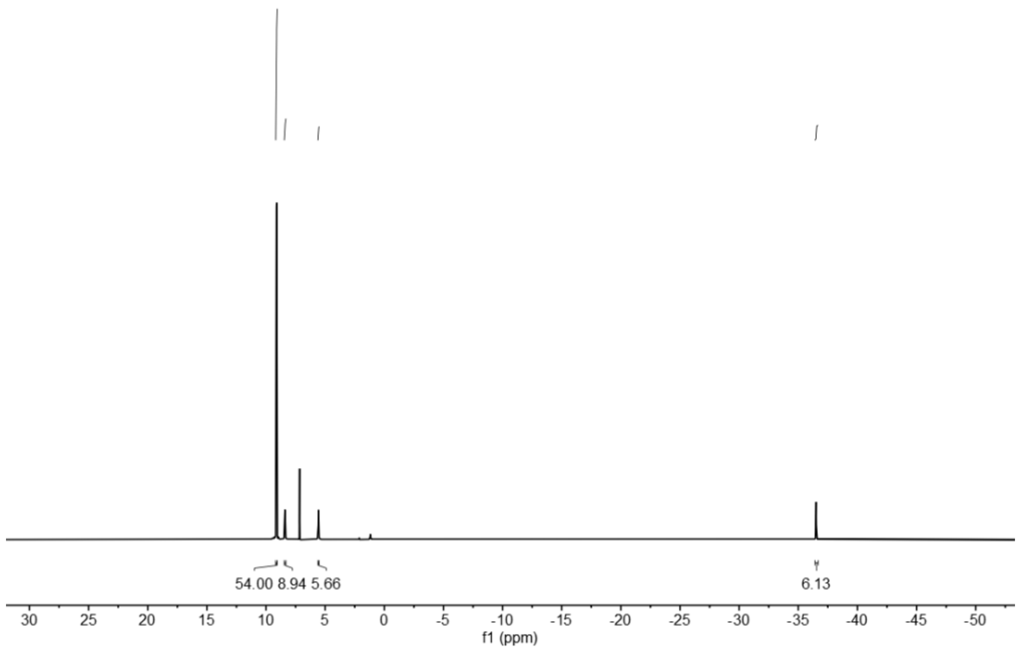

Supplementary Figure 23. ^{1}H NMR (C_6D_6, 298 K) spectrum of [U(Tren^{TIPS})Cl].

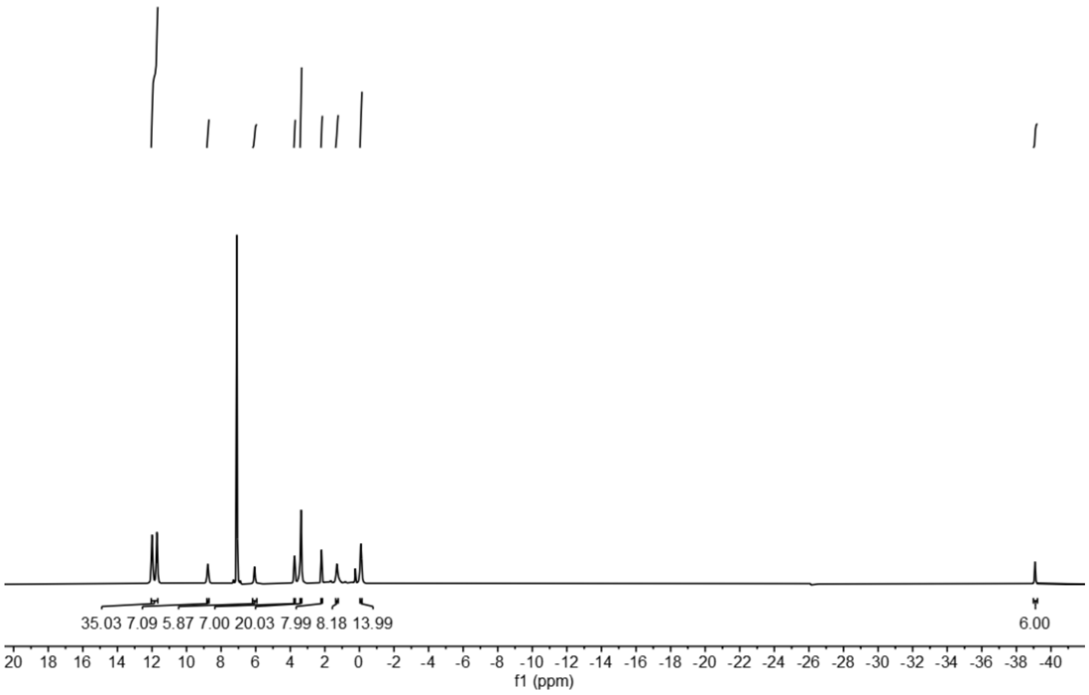

Supplementary Figure 24. ^{1}H NMR (C_6D_6, 298 K) spectrum of [U(Tren^{TCHS})Cl].

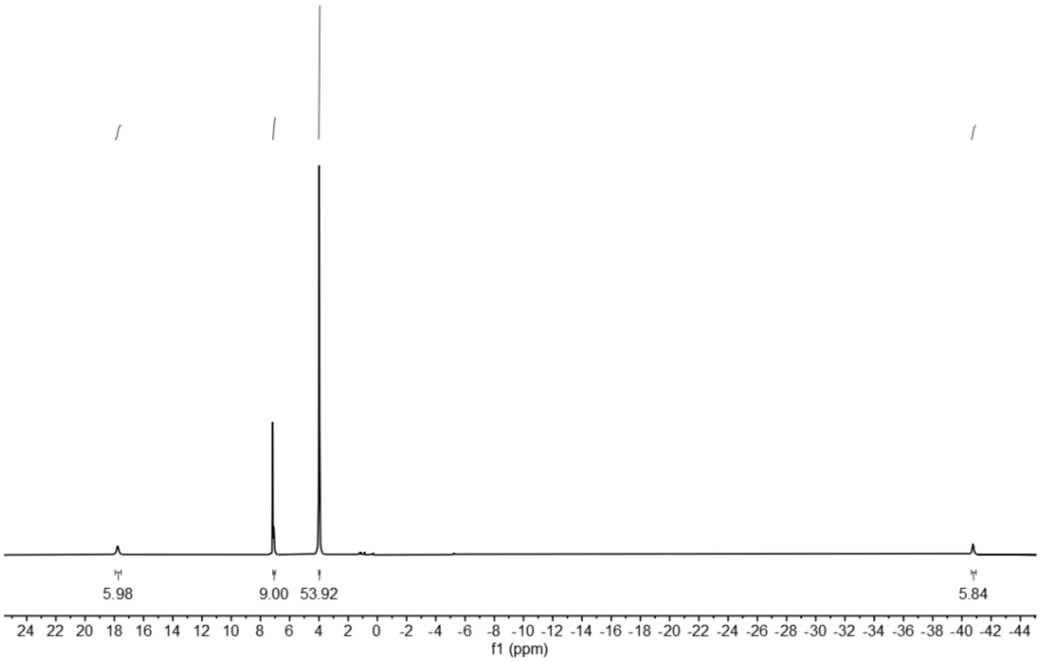

Supplementary Figure 25. ^{1}H NMR (C_6D_6, 298 K) spectrum of [U(Tren^{TIPS})].

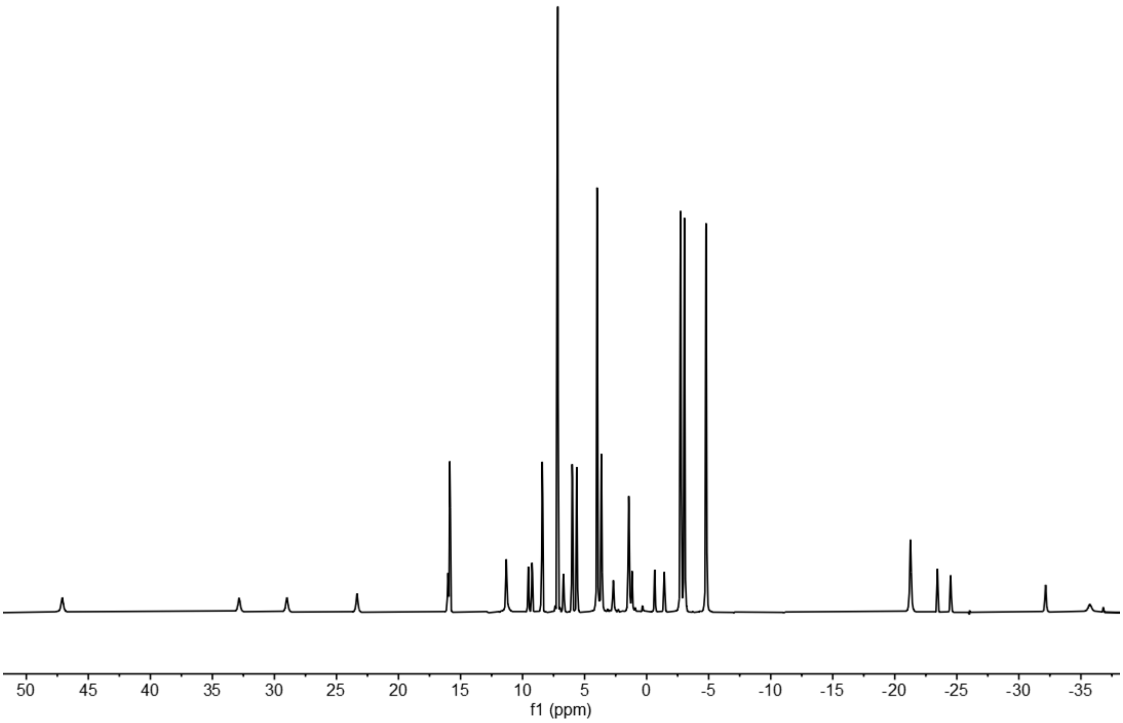

Supplementary Figure 26. ^{1}H NMR (C_6D_6, 298 K) spectrum of **2U**.

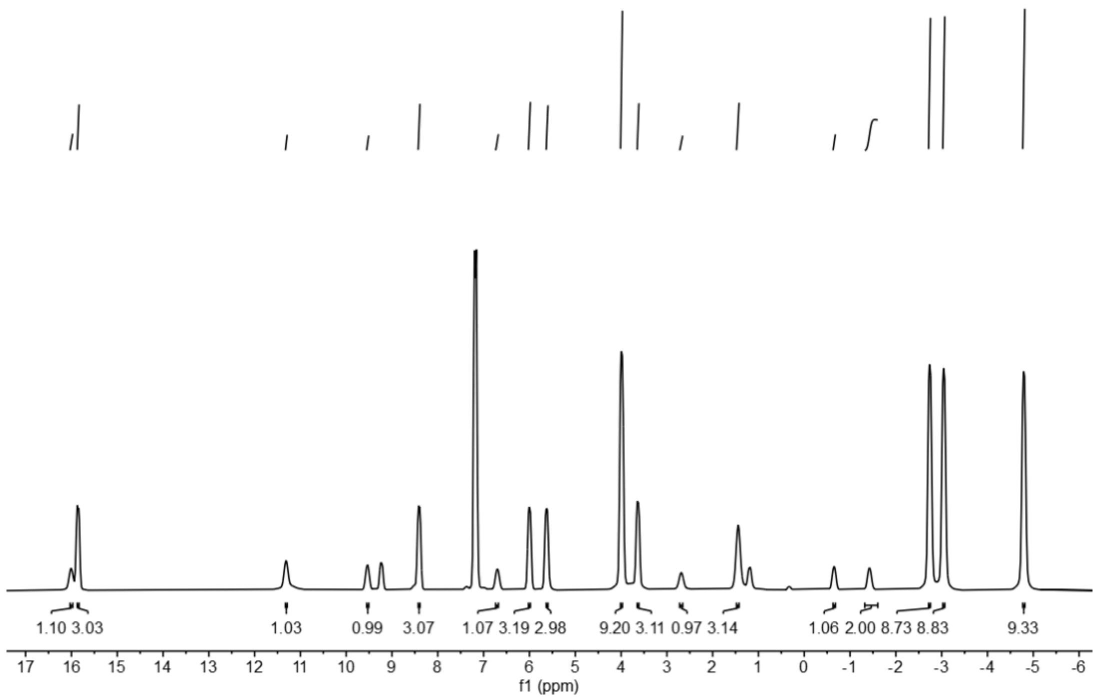

Supplementary Figure 27. Zoom-in of the ^{1}H NMR (C_6D_6, 298 K) spectrum of **2U**.

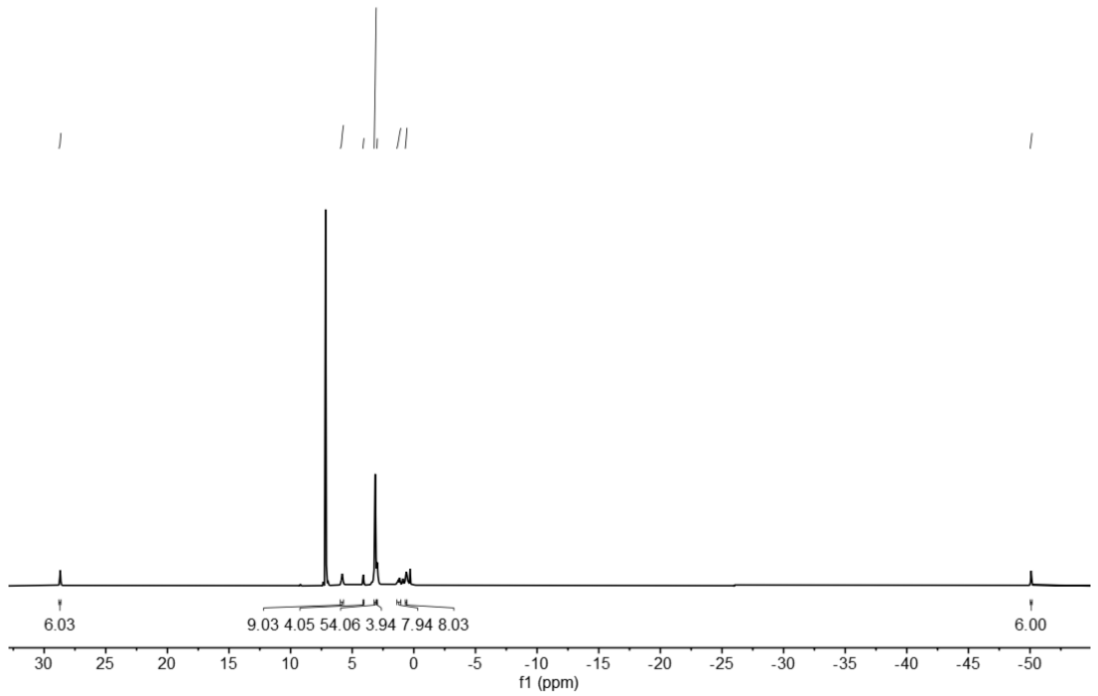

Supplementary Figure 28. ^{1}H NMR (C_6D_6, 298 K) spectrum of **4U**.

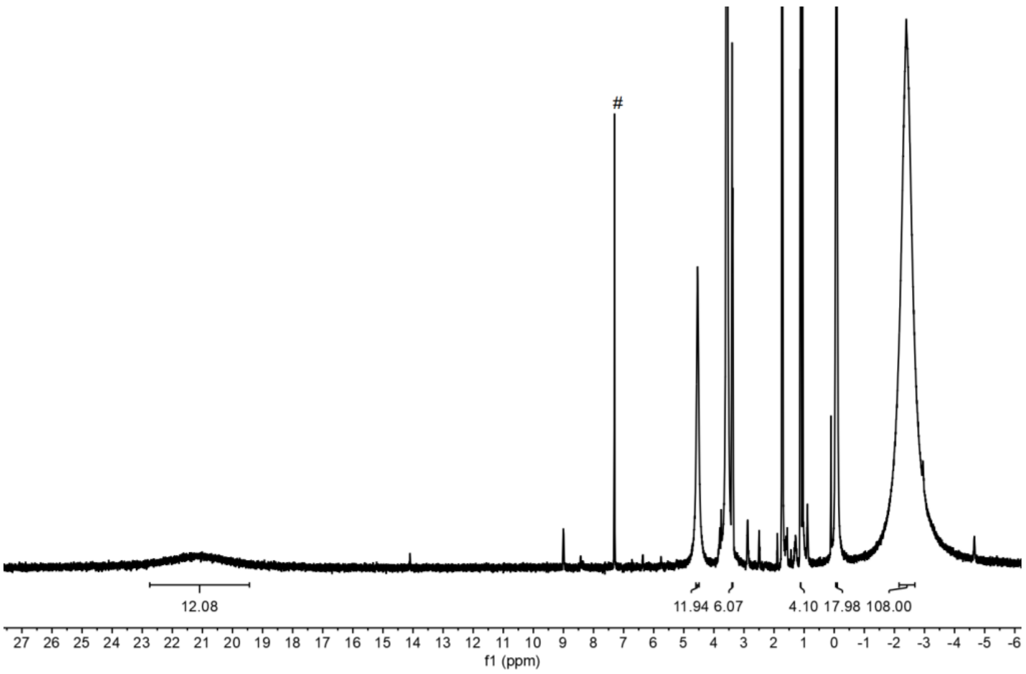

Supplementary Figure 29. ^{1}H NMR (D$_8$-THF, 298 K) spectrum of **5UNa**. # = benzene (s, 7.31 ppm). The resonances attributed to the 20 hydrogens of 15C5 could not be definitively assigned due to overlapping with resonances from the D$_8$-THF solvent used for the NMR experiments.

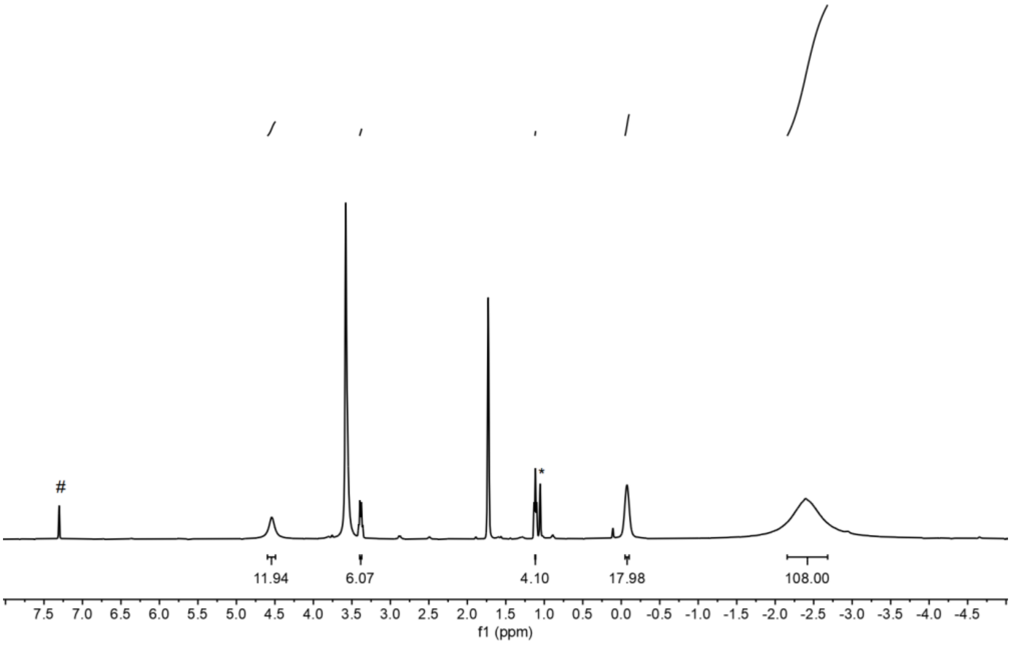

Supplementary Figure 30. Zoom-in of the ^{1}H NMR (D$_8$-THF, 298 K) spectrum of **5UNa**. * = trace Tren^{TIPS}H$_3$; # = benzene (s, 7.31 ppm). The resonances attributed to the 20 hydrogens of 15C5 could not be definitively assigned due to overlapping with resonances from the D$_8$-THF solvent used for the NMR experiments.

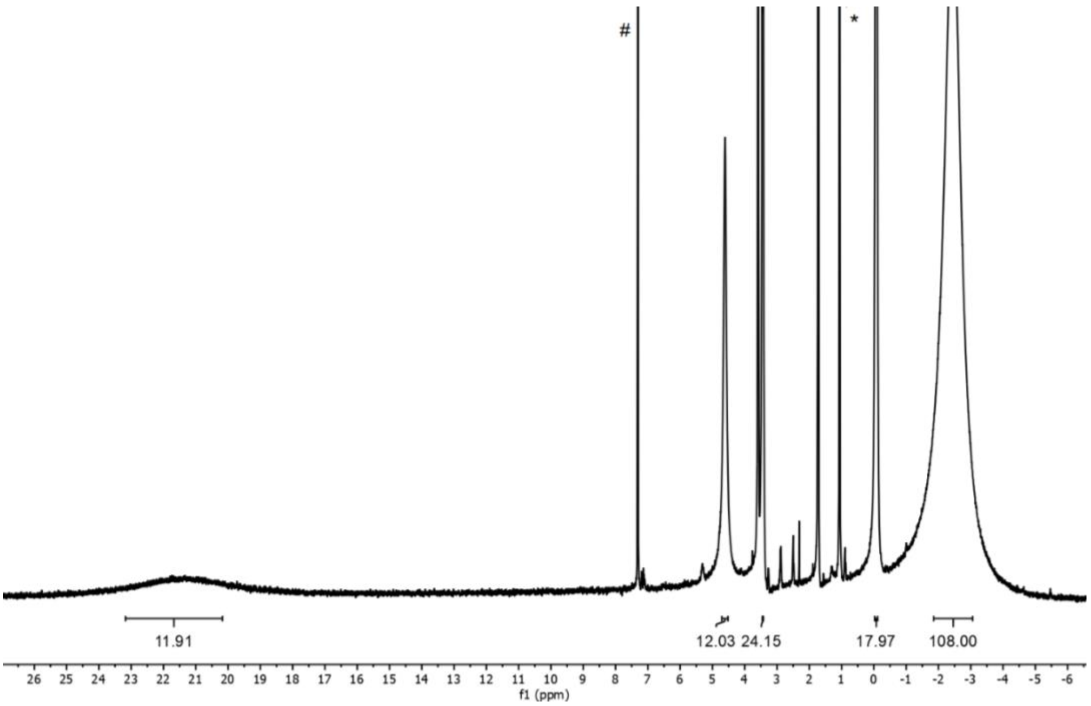

Supplementary Figure 31. ^{1}H NMR (D_8-THF, 298 K) spectrum of **5UK**. * = trace $Tren^{TIPS}H_3$; # = benzene (s, 7.31 ppm).

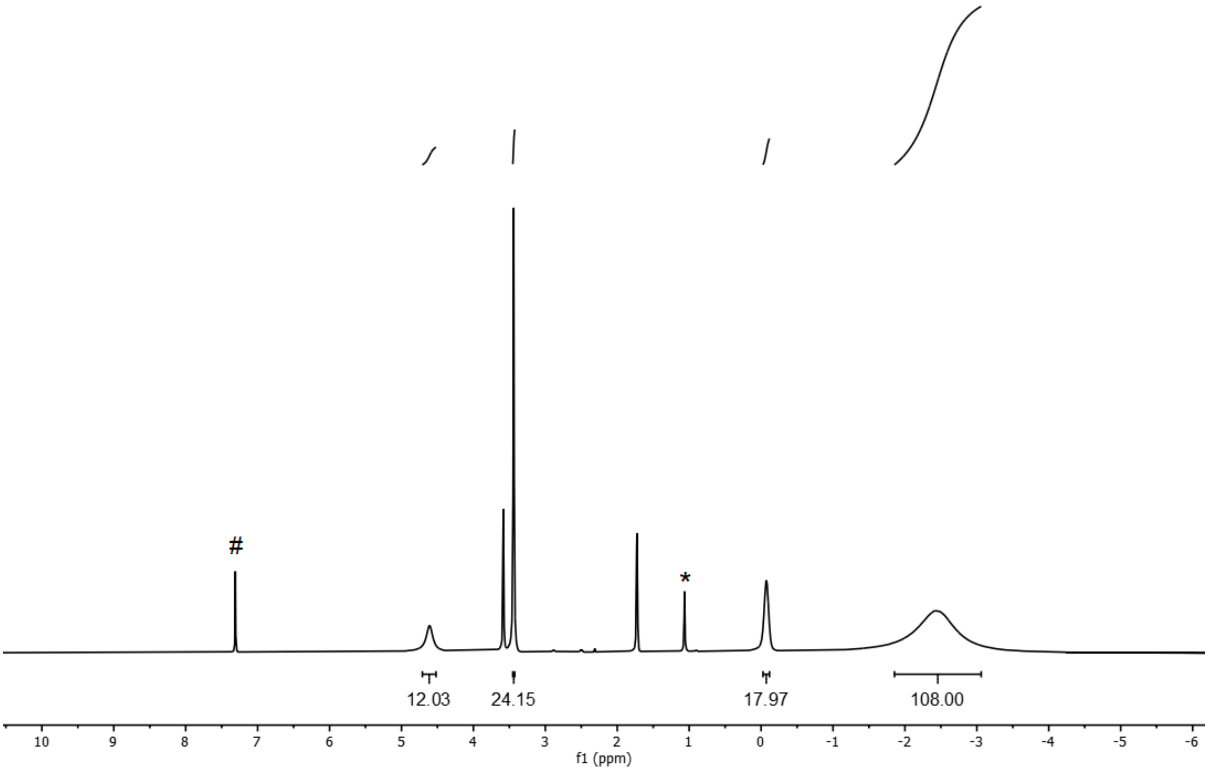

Supplementary Figure 32. Zoom-in of the ^{1}H NMR (D_8-THF, 298 K) spectrum of **5UK.** * = trace $Tren^{TIPS}H_3$; # = benzene (s, 7.31 ppm).

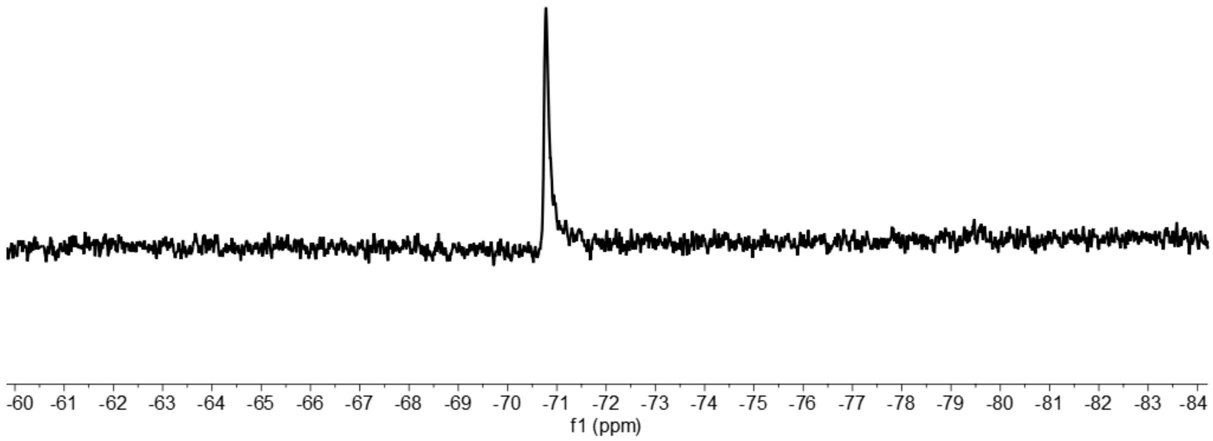

Supplementary Figure 33. $^{29}Si\{^{1}H\}$ NMR (D_8-THF, 298 K) spectrum of **5UK**.

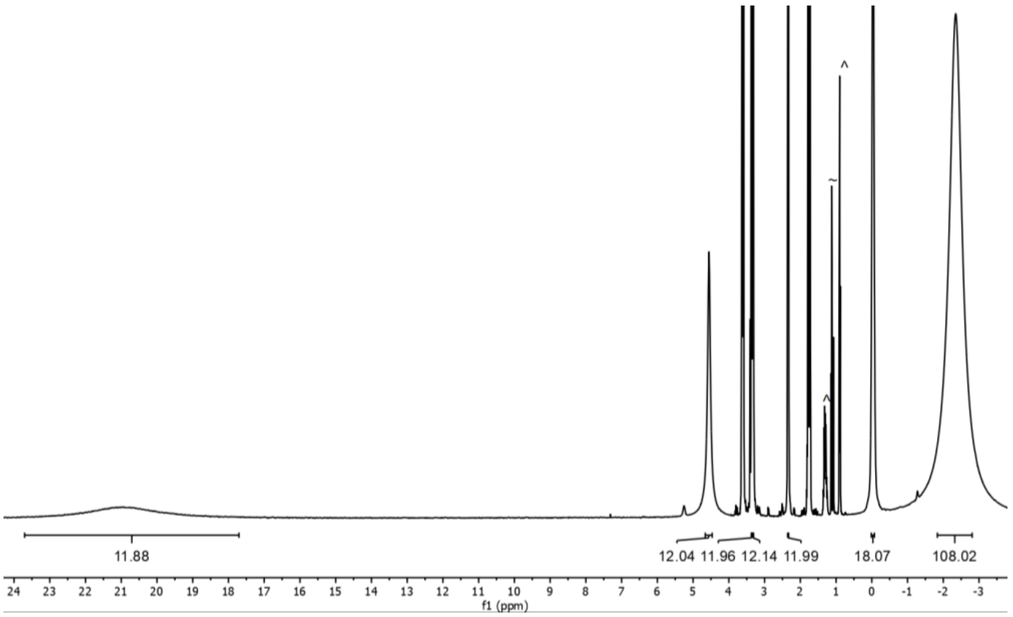

Supplementary Figure 34. ^{1}H NMR (D_8-THF, 298 K) spectrum of **5UK'.** ~ = trace diethyl ether (t, 1.11 ppm and q, 3.26 ppm); ^ = trace pentane (t, ~0.89 ppm and q, ~1.31 ppm). The presence of diethyl ether and pentane results from their unsuccessful use as crystallisation solvents.

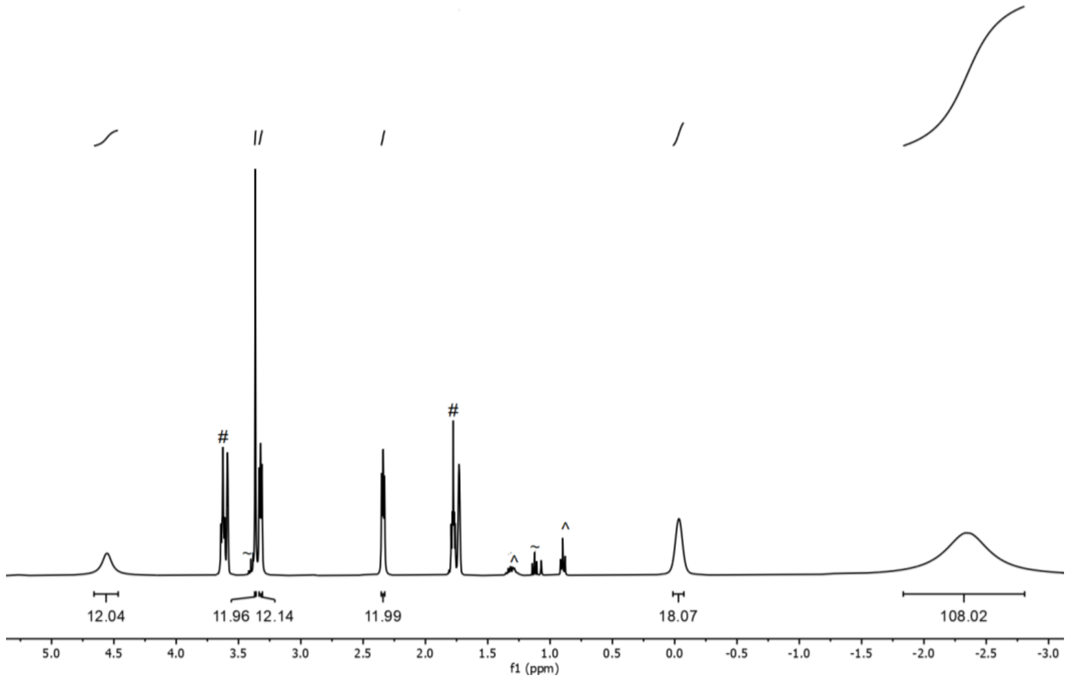

Supplementary Figure 35. Zoom-in of the ^{1}H NMR (D_8-THF, 298 K) spectrum of **5UK'**. # = THF (m, 1.79 ppm and m, 3.62 ppm); ~ = trace diethyl ether (t, 1.11 ppm and q, 3.26 ppm); ^ = trace pentane (t, ~ 0.89 ppm and q, ~1.31 ppm). The presence of diethyl ether and pentane results from their unsuccessful use as crystallisation solvents.

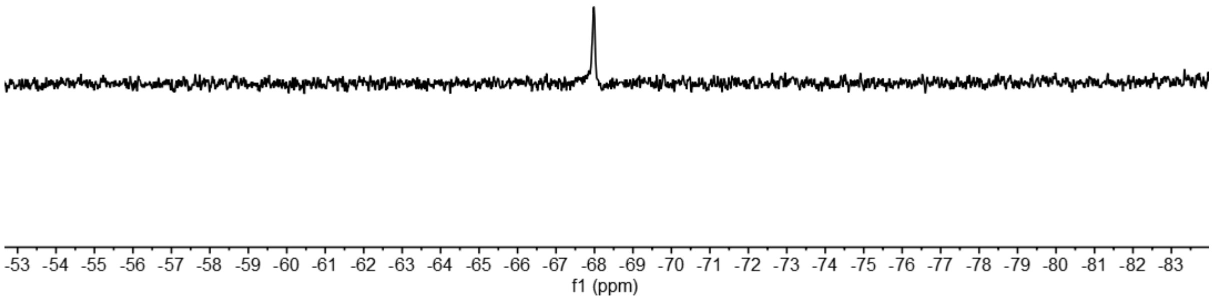

Supplementary Figure 36. $^{29}Si\{^{1}H\}$ NMR (D_8-THF, 298 K) spectrum of **5UK'**.

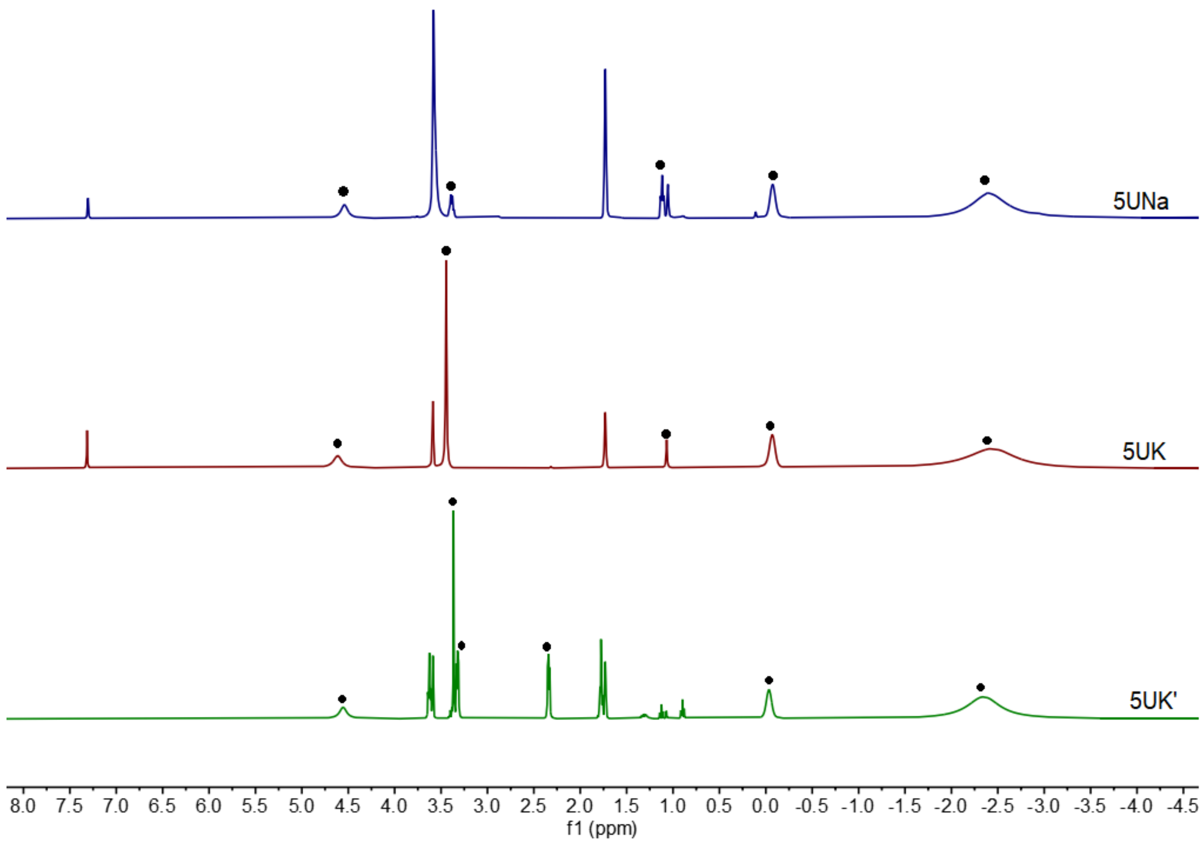

Supplementary Figure 37. Stacked ^{1}H NMR (D_8-THF, 298 K) of **5UNa** (blue, top), **5UK** (red, middle), and **5UK'** (green, bottom). Black spots (•) correspond to resonances attributed to **5UNa**, **5UK**, and **5UK'**.

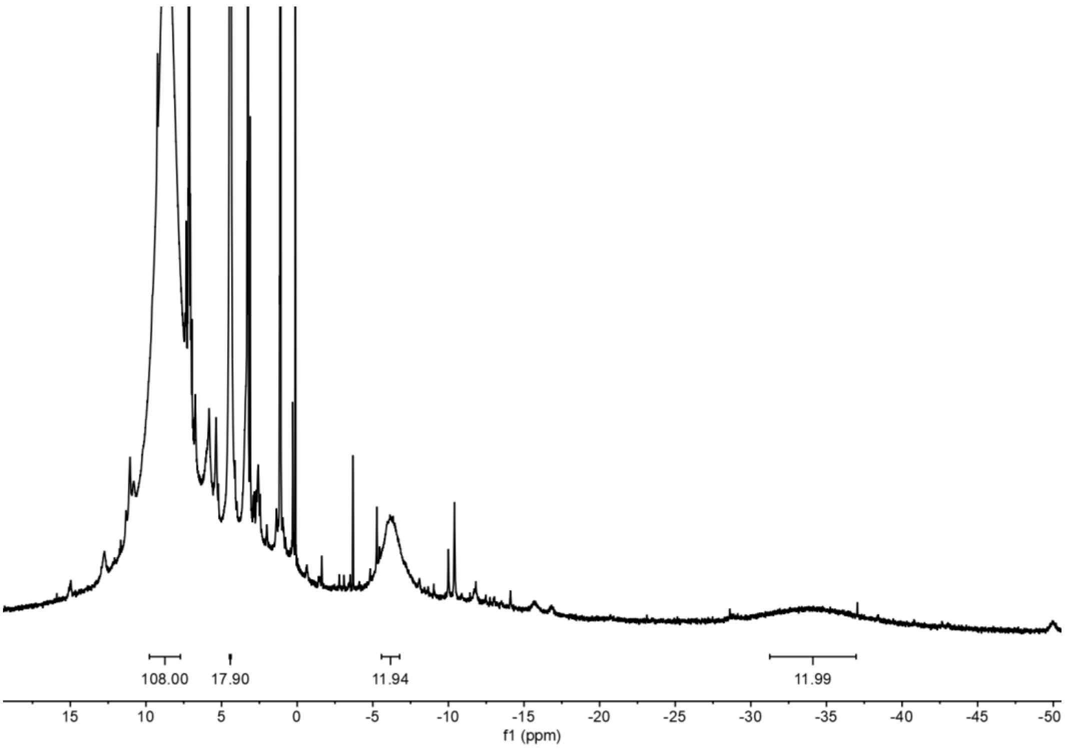

Supplementary Figure 38. ^{1}H NMR (C_6D_6, 298 K) spectrum of 11:89 **6U**:**7U**.

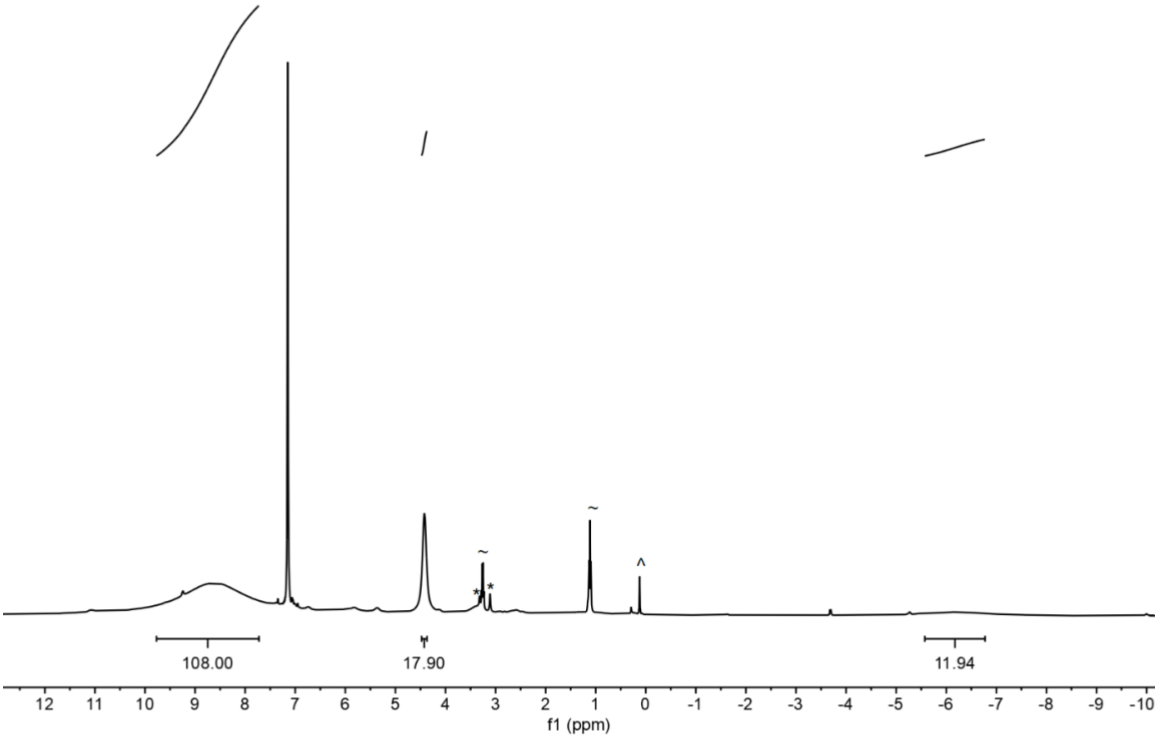

Supplementary Figure 39. Zoom-in of the ^{1}H NMR (C_6D_6, 298 K) spectrum of 11:89 **6U**:**7U**. ~ = trace diethyl ether (t, 1.11 ppm and q, 3.26 ppm); ^ = trace HMDSO (s, 0.12 ppm), * = trace DME (s, 3.12 ppm and s, 3.33 ppm). The presence of diethyl ether, HMDSO and DME results from their unsuccessful use as crystallisation solvents.

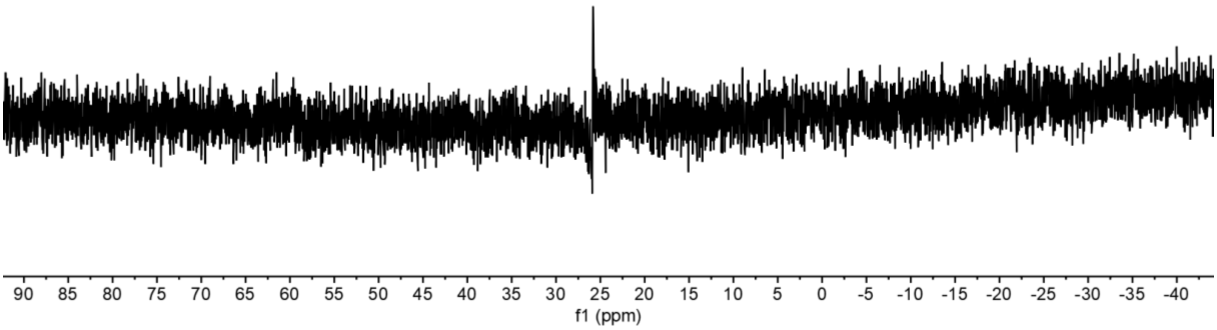

Supplementary Figure 40. $^{29}Si\{^1H\}$ NMR (C_6D_6, 298 K) spectrum of 11:89 **6U**:**7U**.

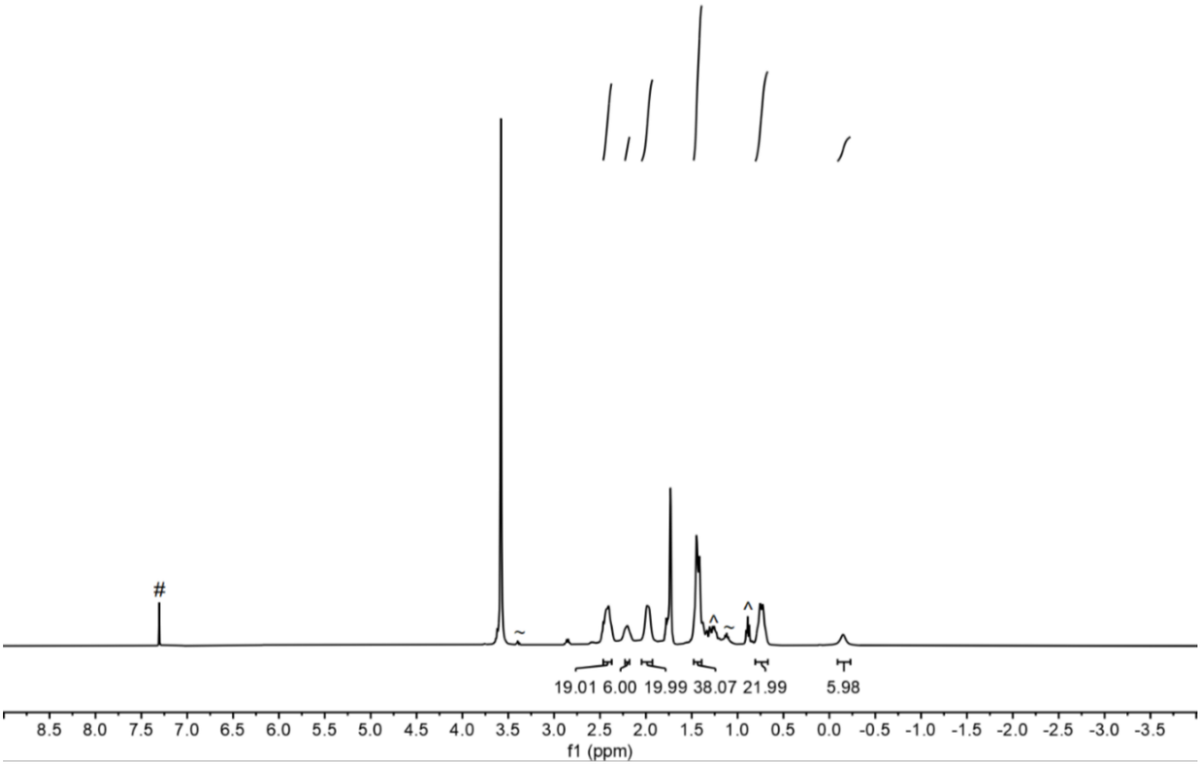

Supplementary Figure 41. ^1H NMR (D_8-THF, 298 K) spectrum of **10UK**. # = benzene (s, 7.31 ppm); ~ = trace diethyl ether (t, 1.11 ppm and q, 3.26 ppm); ^ = trace pentane (t, ~ 0.89 ppm and q, ~1.31 ppm). The presence of diethyl ether and pentane results from their unsuccessful use as crystallisation solvents.

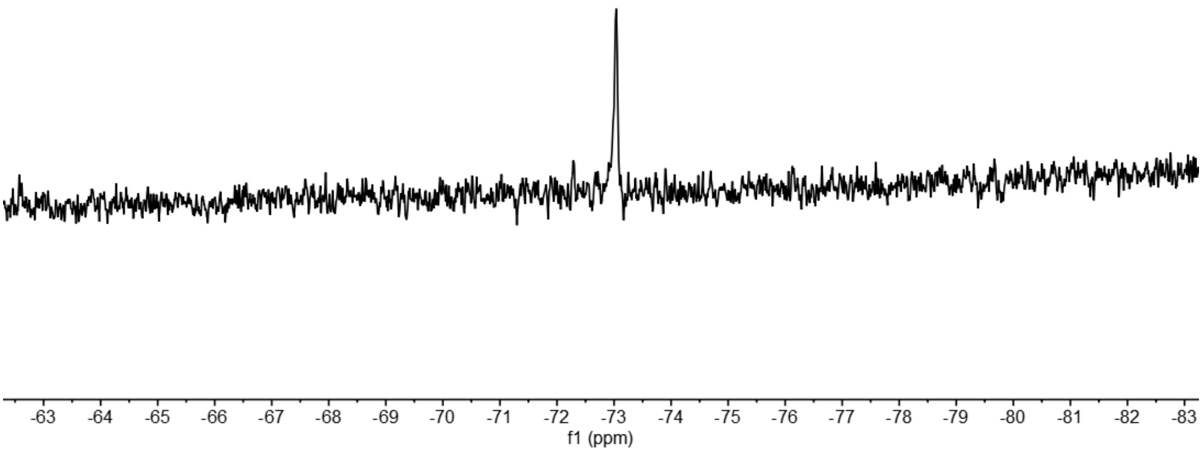

Supplementary Figure 42. $^{29}Si\{^{1}H\}$ NMR (D_8-THF, 298 K) spectrum of **10UK**.

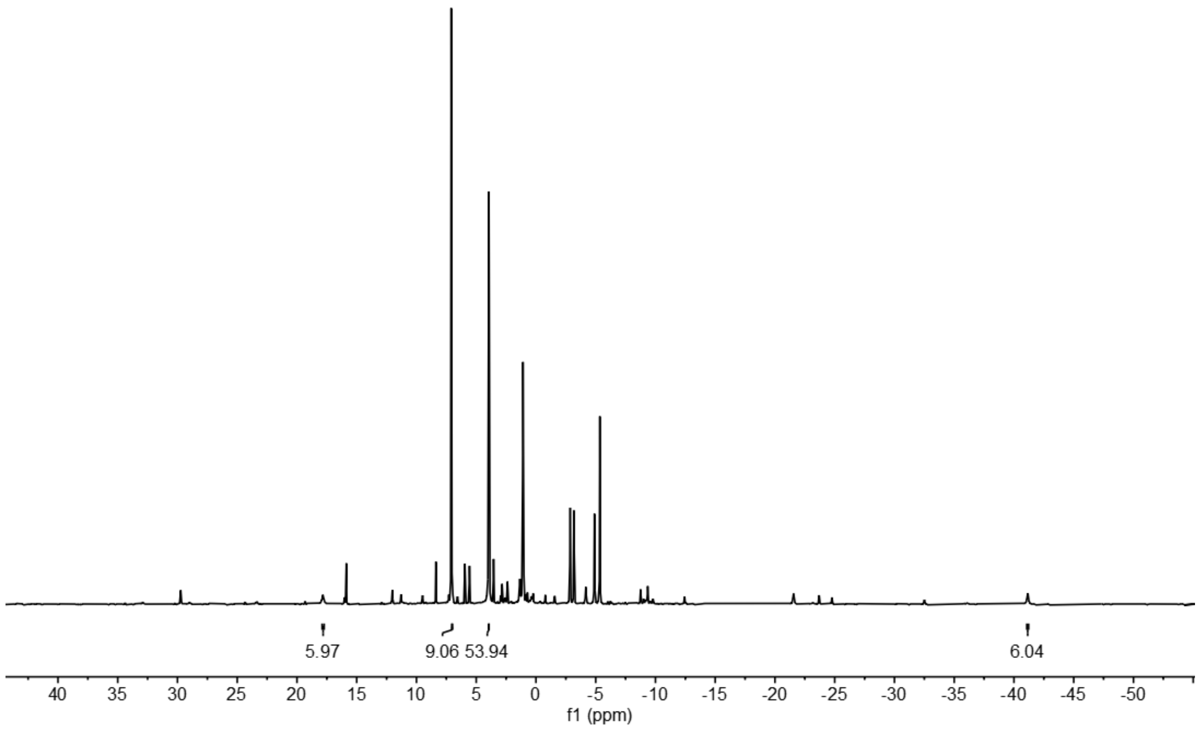

Supplementary Figure 43. ^{1}H NMR (C_6D_6, 298 K) spectrum of the crude reaction mixture resulting from the reduction of **4U**. Integrated resonances represent the four ^{1}H environments of [U(Tren^{TIPS})]: 17.67 (br, s, 6H, NCH_2CH$_2$), 7.12 (br, 9H, CH(CH$_3$)$_2$), 4.03 (s, 54H, CH(CH_3)$_2$), –40.09 (br, s, 6H, NCH$_2$CH_2) ppm.

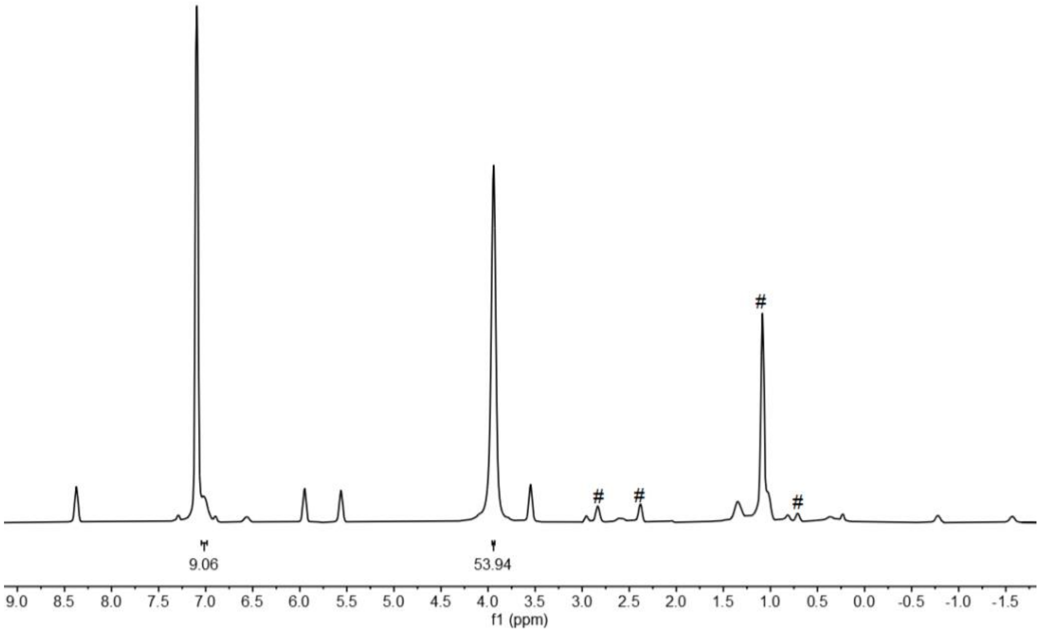

Supplementary Figure 44. Zoom-in of the ^{1}H NMR (C_6D_6, 298 K) spectrum of the crude reaction mixture resulting from the reduction of **4U**. Integrated resonances represent two of the four ^{1}H environments of [U(Tren^{TIPS})]: 7.12 (br, 9H, C*H*(CH_3)$_2$), 4.03 (s, 54H, CH(C*H*$_3$)$_2$) ppm. # = Tren^{TIPS}H_3.

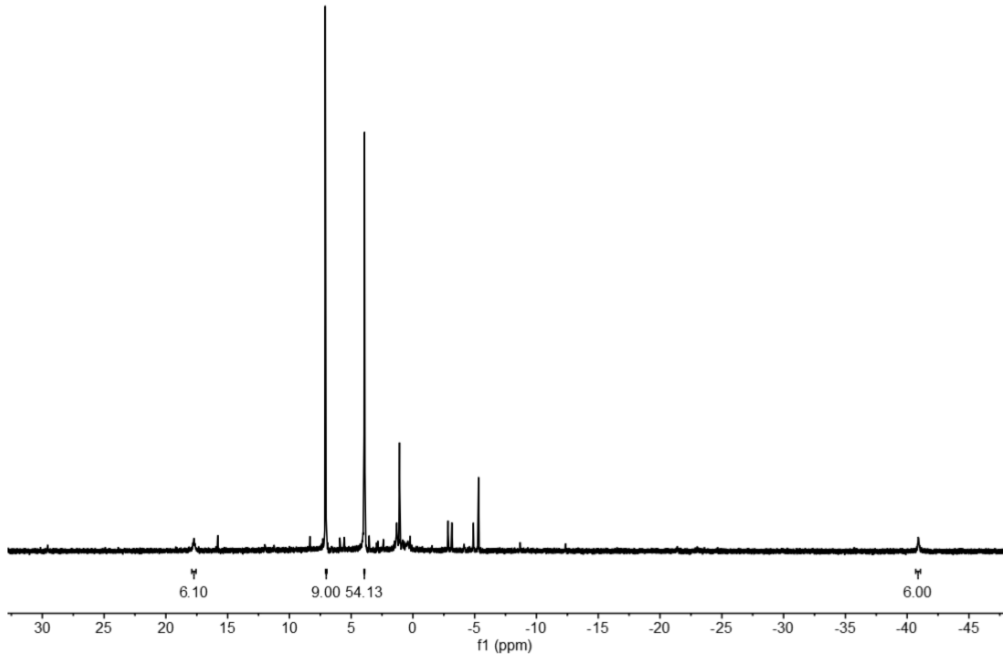

Supplementary Figure 45. ^{1}H NMR (C_6D_6, 298 K) spectrum of the crude reaction mixture resulting from the reduction of [U(Tren^{TIPS})]. Integrated resonances represent the four ^{1}H environments of [U(Tren^{TIPS})]: 17.67 (br, s, 6H, NC*H*$_2$CH$_2$), 7.12 (br, 9H, C*H*(CH_3)$_2$), 4.03 (s, 54H, CH(C*H*$_3$)$_2$), -40.09 (br, s, 6H, NCH$_2$C*H*$_2$) ppm.

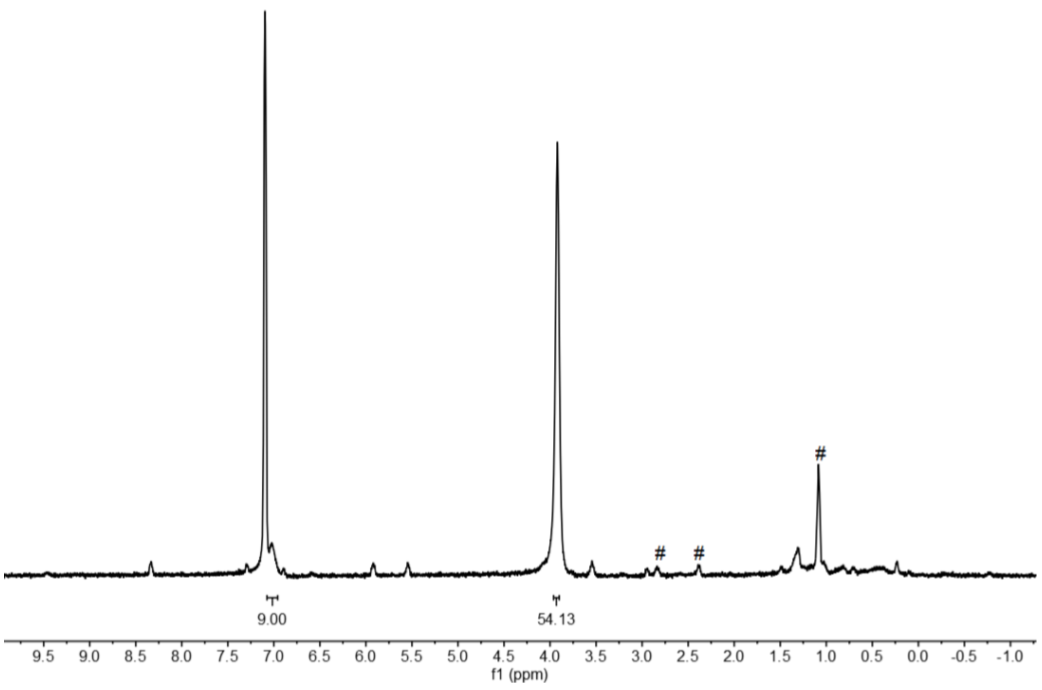

Supplementary Figure 46. Zoom-in of the ^{1}H NMR (C_6D_6, 298 K) spectrum of the crude reaction mixture resulting from the reduction of [U(Tren^{TIPS})]. Integrated resonances represent two of the four ^{1}H environments of [U(Tren^{TIPS})]: 7.12 (br, 9H, C*H*(CH_3)$_2$), 4.03 (s, 54H, CH(C*H*$_3$)$_2$) ppm. # = Tren^{TIPS}H$_3$.

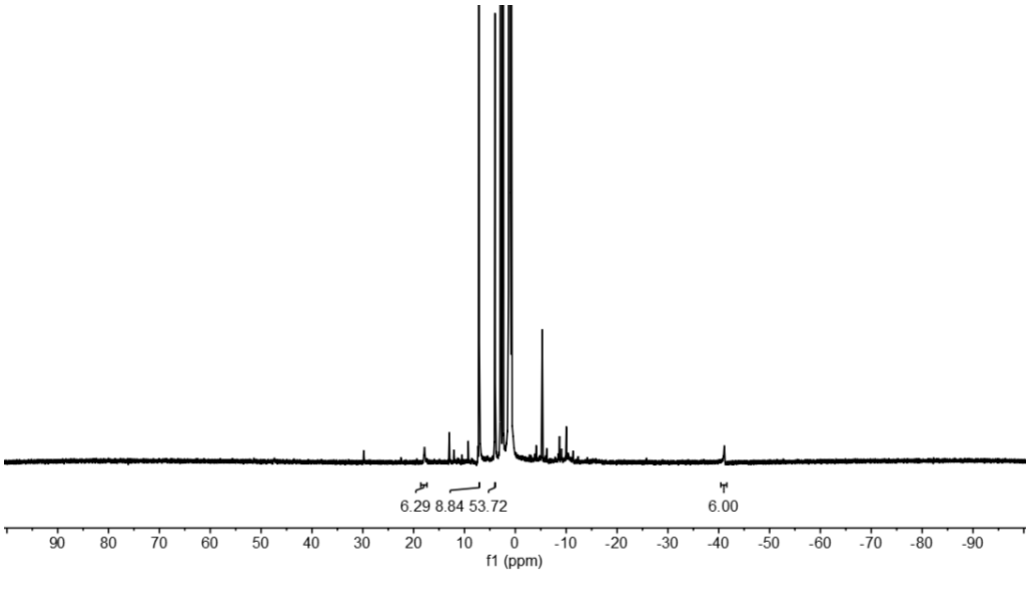

Supplementary Figure 47. ^{1}H NMR (C_6D_6, 298 K) spectrum of the crude reaction mixture resulting from the reduction of **6U**. Integrated resonances represent the four ^{1}H environments of [U(Tren^{TIPS})]: 17.67 (br, s, 6H, NC*H*$_2$CH$_2$), 7.12 (br, 9H, C*H*(CH_3)$_2$), 4.03 (s, 54H, CH(C*H*$_3$)$_2$), -40.09 (br, s, 6H, NCH$_2$C*H*$_2$) ppm.

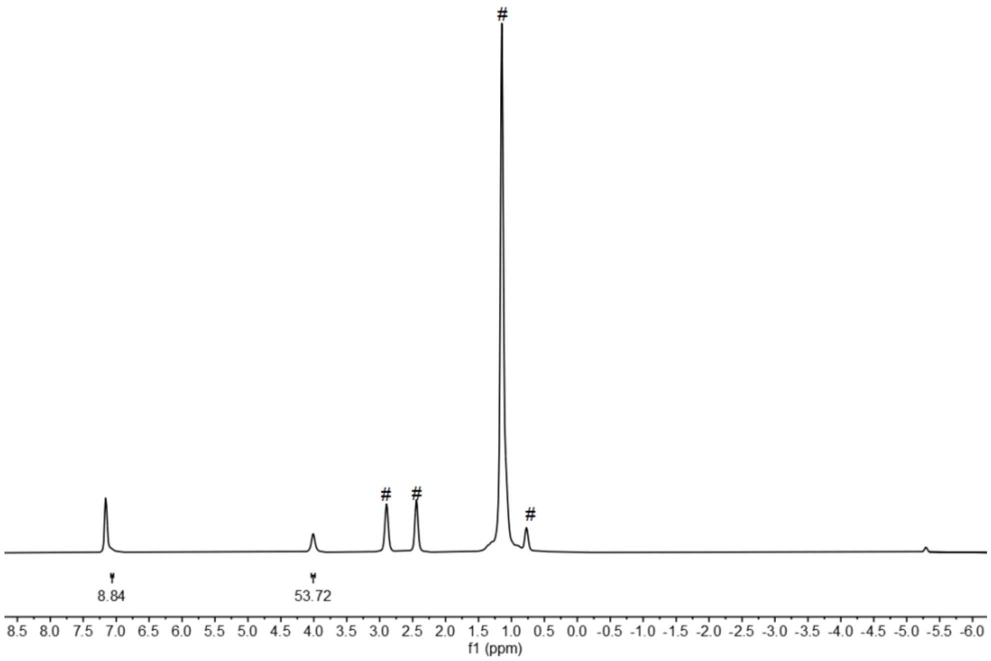

Supplementary Figure 48. Zoom in of the ^{1}H NMR (C_6D_6, 298 K) spectrum of the crude reaction mixture resulting from the reduction of **6U**. Integrated resonances represent two of the four ^{1}H environments of [U(Tren^{TIPS})]: 7.12 (br, 9H, C*H*(CH_3)$_2$), 4.03 (s, 54H, CH(C*H*$_3$)$_2$) ppm. # = Tren^{TIPS}H_3.

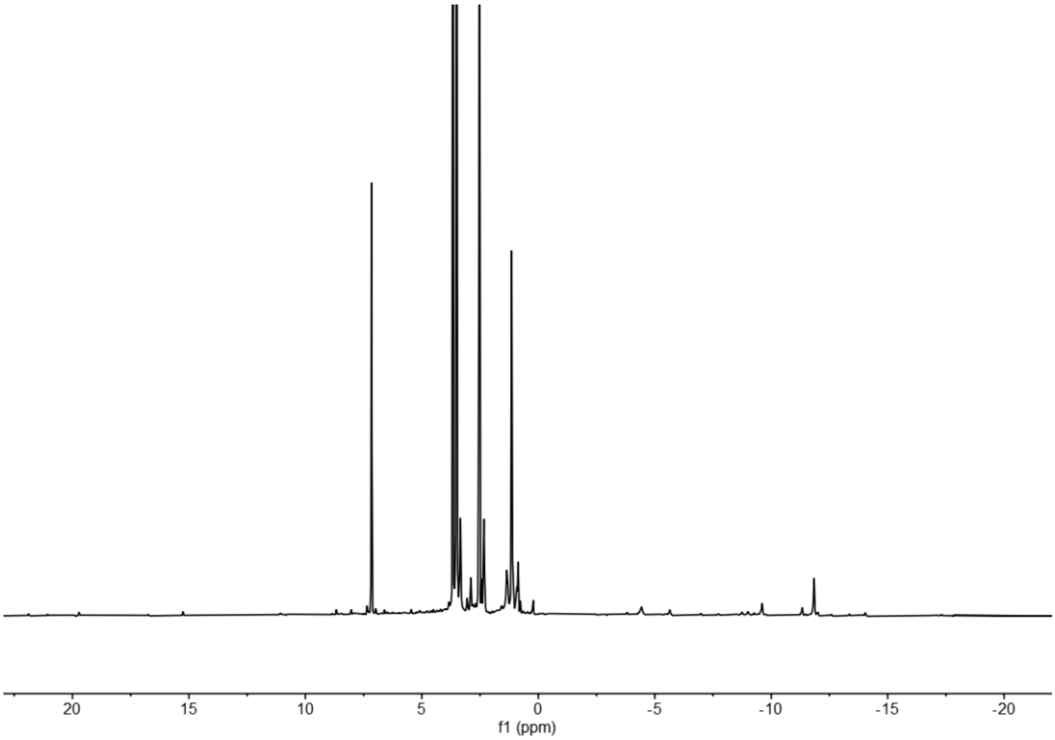

Supplementary Figure 49. ^{1}H NMR (C_6D_6, 298 K) spectrum of the crude reaction mixture resulting from the reduction of **6U** with KC_8 and 2,2,2-cryptand.

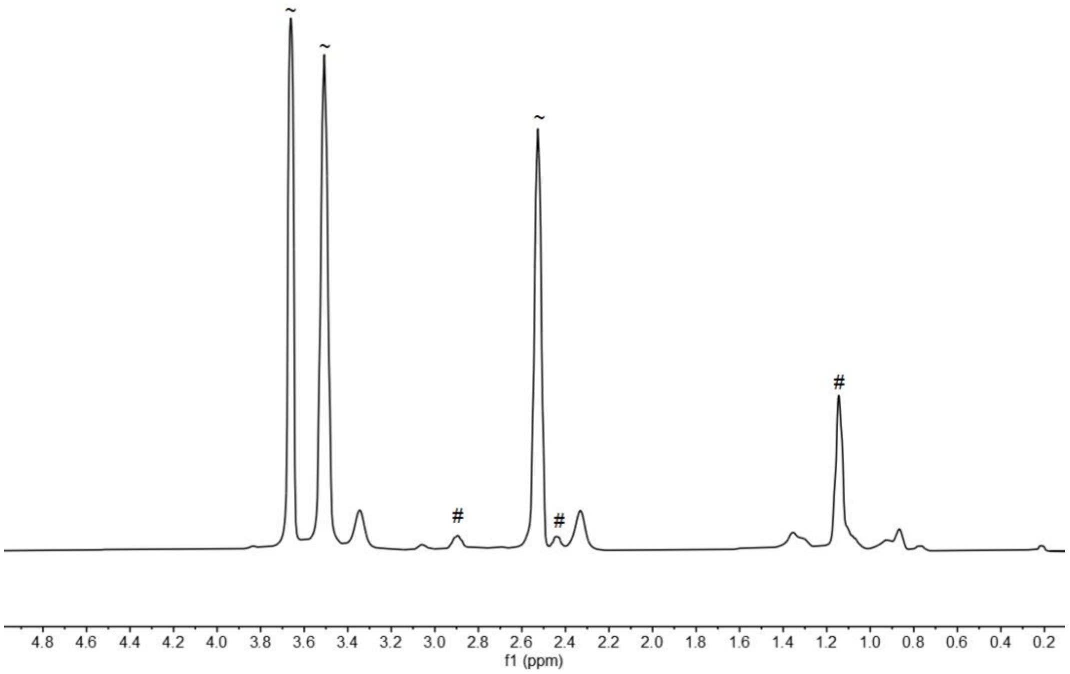

Supplementary Figure 50. Zoom-in of the ^{1}H NMR (C_6D_6, 298 K) spectrum of the crude reaction mixture resulting from the reduction of **6U** with KC_8 and 2,2,2-cryptand. # = $Tren^{TIPS}H_3$, ~ = 2,2,2-cryptand.

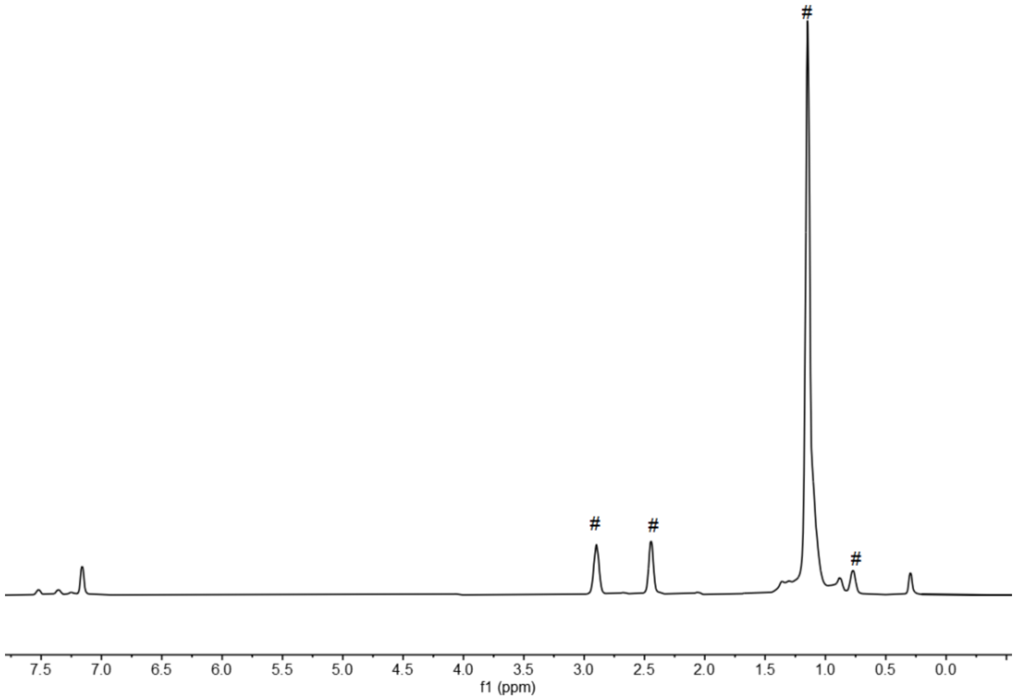

Supplementary Figure 51. ^{1}H NMR (C_6D_6, 298 K) spectrum of the crude reaction mixture resulting from the reduction of **7U:6U** with two equivalents of KC_8. # = $Tren^{TIPS}H_3$.

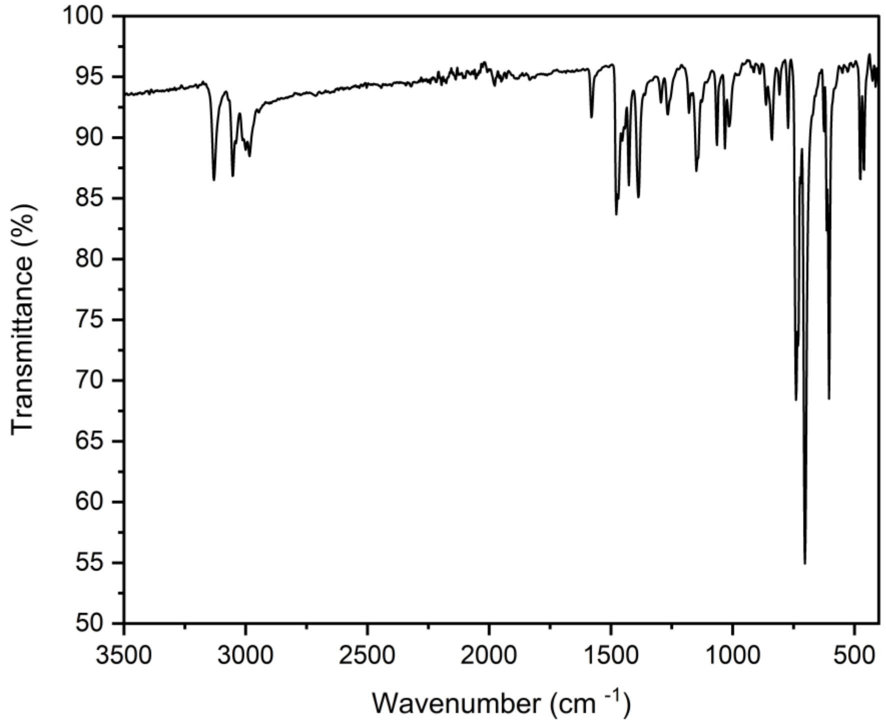

Supplementary Figure 52. ATR-IR spectrum of $[HNEt_3][BPh_4]$.

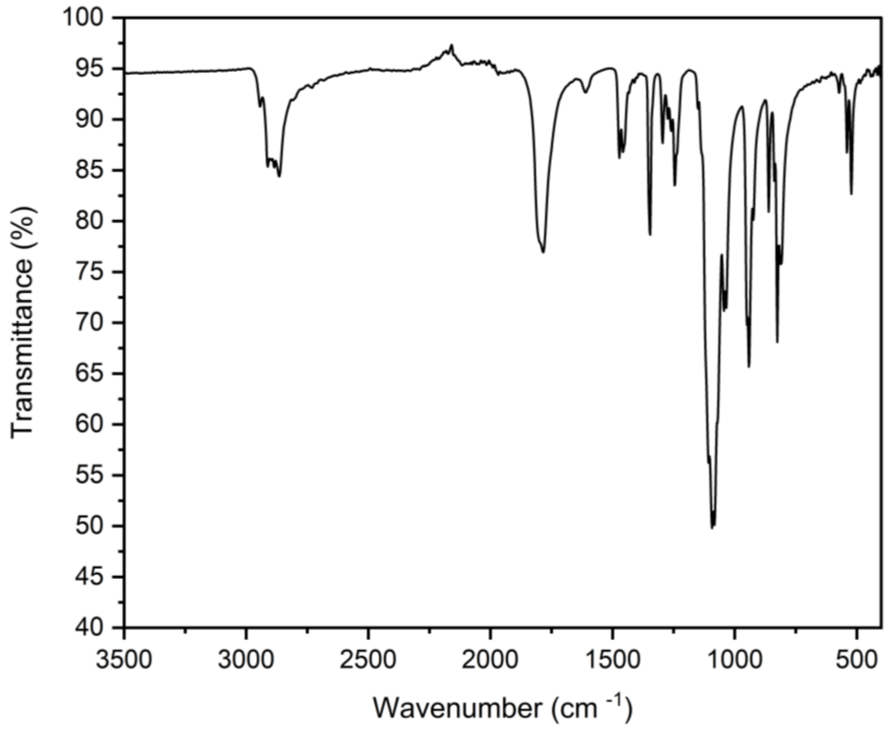

Supplementary Figure 53. ATR-IR spectrum of **1Na**.

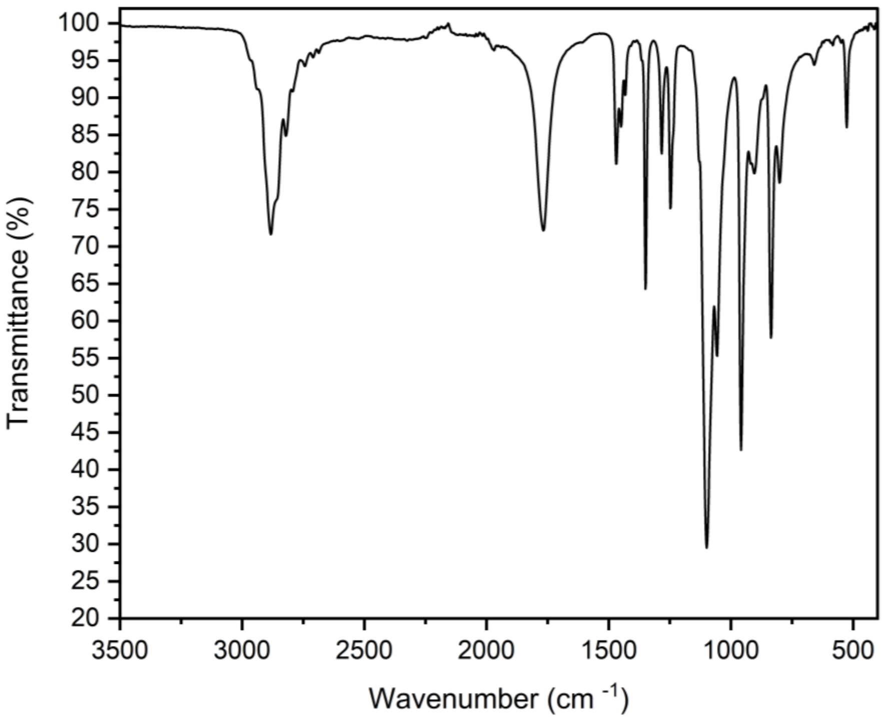

Supplementary Figure 54. ATR-IR spectrum of **1K**.

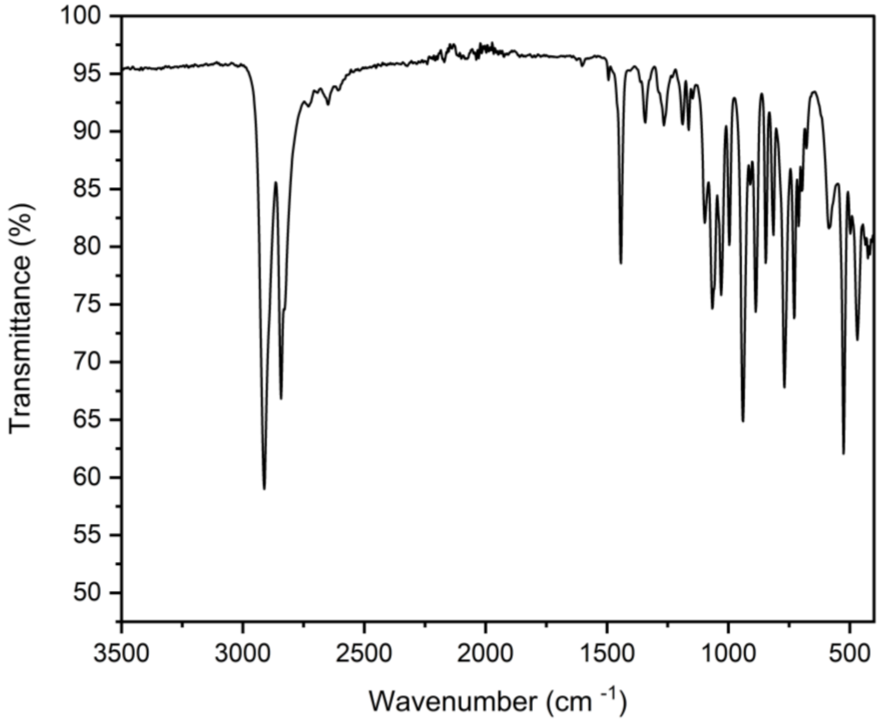

Supplementary Figure 55. ATR-IR spectrum of $Tren^{TCHS}Li_3$.

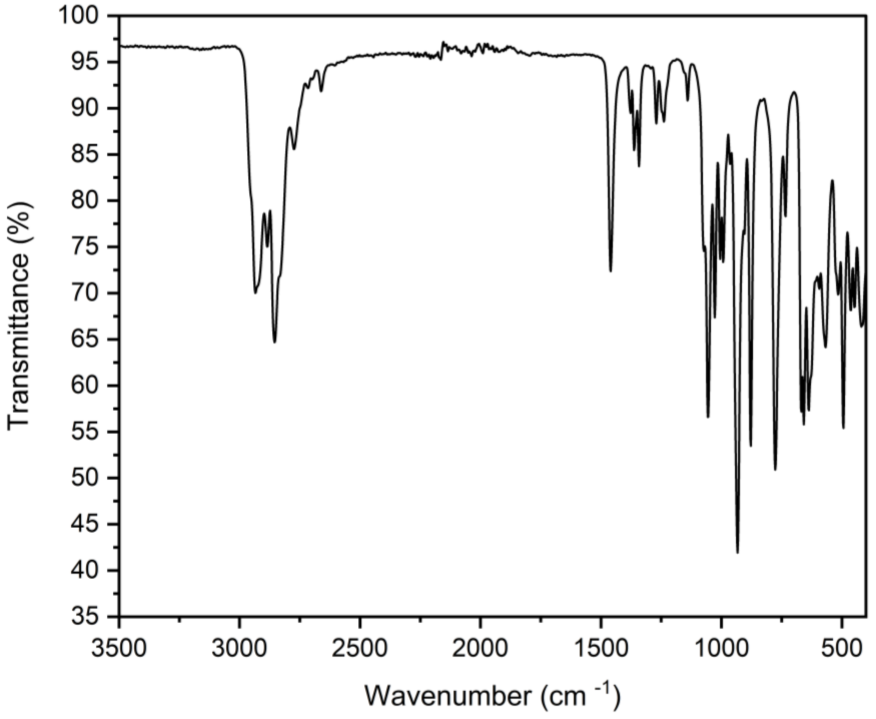

Supplementary Figure 56. ATR-IR spectrum of $Tren^{TIPS}Li_3$.

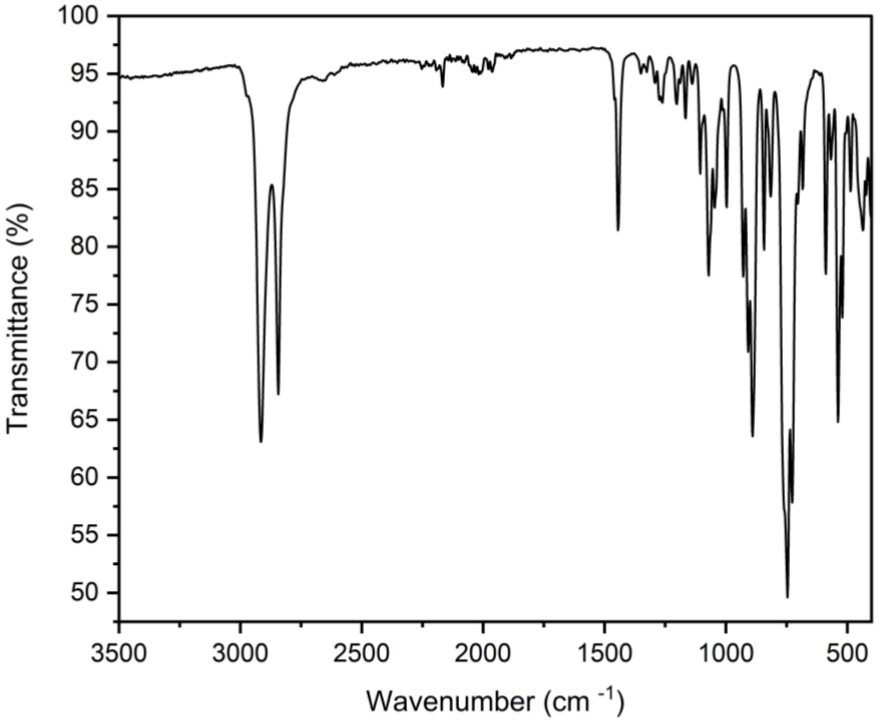

Supplementary Figure 57. ATR-IR spectrum of [U($Tren^{TCHS}$)Cl].

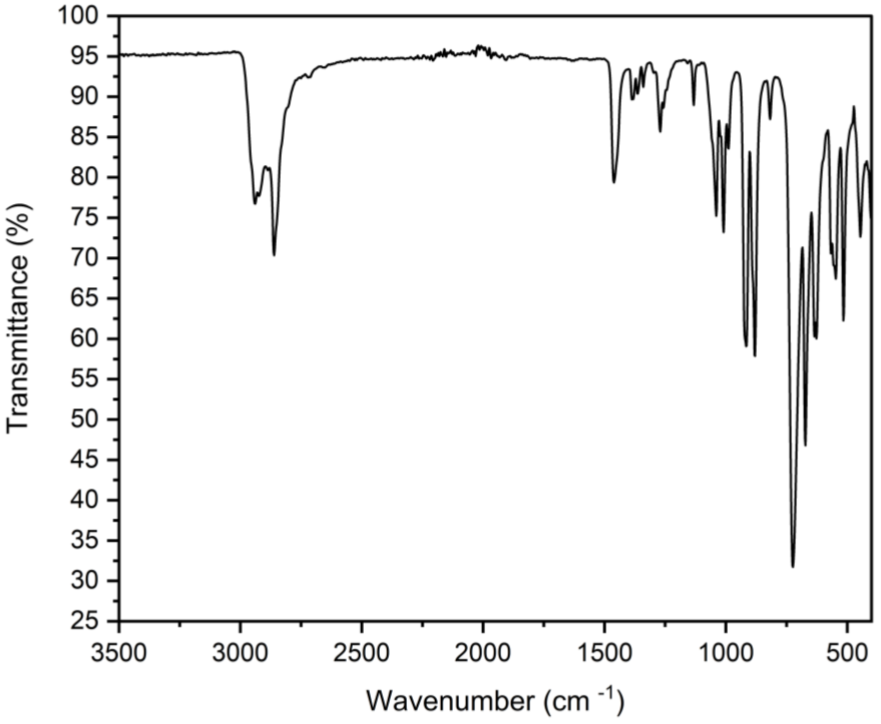

Supplementary Figure 58. ATR-IR spectrum of [U(Tren^{TIPS})Cl].

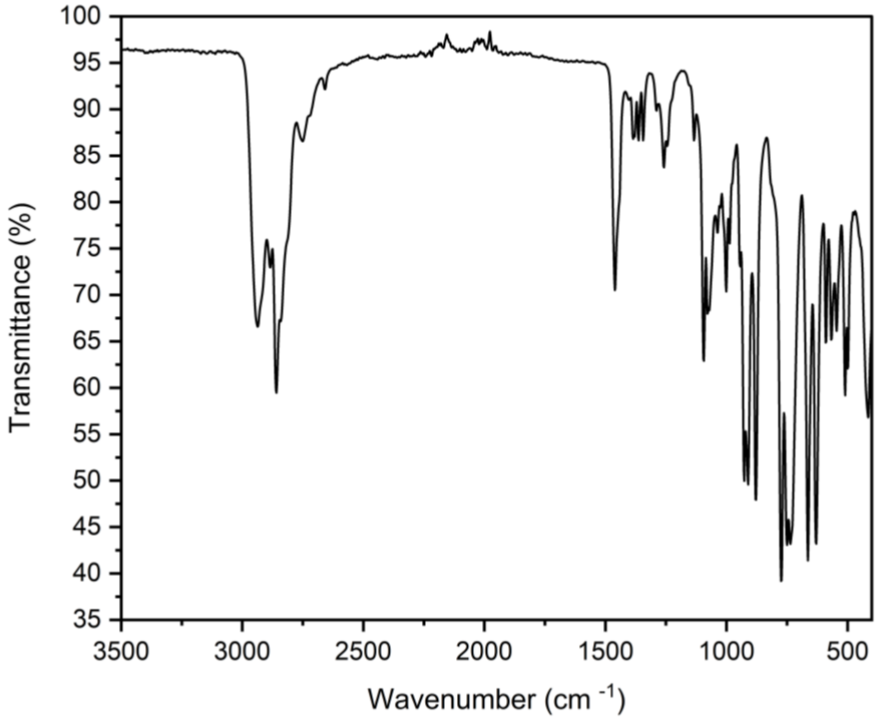

Supplementary Figure 59. ATR-IR spectrum of [U(Tren^{TIPS})].

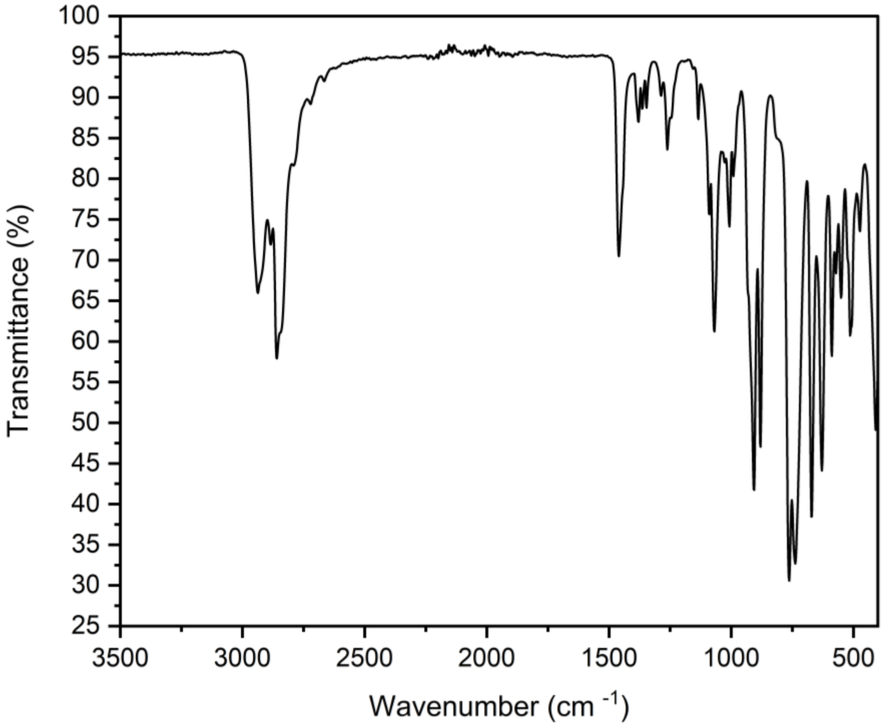

Supplementary Figure 60. ATR-IR spectrum of **2U**.

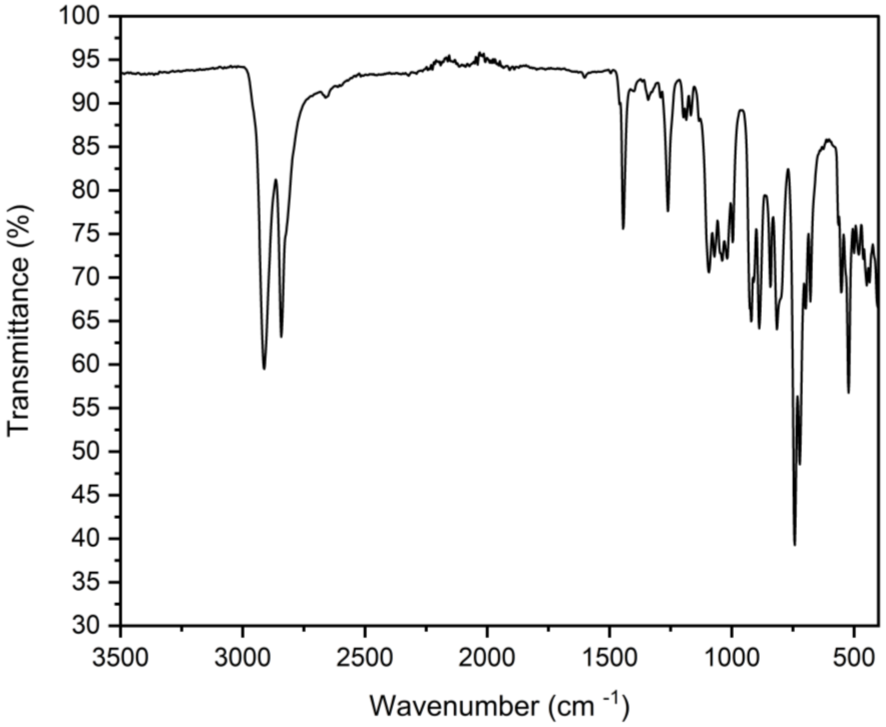

Supplementary Figure 61. ATR-IR spectrum of **3U**.

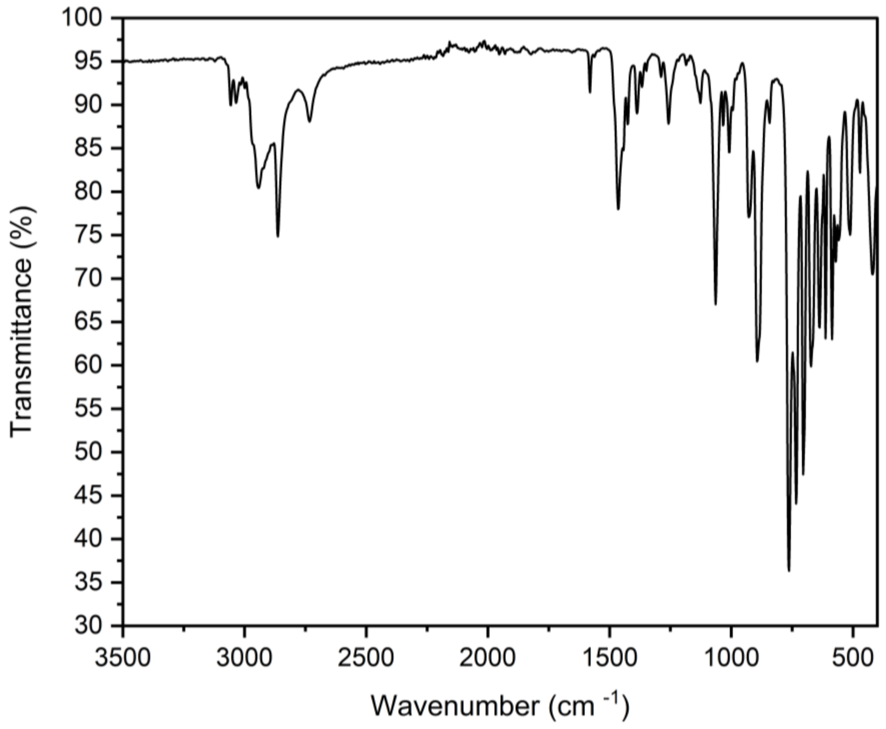

Supplementary Figure 62. ATR-IR spectrum of **4U**.

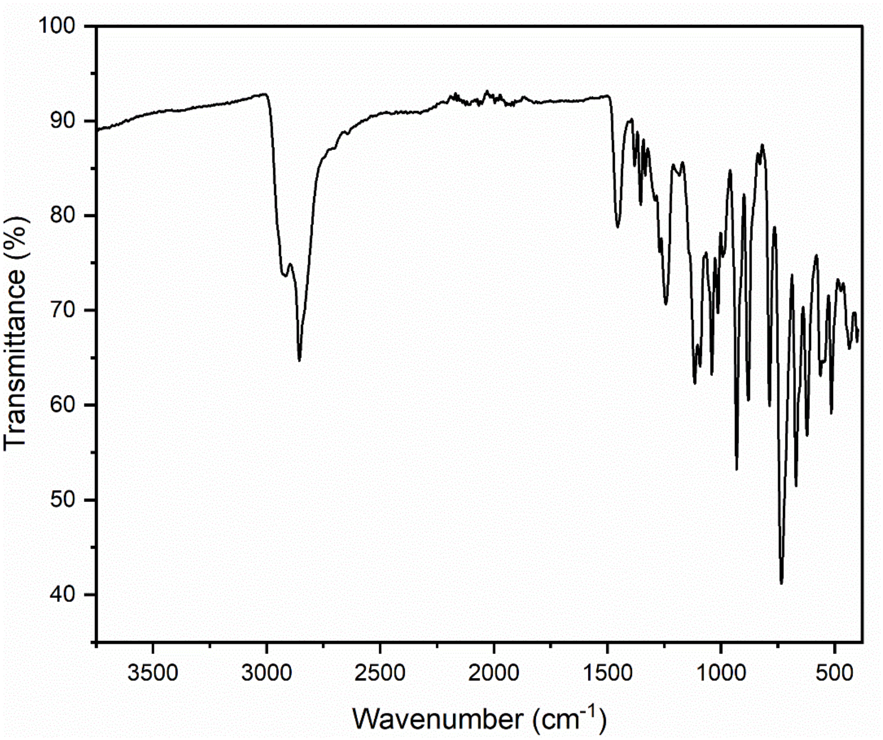

Supplementary Figure 63. ATR-IR spectrum of **5UNa**.

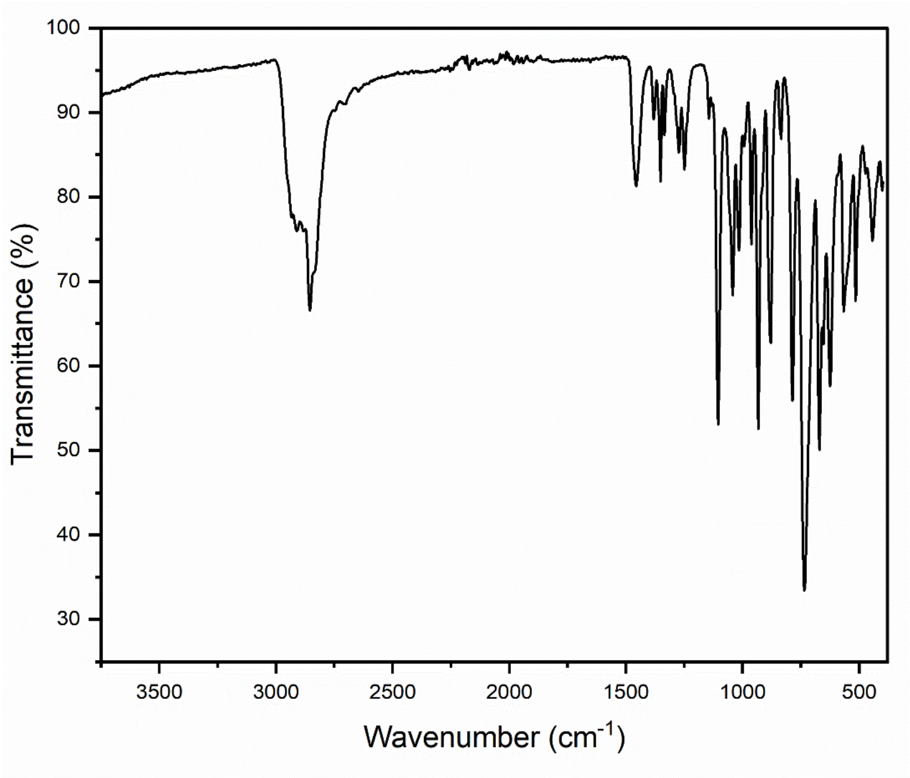

Supplementary Figure 64. ATR-IR spectrum of **5UK**.

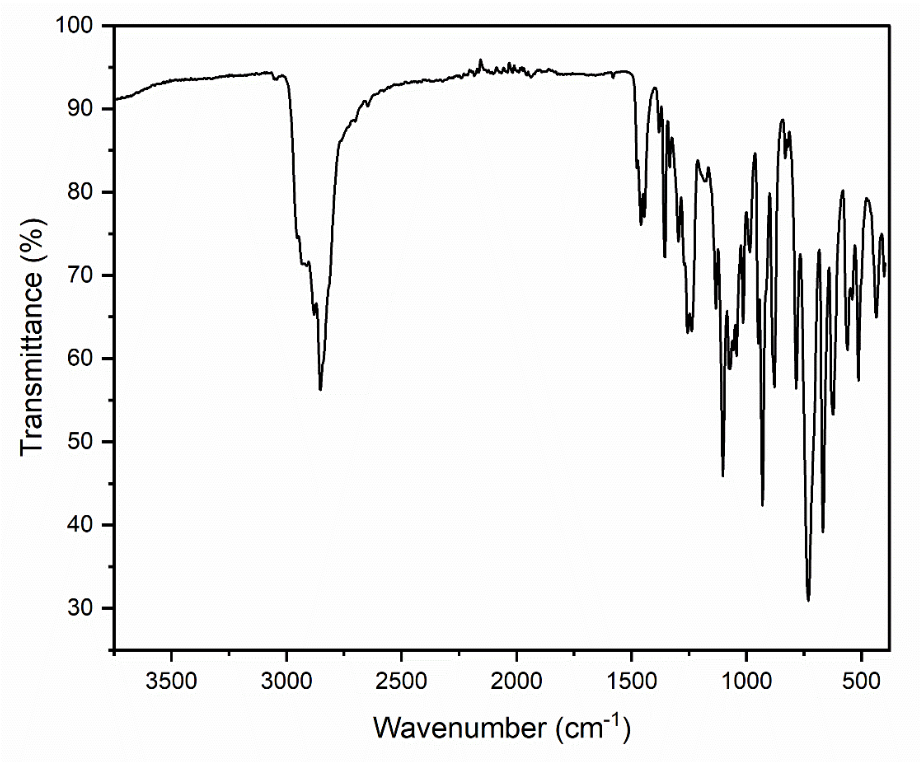

Supplementary Figure 65. ATR-IR spectrum of **5UK'**.

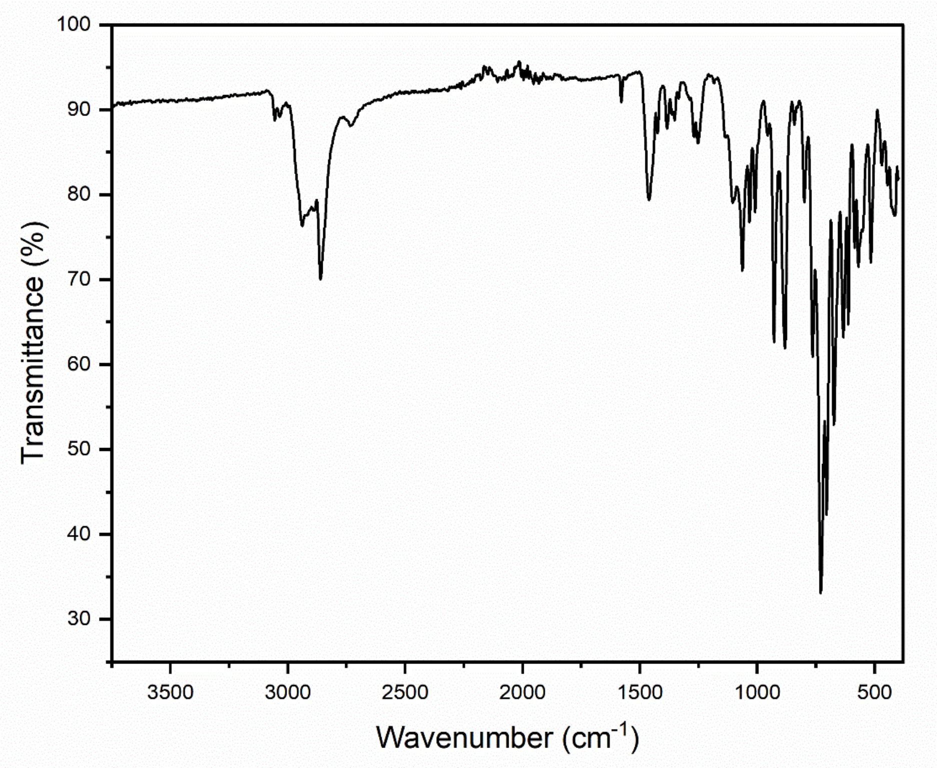

Supplementary Figure 66. ATR-IR spectrum of **6U**.

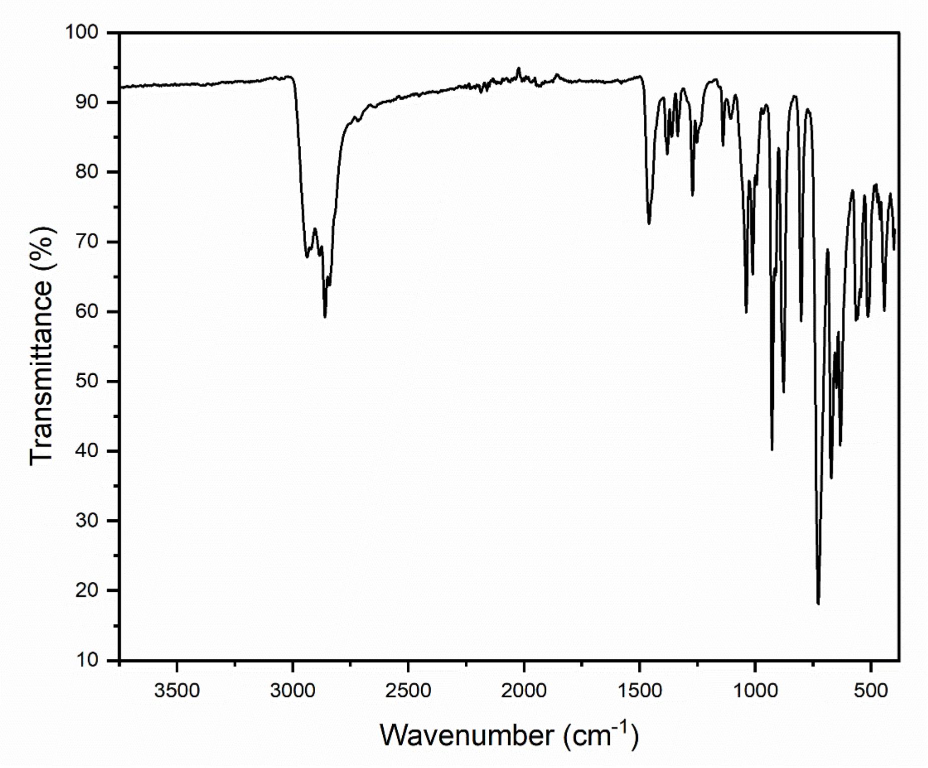

Supplementary Figure 67. ATR-IR spectrum of 11:89 **6U**:**7U**.

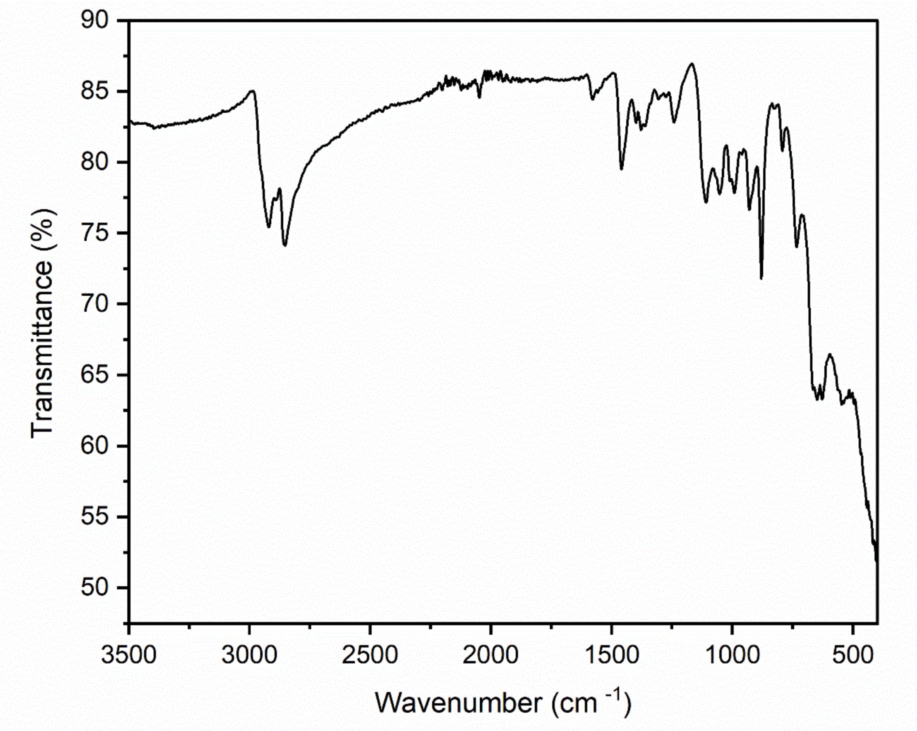

Supplementary Figure 68. ATR-IR spectrum of **8U**.

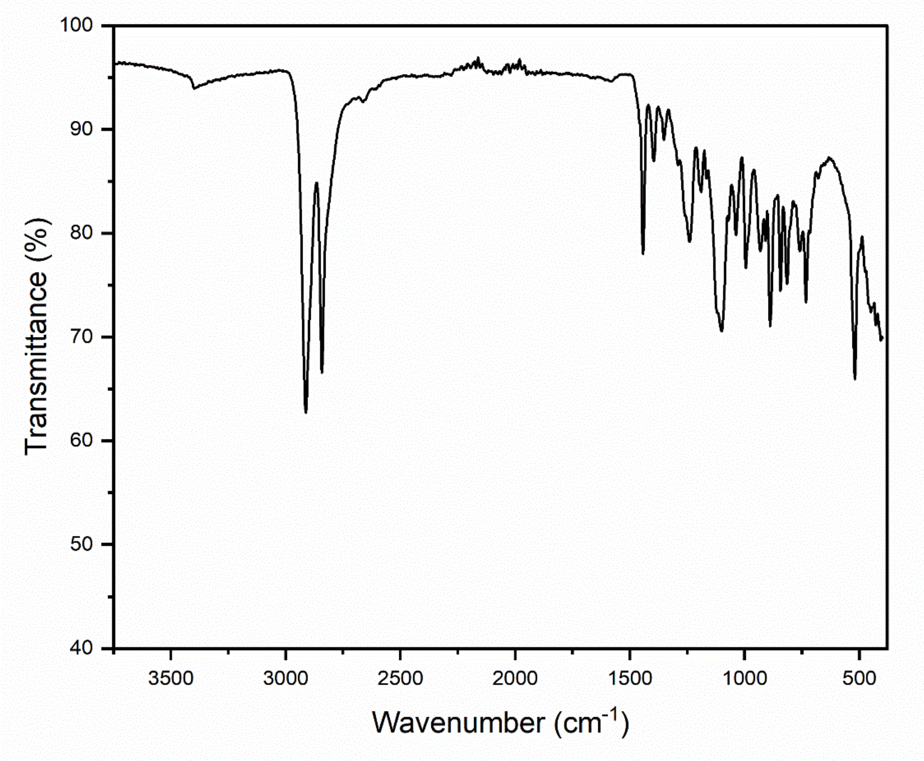

Supplementary Figure 69. ATR-IR spectrum of **9UNa**.

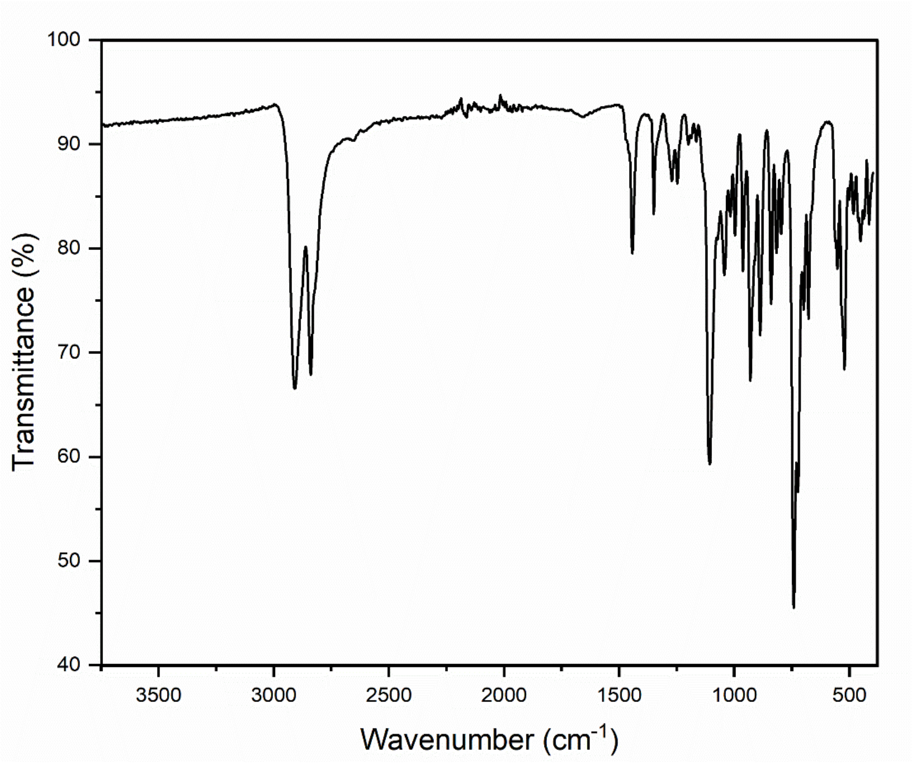

Supplementary Figure 70. ATR-IR spectrum of **9UK**.

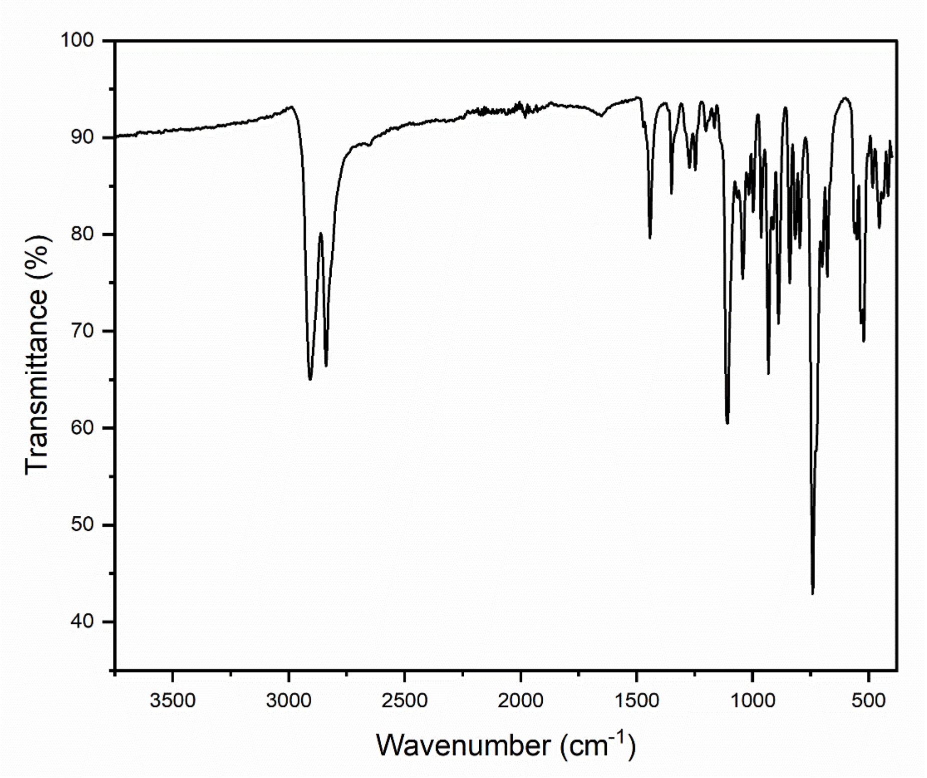

Supplementary Figure 71. ATR-IR spectrum of **10UK**.

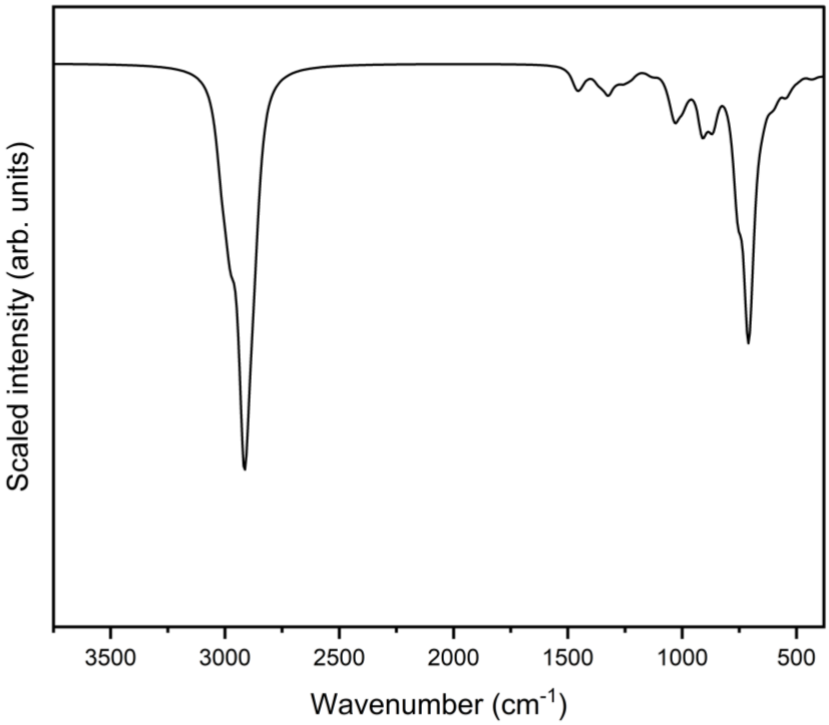

Supplementary Figure 72. Computed IR spectrum of **5U**.

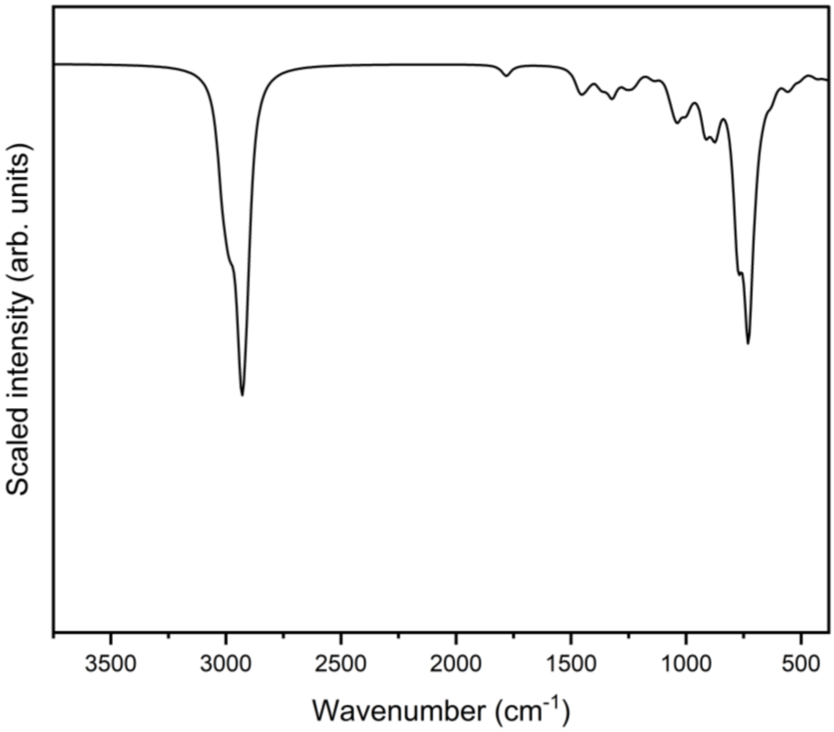

Supplementary Figure 73. Computed IR spectrum of **6U**.

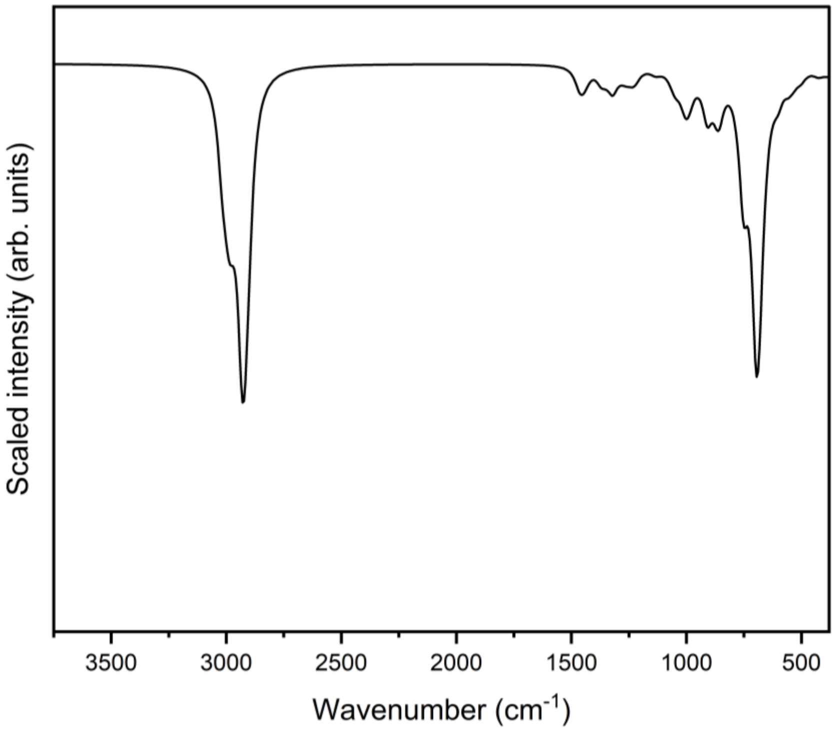

Supplementary Figure 74. Computed IR spectrum of **7U**.

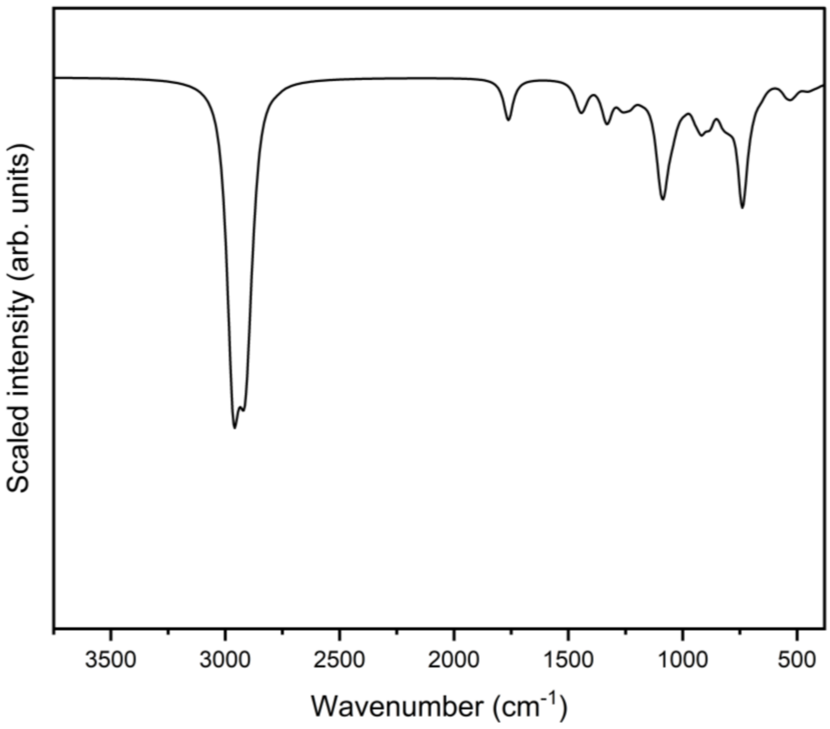

Supplementary Figure 75. Computed IR spectrum of **9UK**.

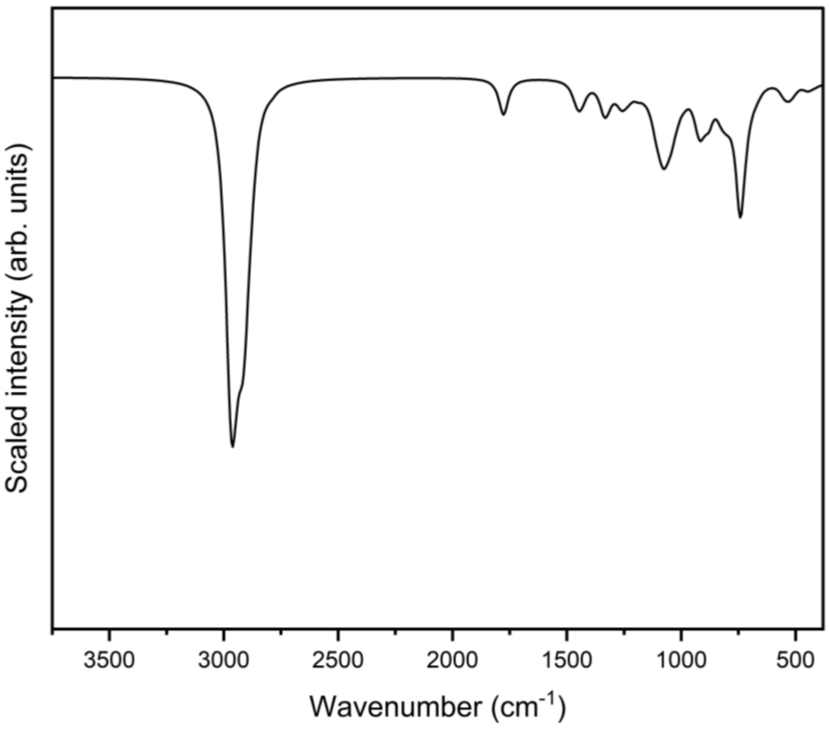

Supplementary Figure 76. Computed IR spectrum of **9UNa**.

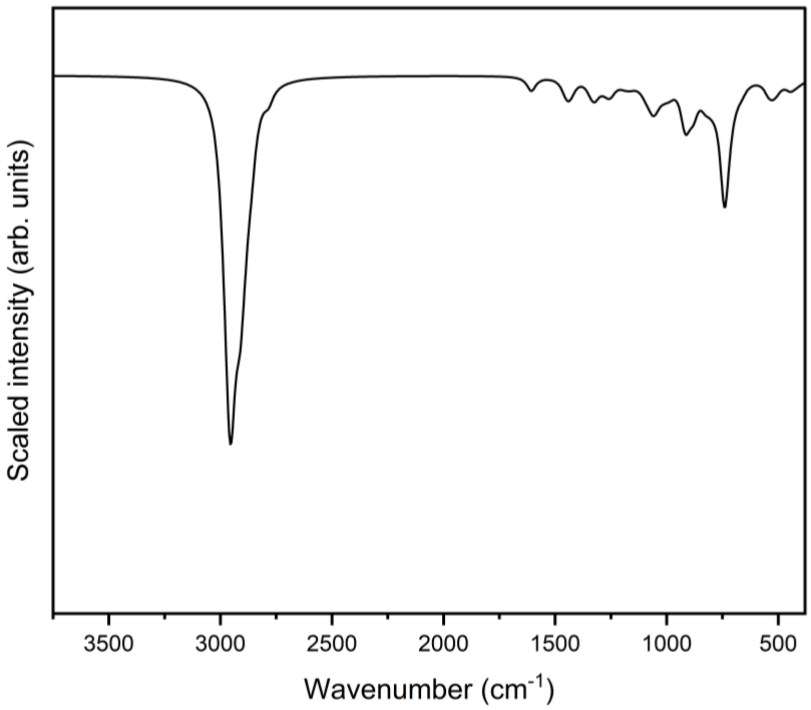

Supplementary Figure 77. Computed IR spectrum of **10UK**.

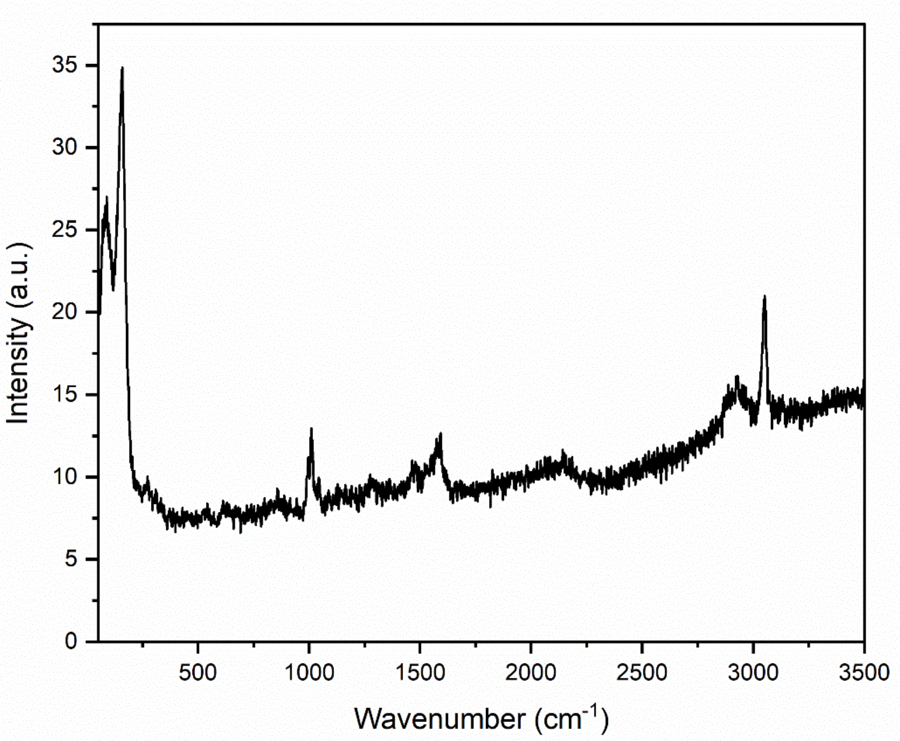

Supplementary Figure 78. Raman spectrum of crystalline 11:89 **6U**:**7U** recorded over the range of 50 to 3500 cm^{-1}.

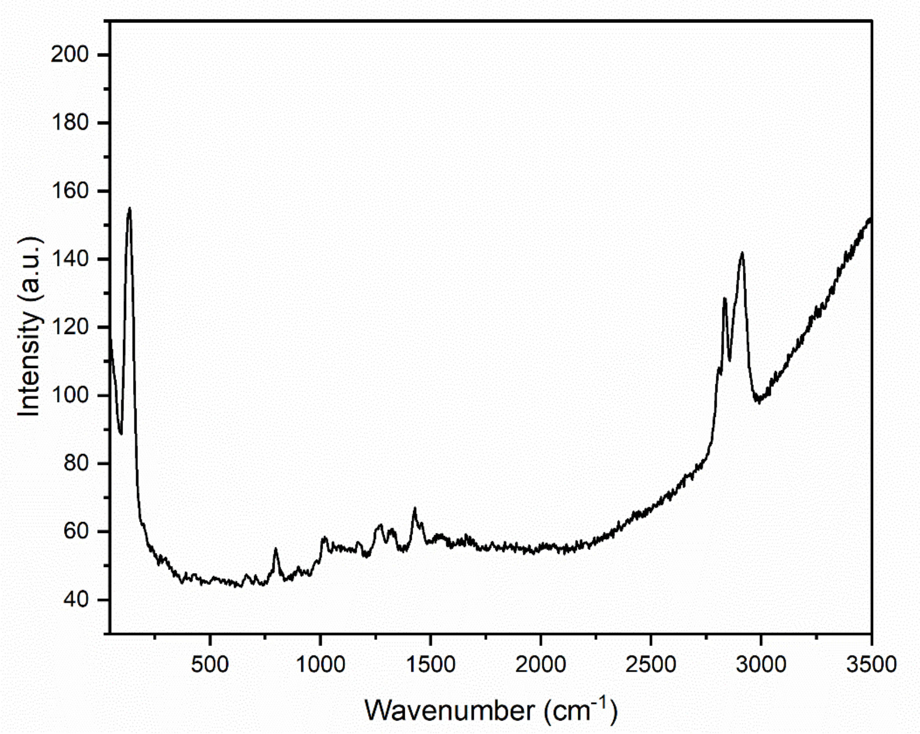

Supplementary Figure 79. Raman spectrum of crystalline **9UK** recorded over the range of 50 to 3500 cm^{-1}.

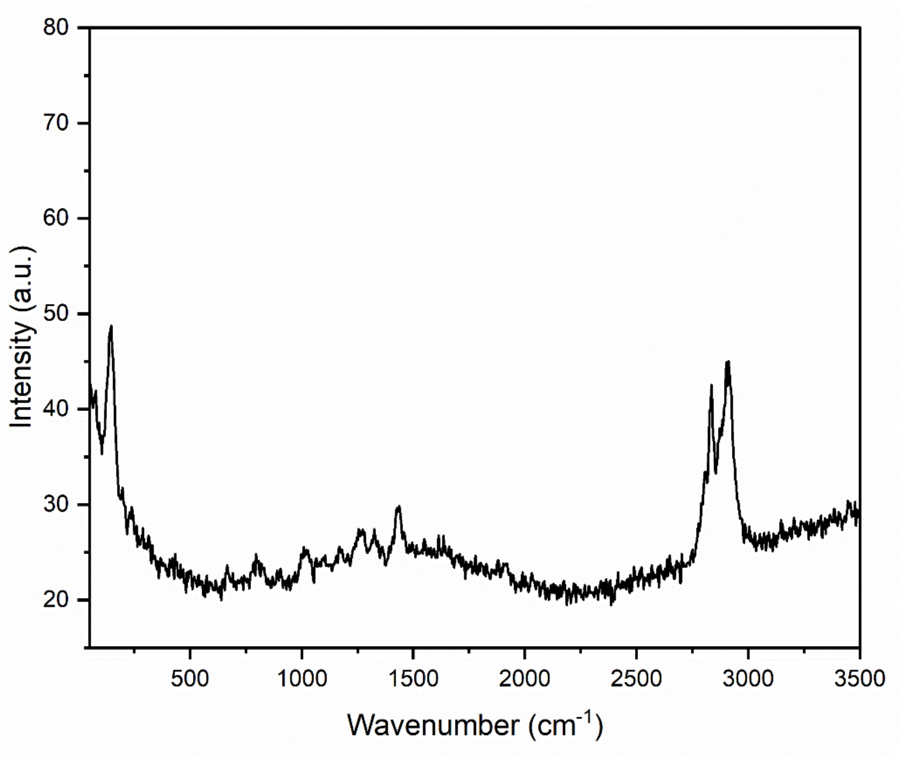

Supplementary Figure 80. Raman spectrum of crystalline **10UK** recorded over the range of 50 to 3500 cm^{-1}.

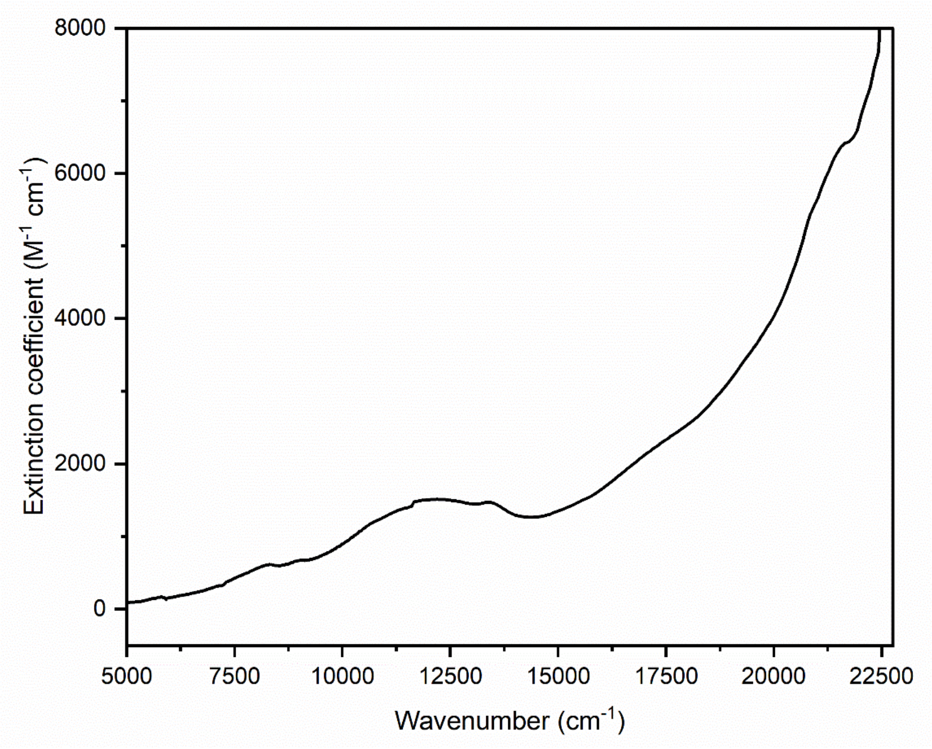

Supplementary Figure 81. UV/Vis/NIR spectra of **5UNa** in THF (5 mM) over the range 5000-22500 cm^{-1}.

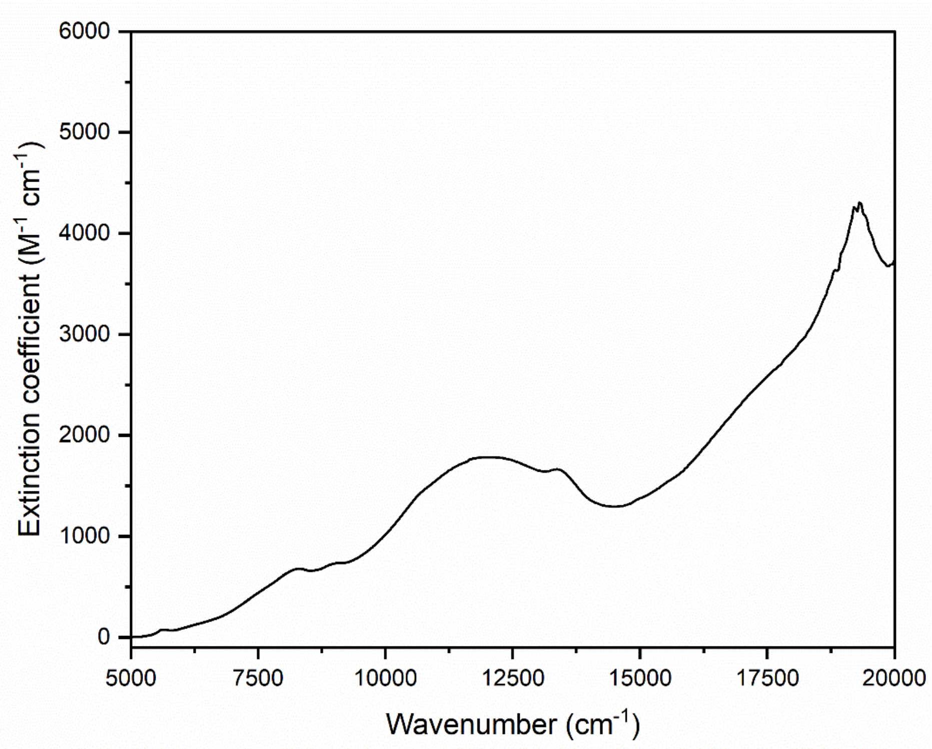

Supplementary Figure 82. UV/Vis/NIR spectra of **5UK** in THF (10 mM) over the range 5000-20000 cm^{-1}.

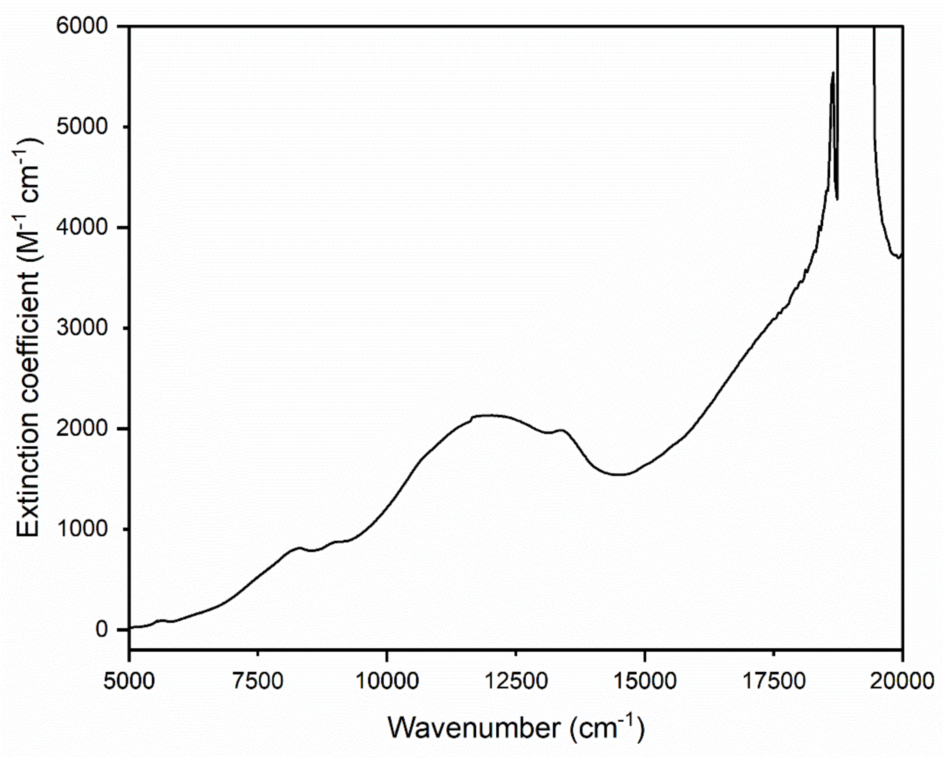

Supplementary Figure 83. UV/Vis/NIR spectra of **5UK'** in THF (5 mM) over the range 5000-20000 cm^{-1}.

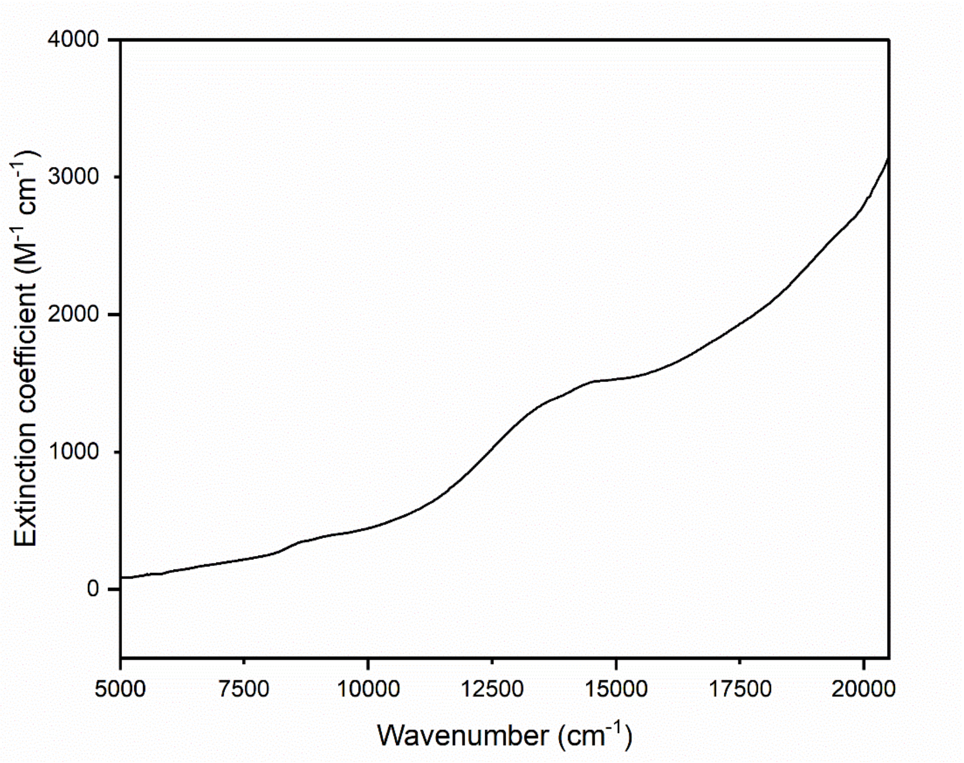

Supplementary Figure 84. UV/Vis/NIR spectra of **6U**:**7U** in THF (10 mM) over the range 5000-20000 cm^{-1}.

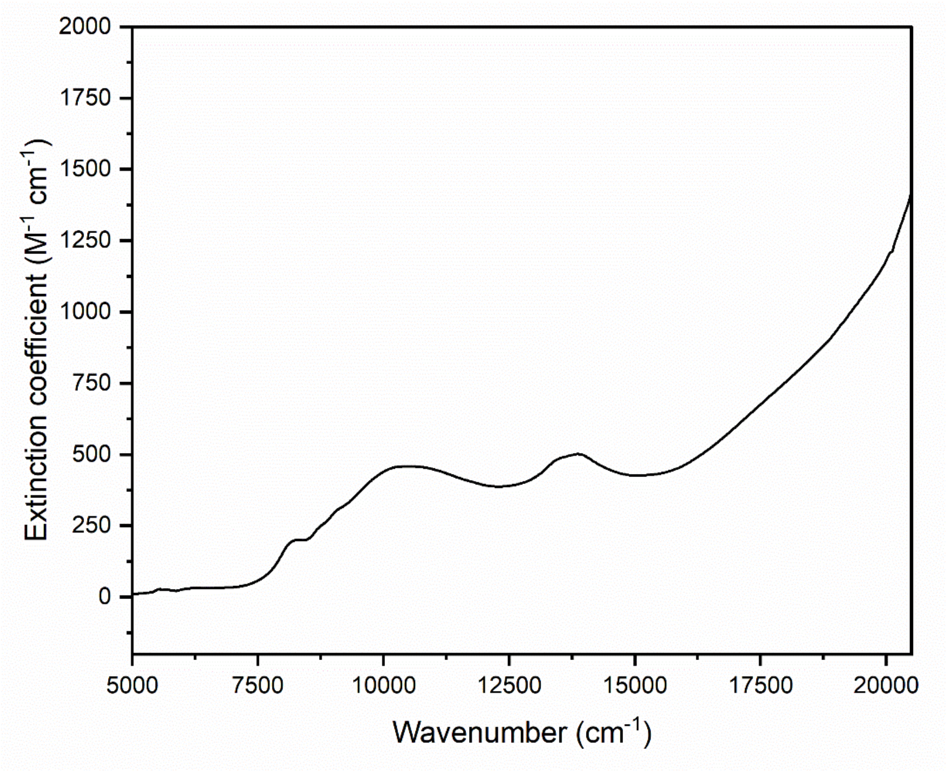

Supplementary Figure 85. UV/Vis/NIR spectra of **10UK** in THF (25 mM) over the range 5000-20000 cm^{-1}.

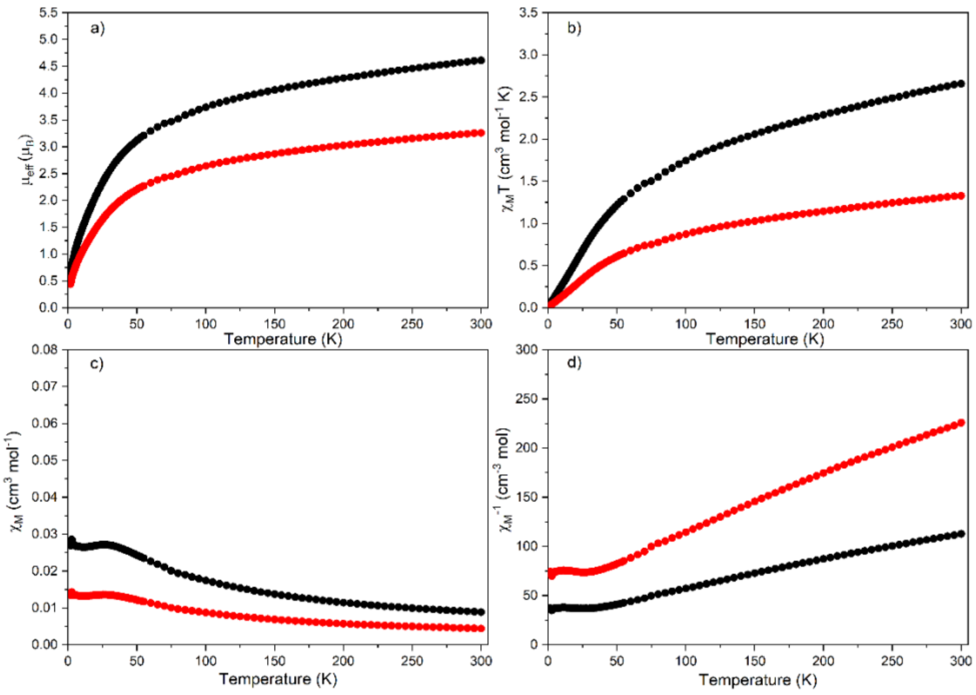

Supplementary Figure 86. Variable-temperature SQUID magnetometry of **5UNa** (per molecule: black; per U: red) over the temperature range 300-1.8 K: a) μ_{eff} vs T; b) χ_MT vs T; c) χ_M vs T; d) χ_M^{-1} vs T. The lines are a guide to the eye only.

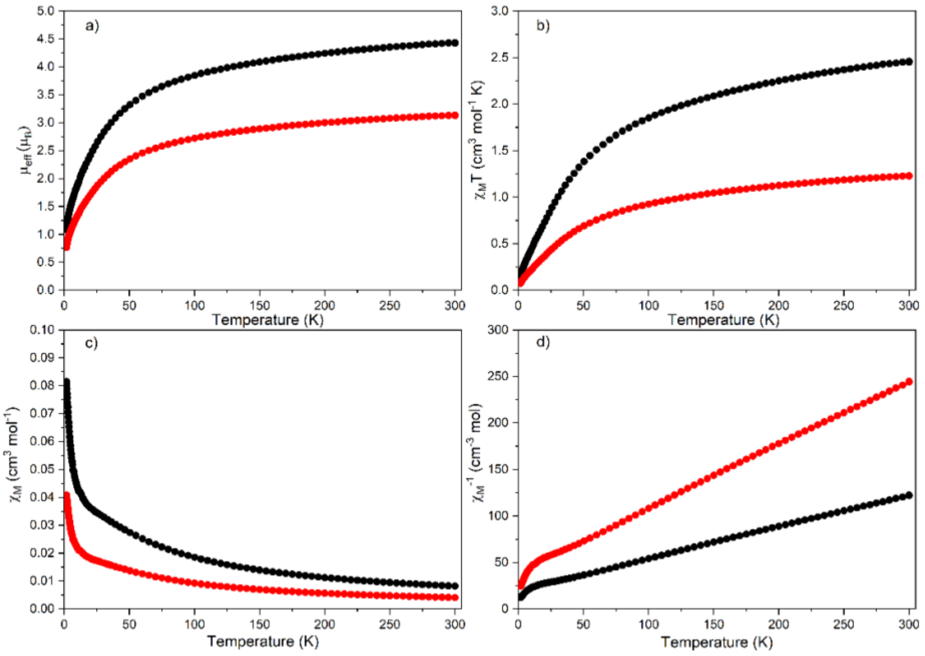

Supplementary Figure 87. Variable-temperature SQUID magnetometry of **5UK** (per molecule: black; per U: red) over the temperature range 300-1.8K: a) μ_{eff} vs T; b) χ_MT vs T; c) χ_M vs T; d) χ_M^{-1} vs T. The lines are a guide to the eye only.

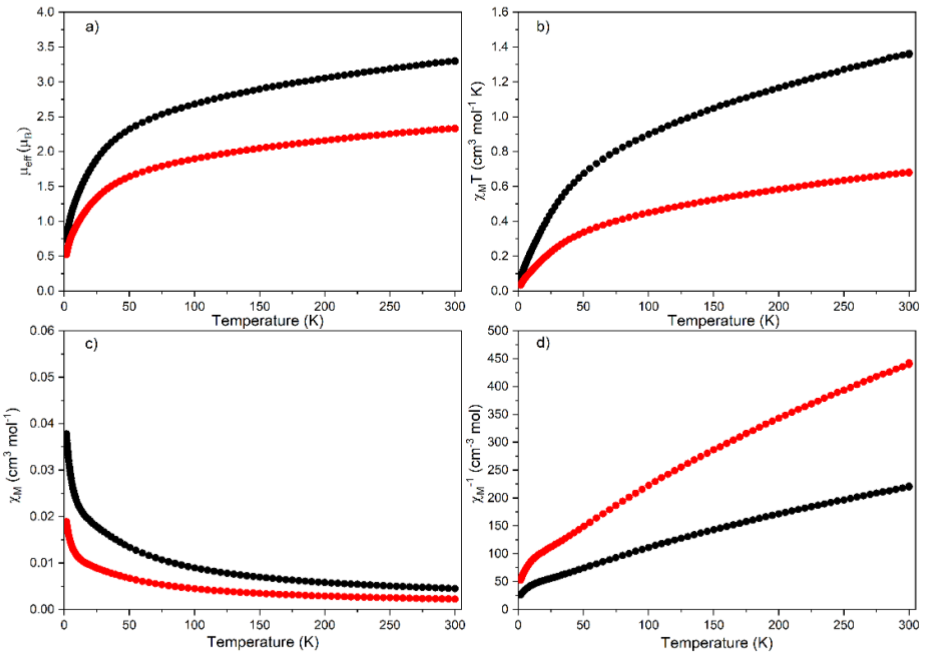

Supplementary Figure 88. Variable-temperature SQUID magnetometry of **5UK'** (per molecule: black; per U: red) over the temperature range 300-1.8 K: a) μ_{eff} vs T; b) $\chi_M T$ vs T; c) χ_M vs T; d) χ_M^{-1} vs T. The lines are a guide to the eye only.

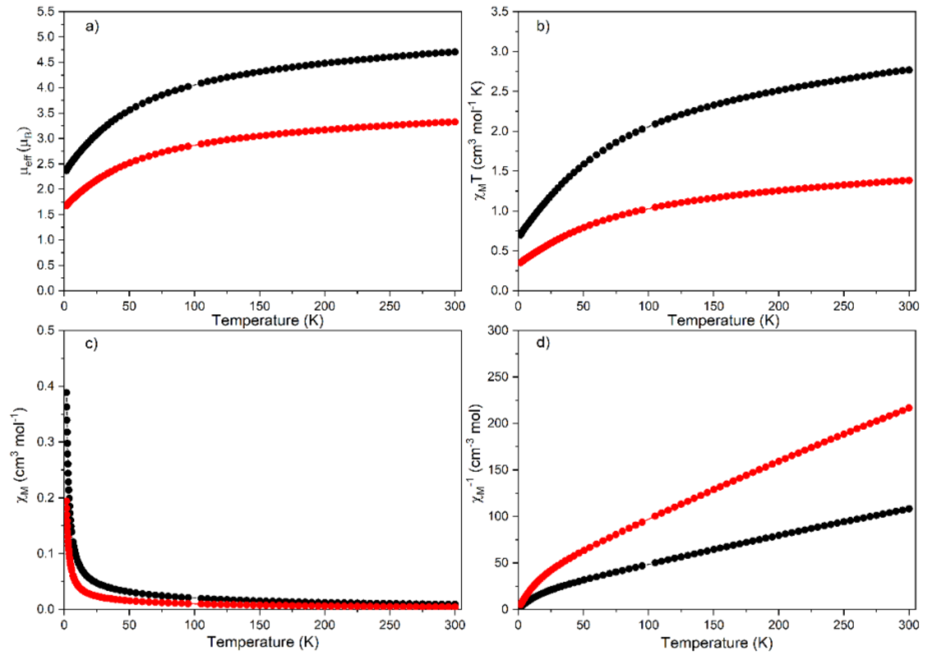

Supplementary Figure 89. Variable-temperature SQUID magnetometry of **6U** (per molecule: black; per U: red) over the temperature range 300-1.8 K: a) μ_{eff} vs T; b) $\chi_M T$ vs T; c) χ_M vs T; d) χ_M^{-1} vs T. The lines are a guide to the eye only.

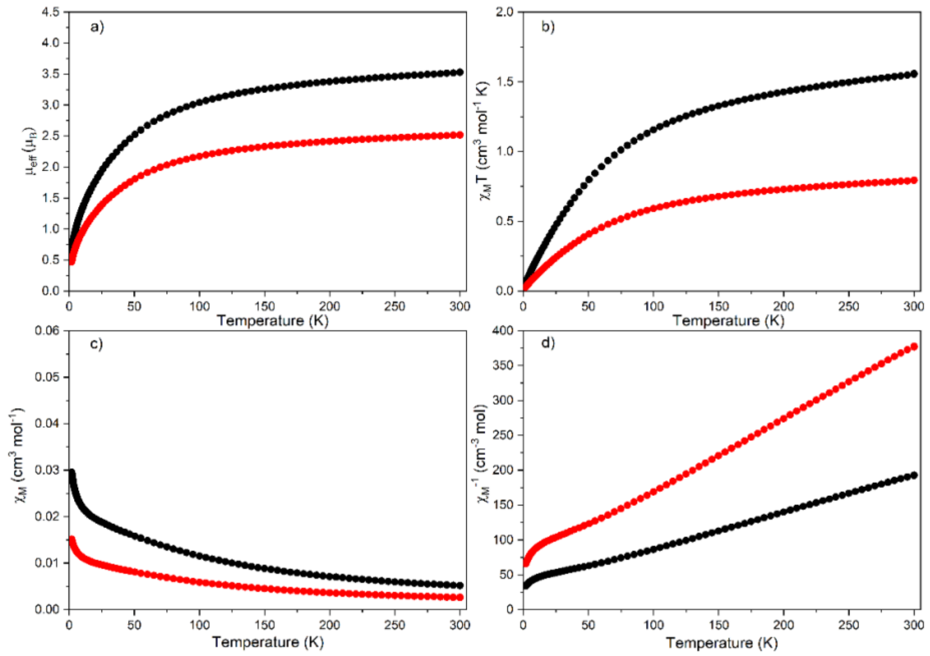

Supplementary Figure 90. Variable-temperature SQUID magnetometry of 11:89 **6U**:**7U** (per molecule: black; per U: red) over the temperature range 300-1.8 K: a) μ_{eff} vs T; b) $\chi_M T$ vs T; c) χ_M vs T; d) χ_M^{-1} vs T. The lines are a guide to the eye only.

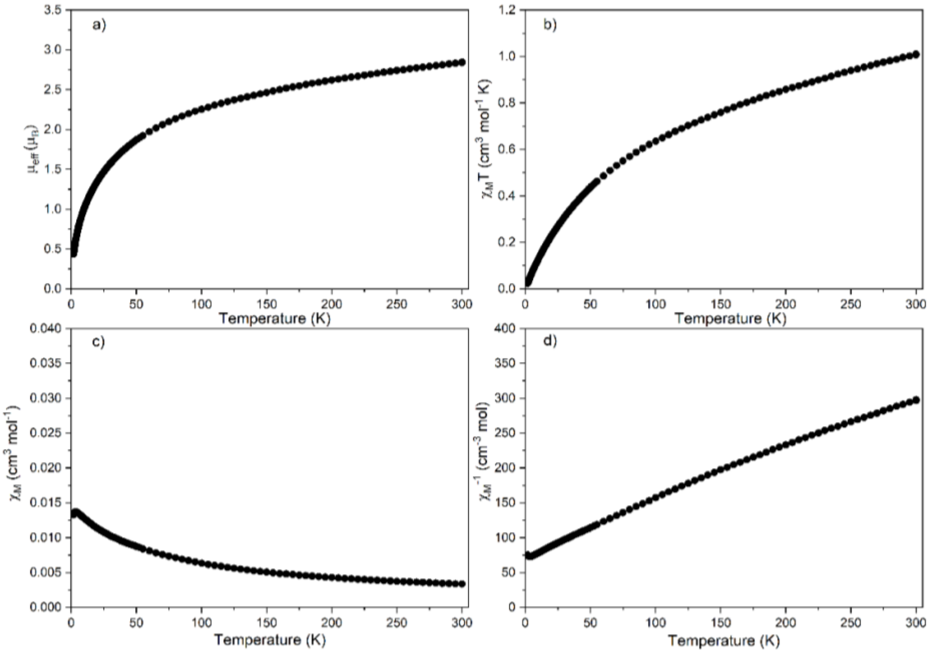

Supplementary Figure 91. Variable-temperature SQUID magnetometry of **9UNa** over the temperature range 300-1.8 K: a) μ_{eff} vs T; b) $\chi_M T$ vs T; c) χ_M vs T; d) χ_M^{-1} vs T. The lines are a guide to the eye only.

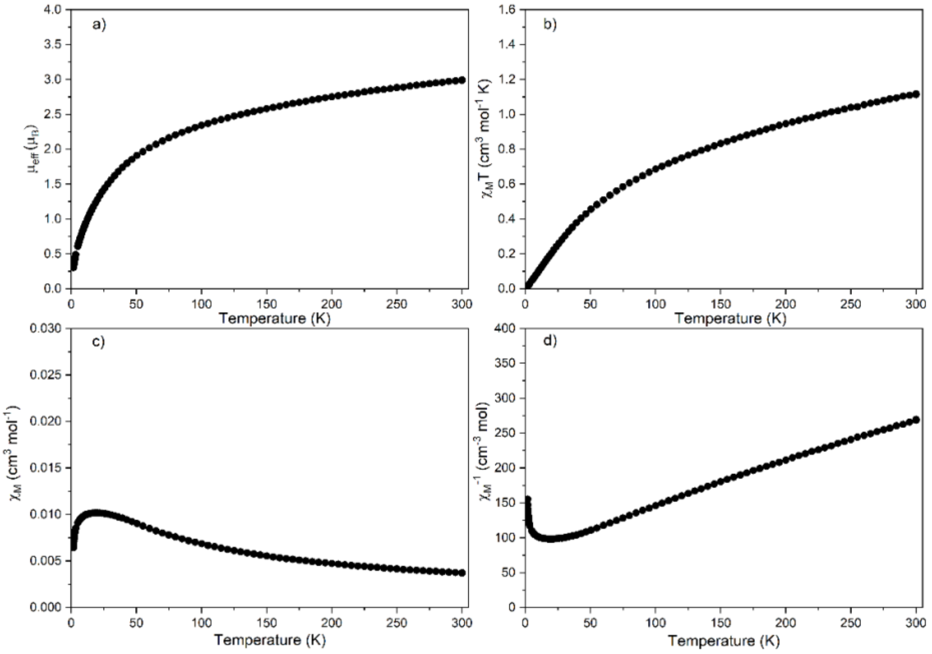

Supplementary Figure 92. Variable-temperature SQUID magnetometry of **9UK** over the temperature range 300-1.8 K: a) μ_{eff} vs T; b) $\chi_M T$ vs T; c) χ_M vs T; d) χ_M^{-1} vs T. The lines are a guide to the eye only.

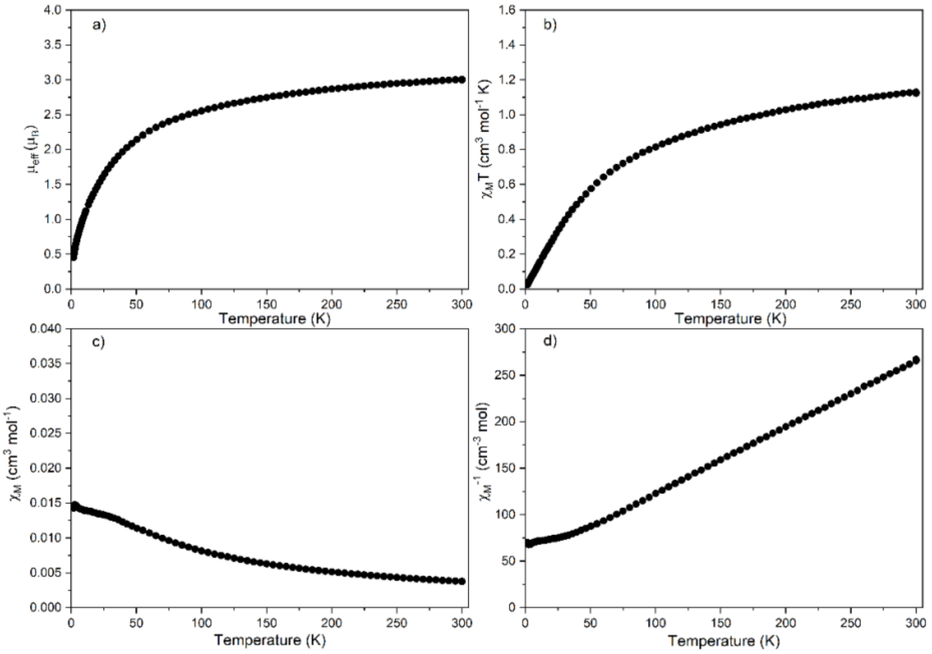

Supplementary Figure 93. Variable-temperature SQUID magnetometry of **10UK** over the temperature range 300-1.8 K: a) μ_{eff} vs T; b) $\chi_M T$ vs T; c) χ_M vs T; d) χ_M^{-1} vs T. The lines are a guide to the eye only.

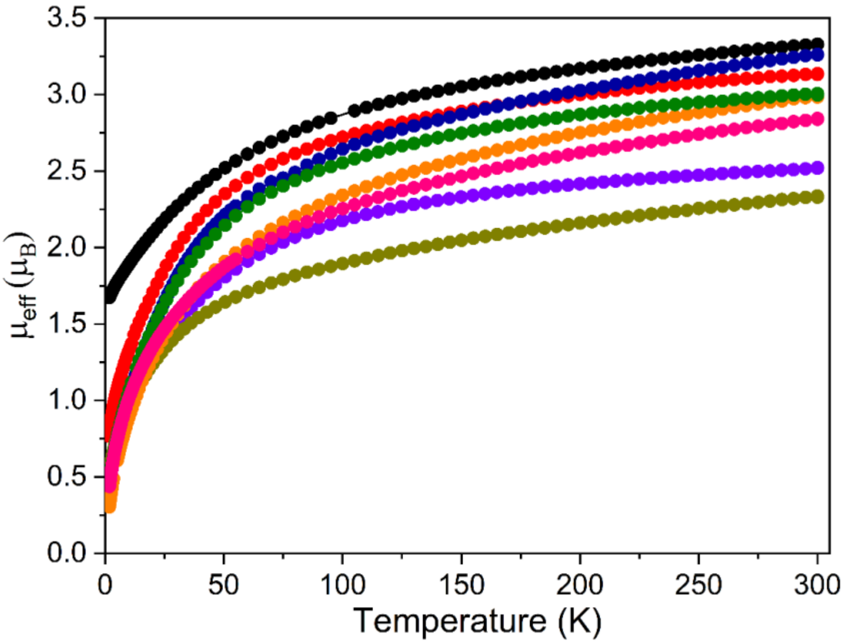

Supplementary Figure 94. Comparative variable-temperature SQUID magnetometry of **5UNa** (dark blue), **5UK** (red), **5UK'** (purple), **6U** (black), **6U**:**7U** (dark yellow), **9UNa** (pink), **9UK** (orange), and **10UK** (green), at 0.1 or 0.5 T plotting μ_{eff} (μ_B) vs Temperature (K) per ion over the temperature range 300 to 1.8 K. The line is a guide to the eye only.

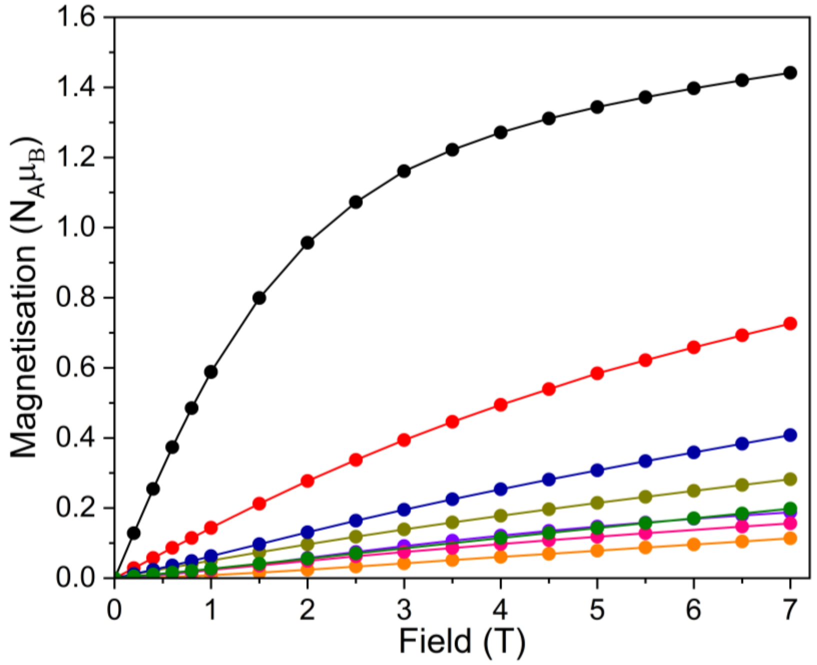

Supplementary Figure 95. Comparative Isothermal Magnetization ($N_A\mu_B$) vs Field (Tesla) data per molecule for **5UNa** (dark blue), **5UK** (red), **5UK'** (purple), **6U** (black), **6U**:**7U** (dark yellow), **9UNa** (pink), **9UK** (orange), and **10UK** (green), at 1.8 K from 0-7 Tesla.

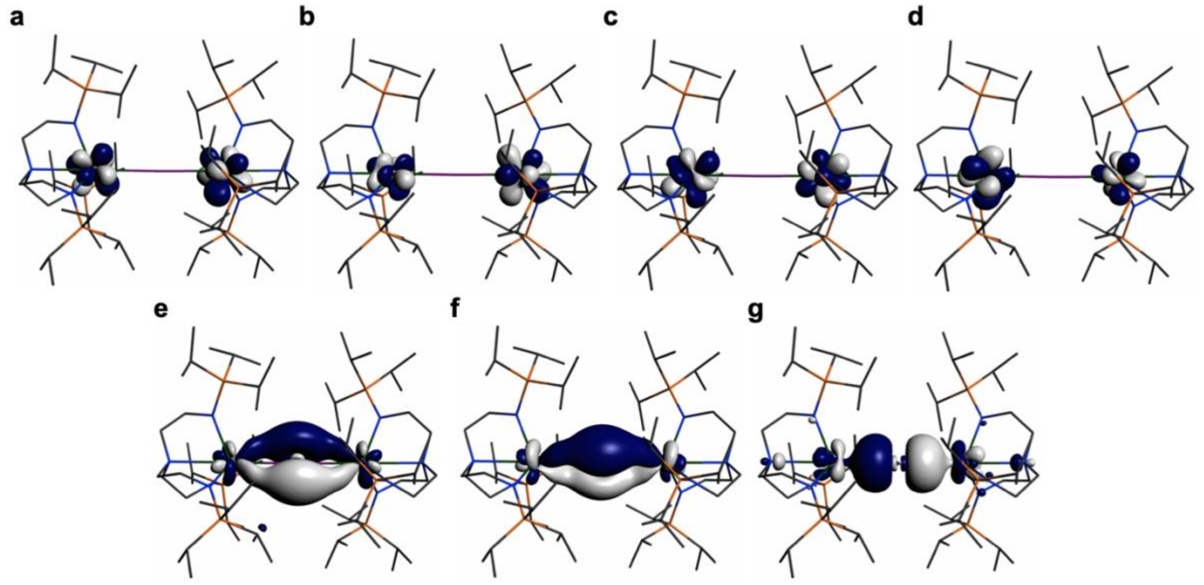

Supplementary Figure 96. Frontier α-spin Kohn Sham Molecular Orbitals of **5U**. a) HOMO (463a, –0.304 eV), b) HOMO–1 (462a, –0.316 eV), c) HOMO–2 (461a, –0.325 eV), d) HOMO–3 (460a, –0.344 eV), e) HOMO–4 (459, –0.960 eV), f) HOMO–5 (458, – 0.976 eV), g) HOMO–6 (457, −1.623 eV). H-atoms are omitted for clarity. Plotted at the 0.04 a.u. isosurface level.

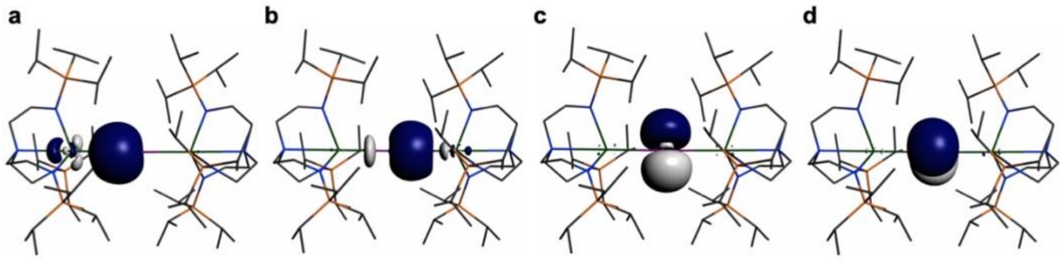

Supplementary Figure 97. Natural Bond Orbitals of **5U**. a) U-Sb σ-bond, b) U-Sb σ-bond, c) U-Sb-U 3c2e π-bond, d) U-Sb-U 3c2e π-bond. H-atoms are omitted for clarity. Plotted at the 0.04 a.u. isosurface level.

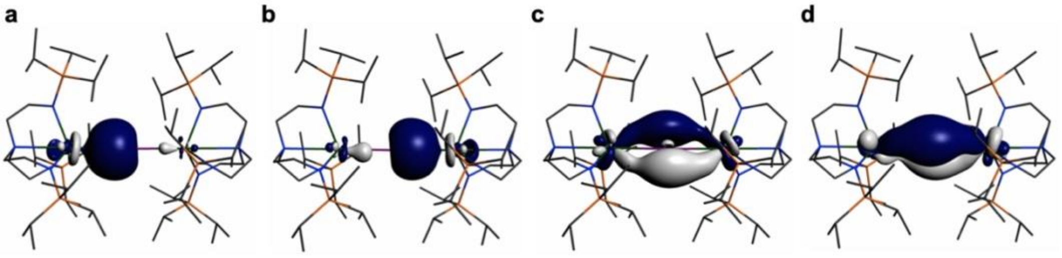

Supplementary Figure 98. Natural Localised Molecular Orbitals of **5U**. a) U-Sb σ-bond, b) U-Sb σ-bond, c) U-Sb-U 3c2e π-bond, d) U-Sb-U 3c2e π-bond. H-atoms are omitted for clarity. Plotted at the 0.04 a.u. isosurface level.

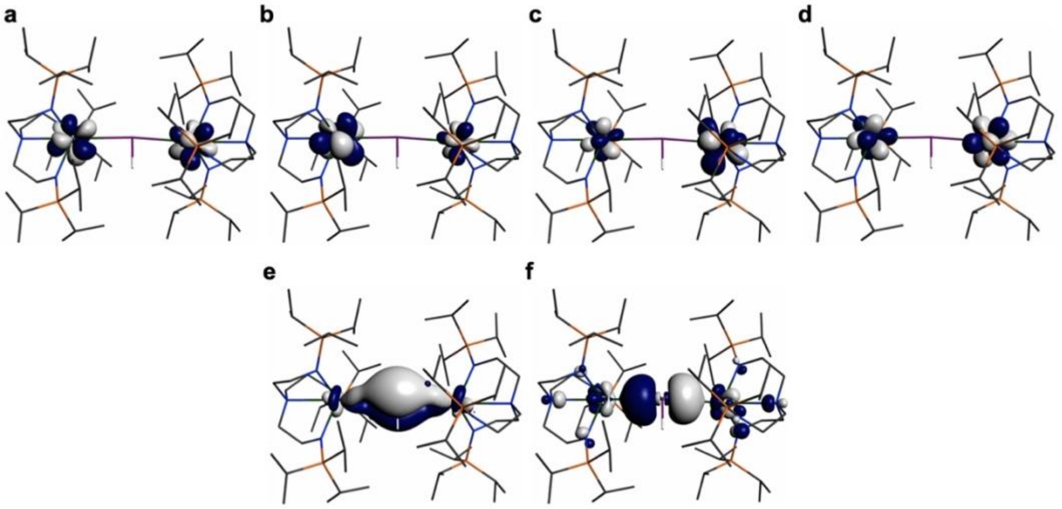

Supplementary Figure 99. Frontier α-spin Kohn Sham Molecular Orbitals of **6U**. a) HOMO (463a, −2.809 eV), b) HOMO−1 (462a, −2.814 eV), c) HOMO−2 (461a, −2.836 eV), d) HOMO−3 (460a, −2.874 eV), e) HOMO−4 (459a, −3.658 eV), f) HOMO−5 (458a, −4.391 eV). Non-Sb bound H-atoms are omitted for clarity. Plotted at the 0.04 a.u. isosurface level.

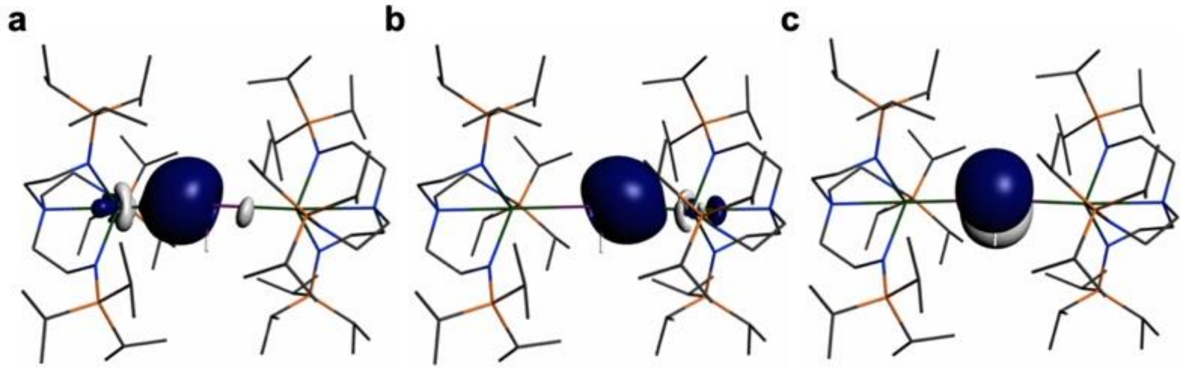

Supplementary Figure 100. Natural Bond Orbitals of **6U**. a) U-Sb σ-bond, b) U-Sb σ-bond, c) U-Sb-U 3c2e π-bond. Non-Sb bound H-atoms are omitted for clarity. Plotted at the 0.04 a.u. isosurface level.

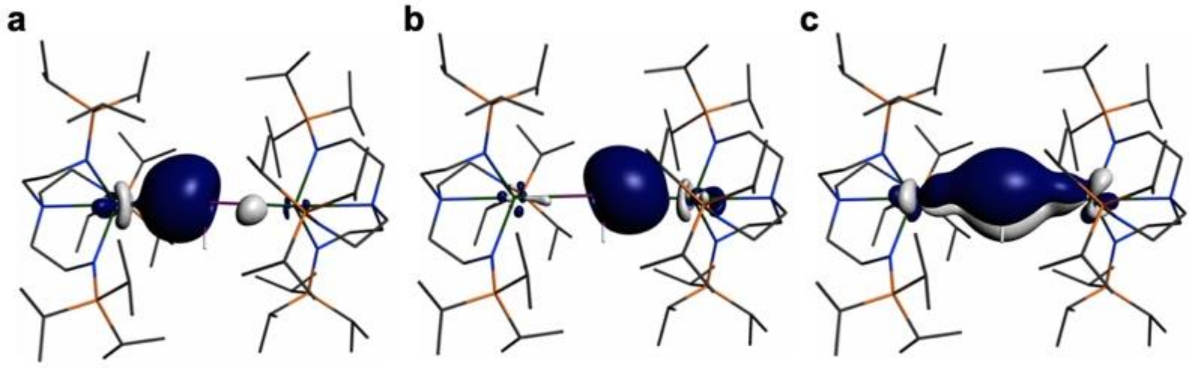

Supplementary Figure 101. Natural Localised Molecular Orbitals of **6U**. a) U-Sb σ-bond, b) U-Sb σ-bond, c) U-Sb-U 3c2e π-bond. Non-Sb bound H-atoms are omitted for clarity. Plotted at the 0.04 a.u. isosurface level.

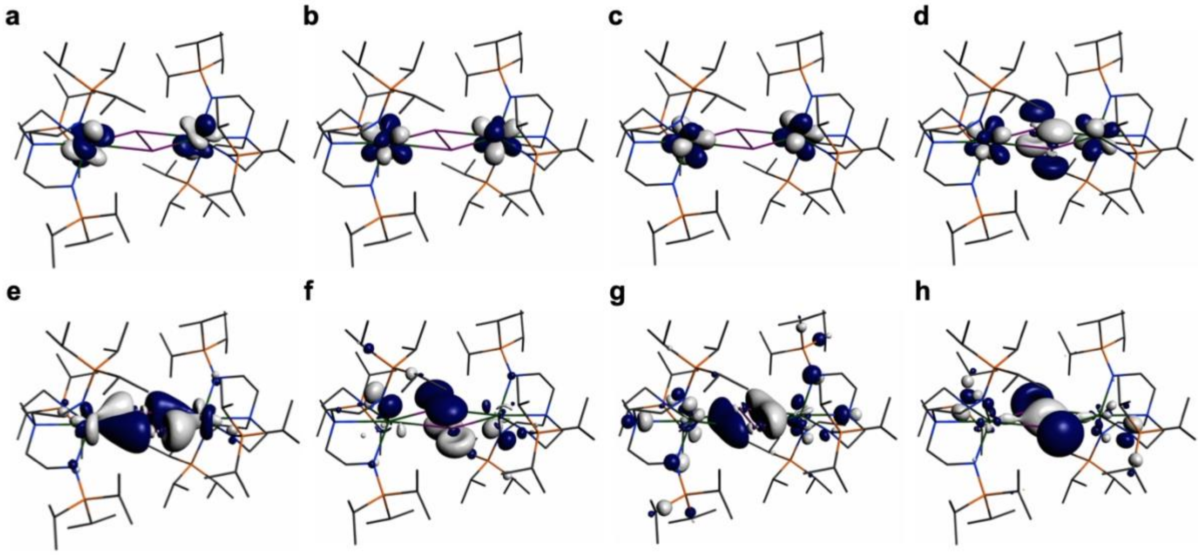

Supplementary Figure 102. Frontier α-spin Kohn Sham Molecular Orbitals of **7U**. a) HOMO (488a, −3.121 eV), b) HOMO−1 (487a, −3.125 eV), c) HOMO−2 (486a, −3.169 eV) , d) HOMO−3 (485a, −3.382 eV), e) HOMO−4 (484a, −4.005 eV), f) HOMO−11 (477a, −5.129 eV), g) HOMO−12 (476a, −5.232 eV), h) HOMO−13 (475a, −5.354 eV). H-atoms are omitted for clarity. Plotted at the 0.04 a.u. isosurface level.

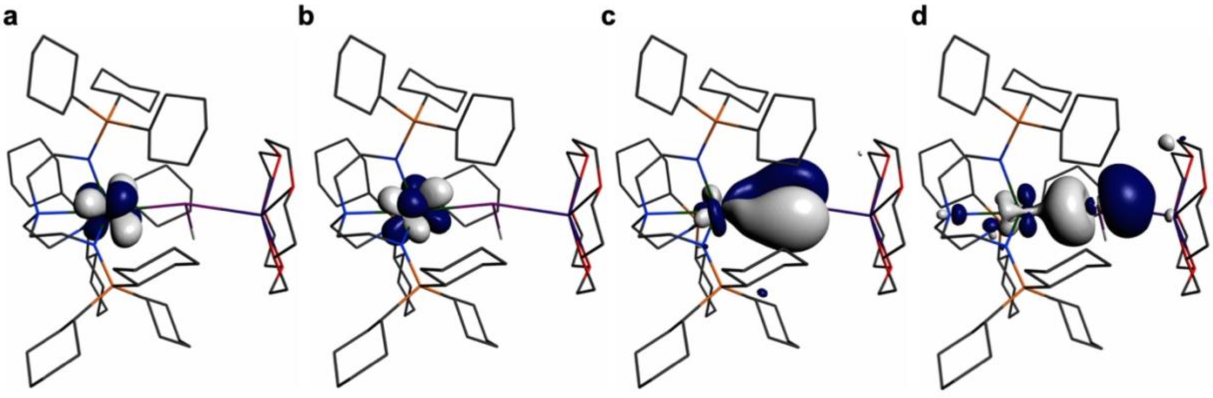

Supplementary Figure 103. Frontier α-spin Kohn Sham Molecular Orbitals of **9UNa**. a) HOMO (409a, −2.024 eV), b) HOMO−1 (408a, −2.072 eV), c) HOMO−2 (407a, −2.664 eV), d) HOMO−3 (406a, −3.271 eV). Non-Sb bound H-atoms are omitted for clarity. Plotted at the 0.04 a.u. isosurface level.

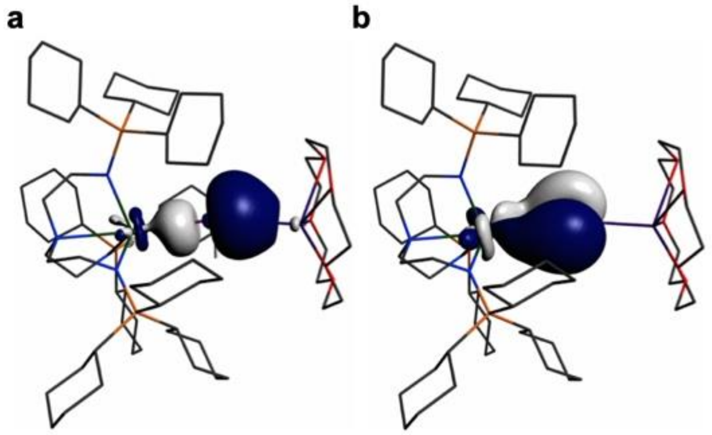

Supplementary Figure 104. Natural Bond Orbitals of **9UNa**. a) U-Sb σ-bond, b) U-Sb π-bond. Non-Sb bound H-atoms are omitted for clarity. Plotted at the 0.04 a.u. isosurface level.

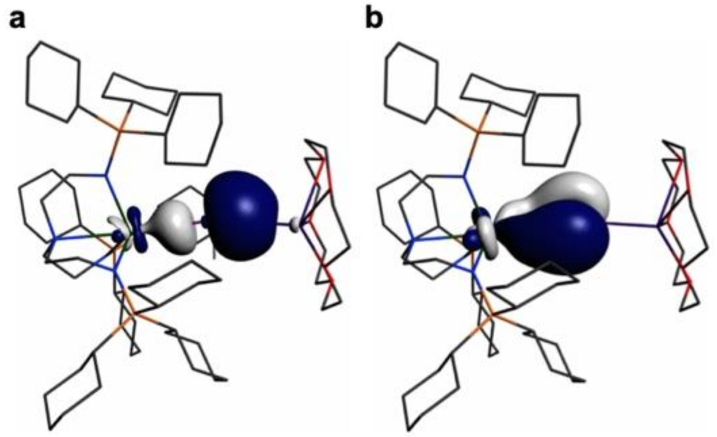

Supplementary Figure 105. Natural Localised Molecular Orbitals of **9UNa**. a) U-Sb σ-bond, b) U-Sb π-bond. Non-Sb bound H-atoms are omitted for clarity. Plotted at the 0.04 a.u. isosurface level.

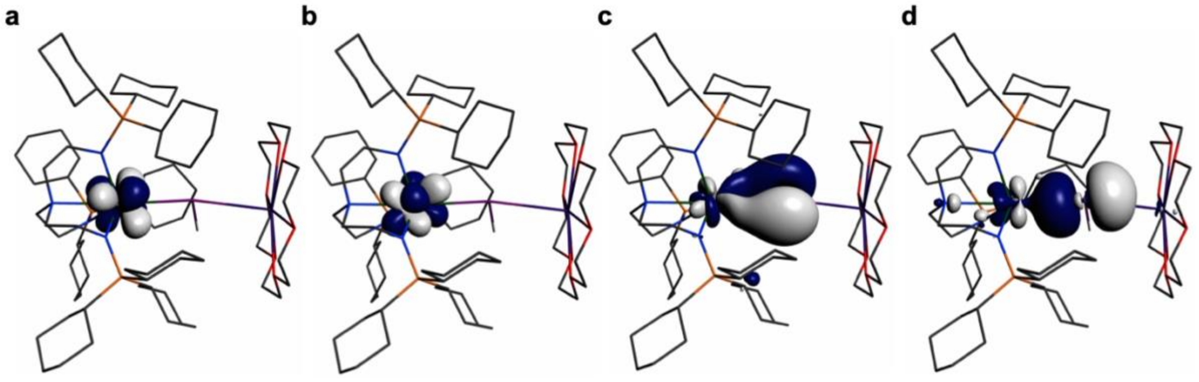

Supplementary Figure 106. Frontier α-spin Kohn Sham Molecular Orbitals of **9UK**. a) HOMO (425a, −1.970 eV), b) HOMO−1 (424a, −2.001 eV), c) HOMO−2 (423a, −571 eV), d) HOMO−3 (422a, −3.075 eV). Non-Sb bound H-atoms are omitted for clarity. Plotted at the 0.04 a.u. isosurface level.

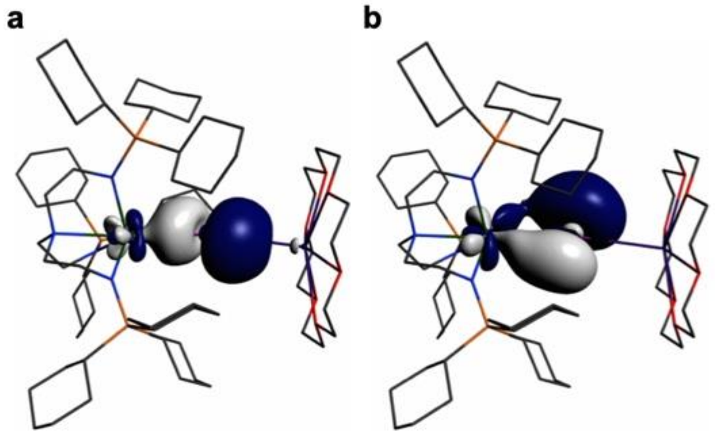

Supplementary Figure 107. Natural Bond Orbitals of **9UK**. a) U-Sb σ-bond, b) U-Sb π-bond. Non-Sb bound H-atoms are omitted for clarity. Plotted at the 0.04 a.u. isosurface level.

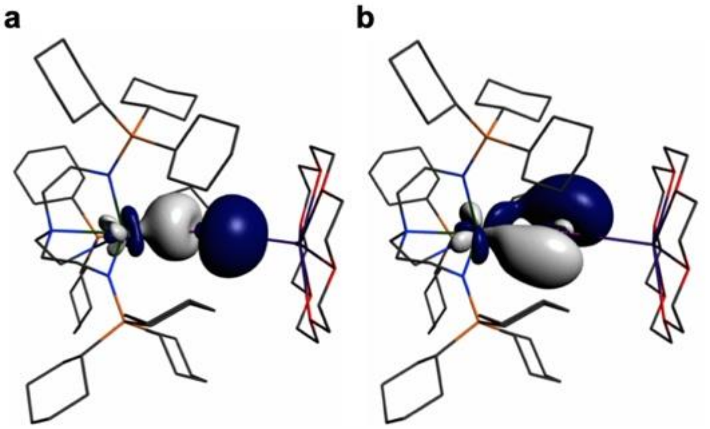

Supplementary Figure 108. Natural Localised Molecular Orbitals of **9UK**. a) U-Sb σ-bond, b) U-Sb π-bond. Non-Sb bound H-atoms are omitted for clarity. Plotted at the 0.04 a.u. isosurface level.

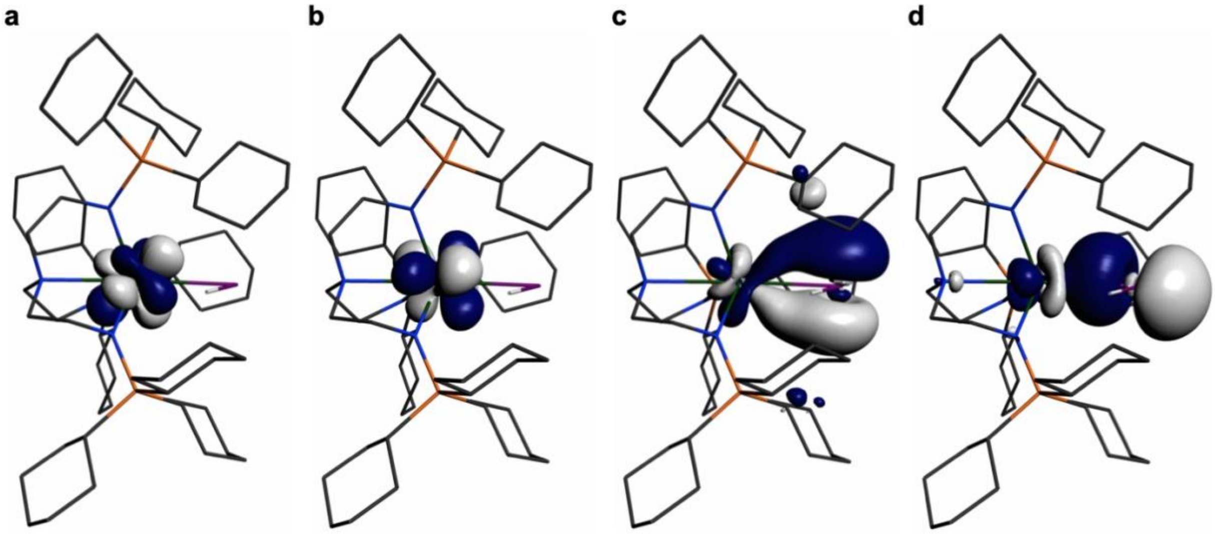

Supplementary Figure 109. Frontier α-spin Kohn Sham Molecular Orbitals of **10U**. a) HOMO (344a, 0.264 eV), b) HOMO−1 (343a, 0.246 eV), c) HOMO−2 (342, −0.350 eV), d) HOMO−3 (341, −0.600 eV). Non-Sb bound H-atoms are omitted for clarity. Plotted at the 0.04 a.u. isosurface level.

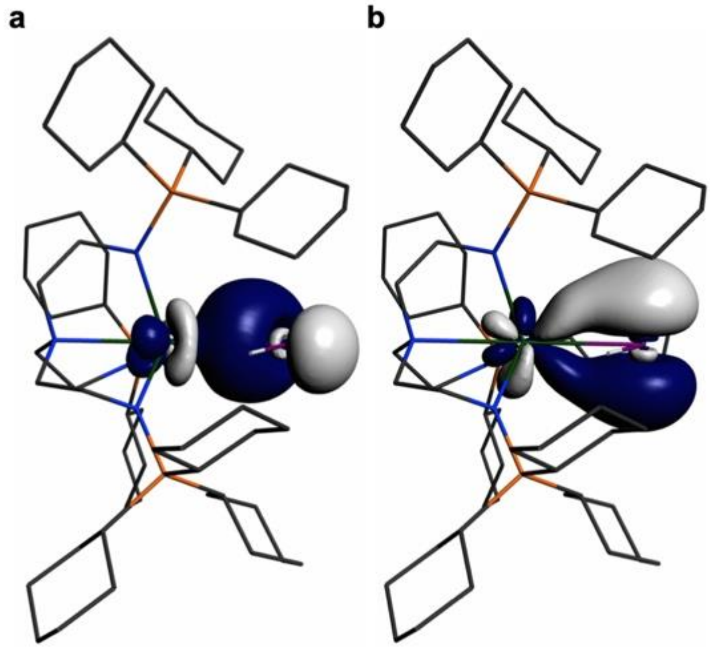

Supplementary Figure 110. Natural Bond Orbitals of **10U**. a) U-Sb σ-bond, b) U-Sb π-bond. Non-Sb bound H-atoms are omitted for clarity. Plotted at the 0.04 a.u. isosurface level.

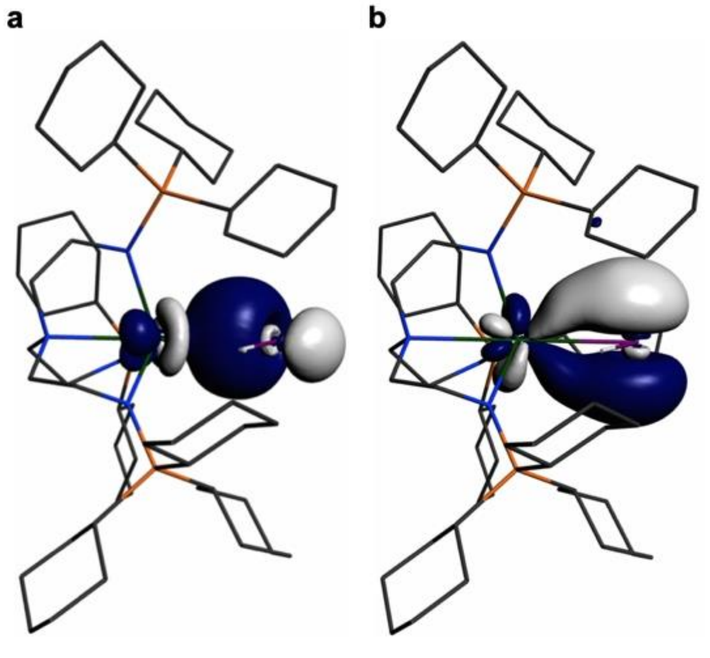

Supplementary Figure 111. Natural Localised Molecular Orbitals of **10U**. a) U-Sb σ-bond, b) U-Sb π-bond. Non-Sb bound H-atoms are omitted for clarity. Plotted at the 0.04 a.u. isosurface level.

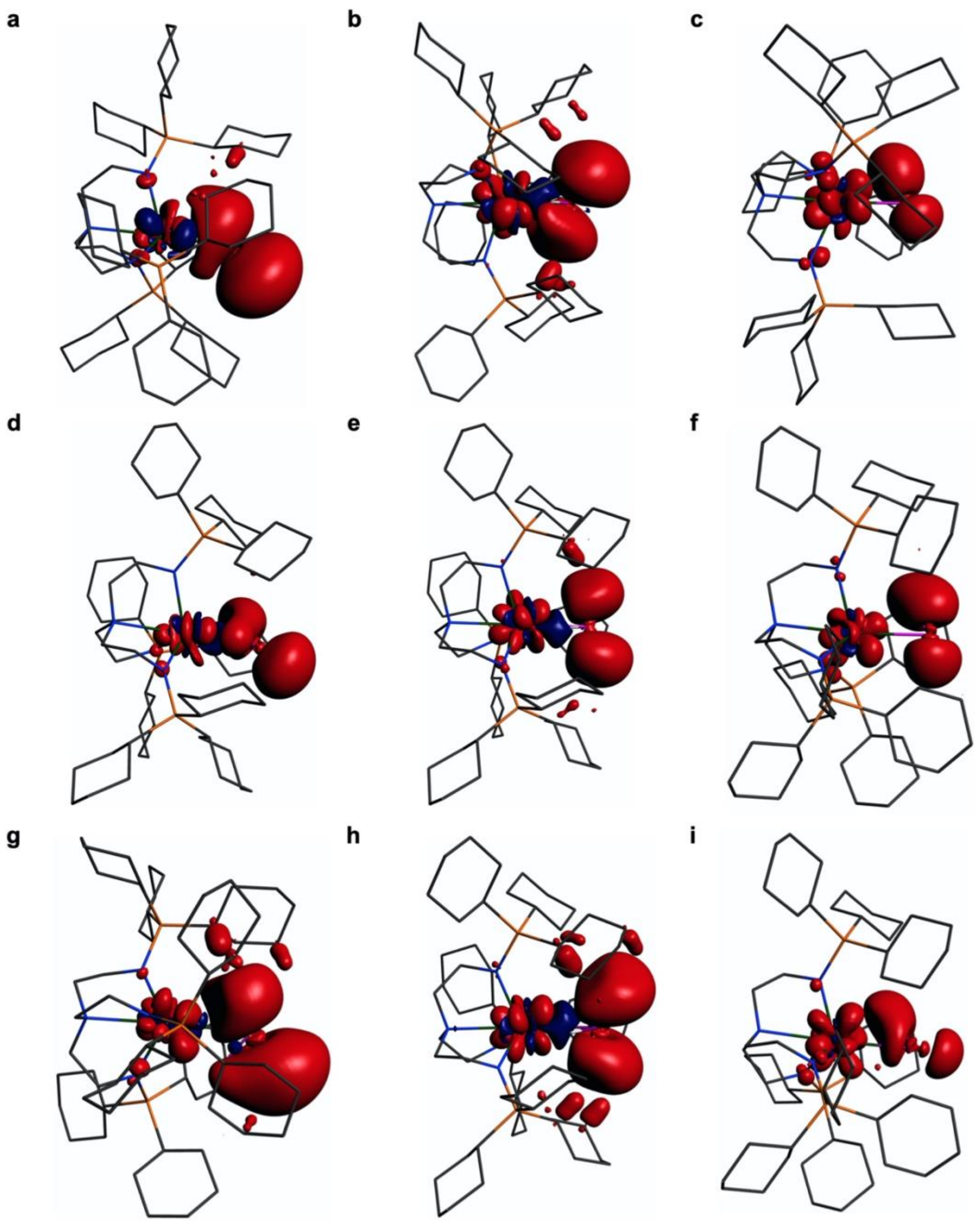

Supplementary Figure 112. ETS-NOCV deformation density plots highlighting the principal interactions in the U=EH (E = P, As, Sb) bonds of **$10U^{P}$**, **$10U^{As}$**, and **10U** , respectively. **a** P→U σ-bond donation in **$10U^{P}$**. **b** P→U π-bond donation in **$10U^{P}$**. **c** P-H→U donation in **$10U^{P}$**. **d** As→U σ-bond donation in **$10U^{As}$**. **e** As→U π-bond donation in **$10U^{As}$**. **f** As-H→U donation in **$10U^{As}$**. **g** Sb→U σ-bond donation in **$10U^{Sb}$**. **h** Sb→U π-bond donation in **$10U^{Sb}$**. **i** Sb-H→U donation in **$10U^{Sb}$**. Charge flow is from red to blue isosurfaces. The deformation density isosurfaces are plotted at 0.001 a.u. and non-Sb bound H-atoms are omitted for clarity.

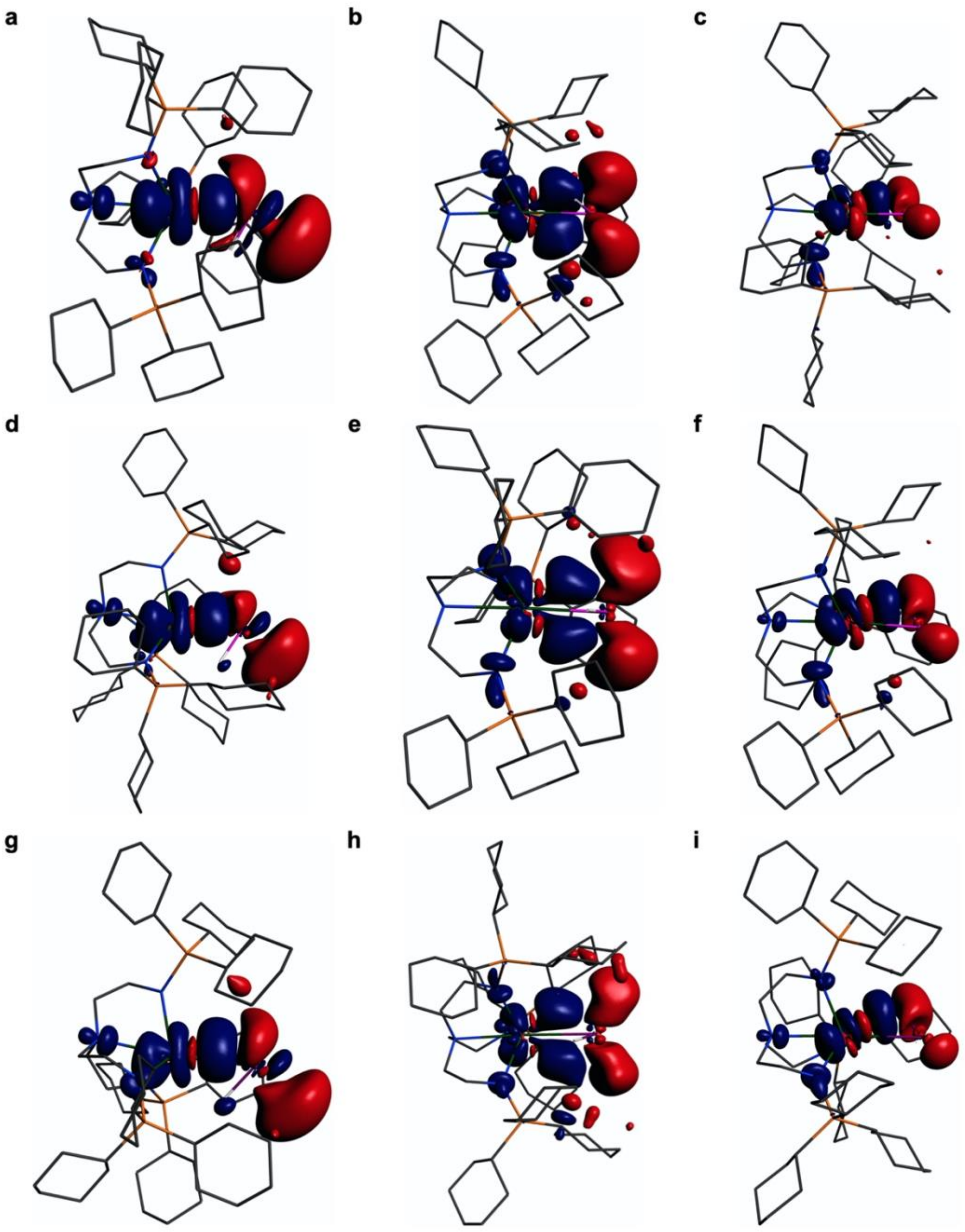

Supplementary Figure 113. ETS-NOCV deformation density plots highlighting the principal interactions in the Th=EH (E = P, As, Sb) bonds of **10Th^{P}**, **10Th^{As}**, and **10Th** , respectively. **a** P→Th σ-bond donation in **10Th^{P}**. **b** P→Th π-bond donation in **10Th^{P}**. **c** P-H→Th donation in **10Th^{P}**. **d** As→Th σ-bond donation in **10Th^{As}**. **e** As→Th π-bond donation in **10Th^{As}**. **f** As-H→Th donation in **10Th^{As}**. **g** Sb→Th σ-bond donation in **10Th^{Sb}**. **h** Sb→Th π-bond donation in **10Th^{Sb}**. **i** Sb-H→Th donation in **10Th^{Sb}**. Charge flow is from red to blue isosurfaces. The deformation density isosurfaces are plotted at 0.001 a.u. and non-Sb bound H-atoms are omitted for clarity.

Tables

Supplementary Table 1. Selected data for the Raman spectroscopic measurements of 7U, 9UK, and 10UK

7U		9UK		10UK	
ν/cm^{-1}	Intensity	ν/cm^{-1}	Intensity	ν/cm^{-1}	Intensity
3048	m	2915	s	2902	s
2922	w	2833	s	2835	s
2884	w	2806	m	2804	m
1589	m	1453	w	1429	w
1046	w	1427	w	1265	vw
1008	m	1327	w	1007	w
158	s	1272	w	794	w
89	m	1178	vw	145	vs
		1026	w	77	s
		797	w		
		135	vs		
		50	s		

Supplementary Table 2. Selected UV/Vis/NIR data for 5UNa, 5UK, and 5UK' [a]

5UNa			5UK			5UK'		
λ_{max} (nm)	ν/cm^{-1}	ε/M^{-1}cm^{-1}	λ_{max} (nm)	ν/cm^{-1}	ε/M^{-1}cm^{-1}	λ_{max} (nm)	ν/cm^{-1}	ε/M^{-1}cm^{-1}
1,208	8,275	607	1,211	8,257	678	1,211	8,257	807
832	12,020	1,506	1,112	8,996	731	1,109	9,019	874
742	13,477	1,469	834	11,996	1,784	839	11,914	2,129
575	17,378	2,278	746	13,400	1,664	748	13,371	1,983
462	21,640	6,418	572	17,471	2,571	580	17,229	2,921
			519	19,271	4,226			
			498	20,086	3,980			

[a] Measurements were conducted in 5 mM solutions in THF.

Supplementary Table 3. Selected UV/Vis/NIR data for 7U and 10UK [a]

7U			10UK		
λ_{max} (nm)	ν/cm^{-1}	ε/M^{-1}cm^{-1}	λ_{max} (nm)	ν/cm^{-1}	ε/M^{-1}cm^{-1}
1,135	8,811	357	1,219	8,205	197
745	13,417	1,323	1,111	9,003	293
692	14,448	1502	964	10,378	458
517	19,327	2,534	723	13,826	501
472	21,164	4,250	546	18,295	802

[a] Measurements were conducted in 5 mM solutions in THF, except for **10UK** which was 10 mM in THF.

Supplementary Table 4. Selected experimental data for the IR spectroscopic measurements of 5UNa, 5UK, and 5UK'

**5UNa**		**5UK**		**5UK'**	
**ν/cm^{-1}**	**Intensity**	**ν/cm^{-1}**	**Intensity**	**ν/cm^{-1}**	**Intensity**
2932	m	2911	m	2934	w
2856	s	2855	s	2880	m
1459	s	1456	m	2854	s
1383	w	1381	w	1458	m
1352	w	1352	w	1445	m
1333	w	1335	w	1381	w
1290	w	1274	w	1356	w
1288	w	1249	w	1335	w
1261	w	1105	s	1296	w
1243	s	1043	s	1259	w
1115	s	1016	m	1133	s
1093	m	962	m	1104	m
1041	s	933	s	1044	s
1013	w	880	s	1016	m
993	s	786	m	931	s
881	s	735	vs	880	s
787	vs	671	s	784	s
735	s	625	m	732	vs
669	s	567	w	670	s
567	w	515	w	629	m
				567	w
				515	w
				436	w

Supplementary Table 5. Selected experimental data for the IR spectroscopic measurements of 6U, 6U/7U, and 8U

6U		7U:6U		8U	
ν/cm^{-1}	Intensity	ν/cm^{-1}	Intensity	ν/cm^{-1}	Intensity
2941	m	2941	m	2924	m
2887	m	2887	m	2850	m
2860	m	2860	m	1459	w
2842	m	2842	m	1461	s
1459	m	1459	m	1399	w
1381	w	1381	w	1379	w
1362	w	1362	w	1241	m
1336	w	1336	w	1109	m
1271	w	1271	w	1054	m
1253	w	1253	w	990	m
1140	m	1140	m	931	m
1040	m	1040	m	879	s
1035	m	1035	m	830	w
1010	m	1010	m	795	w
929	s	929	s	731	m
880	s	880	s	665	w
795	m	795	m	647	w
720	vs	720	vs	628	w
673	m	673	m	548	w
628	m	628	m	507	w
569	w	569	w		
517	w	517	w		
442	w	442	w		

Supplementary Table 6. Selected experimental data for the IR spectroscopic measurements of 9UNa, 9UK, and 10UK

**9UNa**		**9UK**		**10UK**	
**ν/cm^{-1}**	**Intensity**	**ν/cm^{-1}**	**Intensity**	**ν/cm^{-1}**	**Intensity**
2912	s	2909	s	2908	s
2840	s	2839	s	2839	s
1574	w	1654	w	1653	w
1442	m	1444	m	1443	m
1396	m	1350	w	1350	w
1352	w	1272	w	1273	w
1290	w	1248	w	1248	w
1255	m	1107	s	1109	vs
1241	w	1044	w	1044	w
1189	m	997	m	998	m
1165	m	963	s	963	s
1115	s	931	w	933	w
1103	m	889	w	889	w
1099	s	840	w	840	w
1037	m	817	w	817	w
996	m	796	w	797	w
933	m	743	vs	742	vs
910	w	678	w	678	w
889	s	552	m	552	m
844	m	521	m	521	m
813	m	452	w	454	w
760	w	437	w		
731	w				
514	s				
450	w				
429	w				

Supplementary Table 7. Computed bond length, indices, charges, and spin density data for 5U (anion of 5UK), 6U, 7U, 9UNa, 9UK, and 10U (anion of 10UK)

Cmpd	U-Sb bond lengths and indices		MDC_q charges		MDC_m spin density[b]	
	Dist.	BI[a]	U	Sb	U	Sb
**5U**	3.0253	1.92	2.30	−0.71	2.48	−0.42
	3.0614	1.80	2.37		2.44	
**6U**	3.1318	1.24	2.69	−1.15	2.39	−0.19
	3.1457	1.22	2.76		2.39	
**7U**	3.2906	0.90	2.34	−0.38	2.22	0.06
	3.3007	0.83	2.34	−0.38	2.22	0.06
	3.3008	0.83				
	3.2907	0.90				
**9UNa**	3.0408	1.79	2.22	−0.46	2.50	−0.21
**9UK**	3.0696	1.80	1.99	−0.82	2.54	−0.25
**10U**	2.9950	2.17	1.80	−0.41	2.55	−0.32

[a] Nalewajski-Mrozek bond indices. [b] Positive value is accumulation of electron spin density, negative value is a deficit of electron spin density.

Supplementary Table 8. Natural Bond Orbital data for the U-Sb bonds in 5U (anion of 5UK), 6U, 9UNa, 9UK, and 10U (anion of 10UK)[a]

Cmpd	U-Sb σ-bond (%)				U-Sb π-bond (%)			
	U	Sb	U s/p/d/f	Sb s/p	U	Sb	U s/p/d/f	Sb s/p
**5U**	15	85	17/0/63/20	55/45	0	100	0/0/0/0	0/100
	15	85	17/0/63/20	55/45	0	100	0/0/0/0	0/100
**6U**	12	88	19/0/62/19	51/49	0	100	0/0/0/0	0/100
	13	87	18/0/62/30	38/62				
**9UNa**	7	93	6/0/56/38	21/79	28	72	1/0/34/65	3/97
**9UK**	10	90	3/0/50/47	7/93	29	71	0/0/31/69	7/93
**10U**	25	75	1/0/35/64	13/87	38	62	0/0/24/76	0/100

[a] Complex **7U** was not examined by the NBO method given the four-centre nature of the U_2Sb_2 core.

Supplementary Table 9. Natural Localised Molecular Orbital data for the U-Sb bonds in 5U (anion of 5UK), 6U, 9UNa, 9UK, and 10U (anion of 10UK)[a]

**Cmpd**	**U-Sb σ-bond (%)**				**U-Sb π-bond (%)**			
	**U**	**Sb**	**U s/p/d/f**	**Sb s/p**	**U**	**Sb**	**U s/p/d/f**	**Sb s/p**
**5U**	19	80	10/0/47/43	47/53	22+22	53	0/0/30/70	0/100
	19	80	12/0/54/34	55/45	23+23	50	0/0/30/70	0/100
**6U**	15	84	10/0/57/33	38/62	17+18	62	0/0/34/66	2/98
	15	84	11/0/57/32	52/48				
**9UNa**	8	89	3/0/42/55	24/76	28	69	0/0/27/73	4/96
**9UK**	10	87	0/0/36/64	9/91	28	69	0/0/25/75	1/99
**10U**	24	73	1/0/34/65	16/84	37	60	0/0/25/75	0/100

[a] Complex **7U** was not examined by the NLMO method given the four-centre nature of the U_2Sb_2 core.

Supplementary Table 10. Quantum Theory of Atoms In Molecules data for the M-Sb (3, –1)-bond critical points in 5U (anion of 5UK), 6U, 7U, 9UNa, 9UK, and 10U (anion of 10UK)

**Cmpd**	ρ^{a}	$\nabla^2\rho^{b}$	H^{c}	ε^{d}
**5U**	0.05	0.05	–0.01	0.06
	0.05	0.05	–0.01	0.07
**6U**	0.04	0.04	–0.01	0.28
	0.04	0.04	–0.01	0.25
**7U**	0.03	0.04	–0.01	0.12
	0.03	0.03	–0.01	0.10
	0.03	0.03	–0.01	0.10
	0.03	0.04	–0.01	0.12
**9UNa**	0.05	0.04	–0.02	0.31
**9UK**	0.05	0.04	–0.02	0.28
**10U**	0.05	0.05	–0.02	0.23

[a] Topological electron density. [b] Laplacian. [c] Electronic energy density. [d] Bond ellipticity.

Crystallographic Alerts and Justifications

5UK

Alert level A

PLAT971_ALERT_2_A Check Calcd Resid. Dens. 2.23Ang From C46 9.29 eA-3

Author Response: Residual density is either due to unresolved pseudosymmetry (See cif details) or close to a heavy atom.

PLAT971_ALERT_2_A Check Calcd Resid. Dens. 1.01Ang From C63 8.19 eA-3

Author Response: Residual density is either due to unresolved pseudosymmetry (See cif details) or close to a heavy atom.

PLAT971_ALERT_2_A Check Calcd Resid. Dens. 1.96Ang From C24 4.62 eA-3

Author Response: Residual density is either due to unresolved pseudosymmetry (See cif details) or close to a heavy atom.

PLAT971_ALERT_2_A Check Calcd Resid. Dens. 0.56Ang From U2 3.88 eA-3

Author Response: Residual density is either due to unresolved pseudosymmetry (See cif details) or close to a heavy atom.

PLAT971_ALERT_2_A Check Calcd Resid. Dens. 0.36Ang From U1 3.52 eA-3

Author Response: Residual density is either due to unresolved pseudosymmetry (See cif details) or close to a heavy atom.

PLAT972_ALERT_2_A Check Calcd Resid. Dens. 0.70Ang From U1 -3.76 eA-3

Author Response: Residual density is either due to unresolved pseudosymmetry (See cif details) or close to a heavy atom.

Alert level B

PLAT201_ALERT_2_B Isotropic non-H Atoms in Main Residue(s) .... 1 Report C63

Author Response: C63 was refined isotropically as chemically sensible displacement parameters could not be obtained due it its proximity to a pseudosymmetry heavy atom site.

PLAT972_ALERT_2_B Check Calcd Resid. Dens. 0.71Ang From U1 -3.09 eA-3

Author Response: Residual density is either due to unresolved pseudosymmetry (See cif details) or close to a heavy atom.

PLAT972_ALERT_2_B Check Calcd Resid. Dens. 0.87Ang From U1 -3.01 eA-3

Author Response: Residual density is either due to unresolved pseudosymmetry (See cif details) or close to a heavy atom.

PLAT972_ALERT_2_B Check Calcd Resid. Dens. 0.79Ang From U1 -3.01 eA-3

Author Response: Residual density is either due to unresolved pseudosymmetry (See cif details) or close to a heavy atom.

PLAT972_ALERT_2_B Check Calcd Resid. Dens. 0.80Ang From U1 -3.01 eA-3

Author Response: Residual density is either due to unresolved pseudosymmetry (Secif e details) or close to a heavy atom.

PLAT972_ALERT_2_B Check Calcd Resid. Dens. 0.82Ang From U2 -2.99 eA-3

Author Response: Residual density is either due to unresolved pseudosymmetry (See cif details) or close to a heavy atom.

PLAT972_ALERT_2_B Check Calcd Resid. Dens. 0.84Ang From U2 -2.85 eA-3

Author Response: Residual density is either due to unresolved pseudosymmetry (See cif details) or close to a heavy atom.

PLAT972_ALERT_2_B Check Calcd Resid. Dens. 0.80Ang From U2 -2.81 eA-3

Author Response: Residual density is either due to unresolved pseudosymmetry (See cif details) or close to a heavy atom.

PLAT972_ALERT_2_B Check Calcd Resid. Dens. 0.69Ang From U2 -2.73 eA-3

Author Response: Residual denisty is either due to unresolved pseudosymmetry (Seecif details) or close to a heavy atom.

PLAT972_ALERT_2_B Check Calcd Resid. Dens. 0.88Ang From U2 -2.60 eA-3

Author Response: Residual density is either due to unresolved pseudosymmetry (See cif details) or close to a heavy atom.

5UK'

No Alerts at level A or B.

5UNa

Alert level B

PLAT910_ALERT_3_B Missing # of FCF Reflection(s) Below Theta(Min). 50 Note

2 0 0, 1 1 0, 2 1 0, 0 2 0, 1 2 0, 2 2 0,

1 3 0, 2 3 0, 0 4 0, 1 4 0, -1 0 1, 1 0 1,

-2 1 1, -1 1 1, 0 1 1, 1 1 1, 2 1 1, -2 2 1,

-1 2 1, 0 2 1, 1 2 1, 2 2 1, -1 3 1, 0 3 1,

Author Response: Missing reflections at low angle are due to obfuscation by the beamstop, and are present despite applying beamstop corrections.

6U

Alert level A

PLAT971_ALERT_2_A Check Calcd Resid. Dens. 0.98Ang From U1 4.53 eA-3

Author Response: Residual density is close to a heavy atom and present despite applying absorption corrections. It is not chemically significant.

PLAT971_ALERT_2_A Check Calcd Resid. Dens. 0.99Ang From U2 4.45 eA-3

Author Response: Residual density is close to a heavy atom and present despite applying absorption corrections. It is not chemically significant.

PLAT971_ALERT_2_A Check Calcd Resid. Dens. 0.94Ang From U2 4.14 eA-3

Author Response: Residual density is close to a heavy atom and present despite applying absorption corrections. It is not chemically significant.

PLAT971_ALERT_2_A Check Calcd Resid. Dens. 1.01Ang From U2 4.05 eA-3

Author Response: Residual density is close to a heavy atom and present despite applying absorption corrections. It is not chemically significant.

PLAT971_ALERT_2_A Check Calcd Resid. Dens. 0.97Ang From U1 3.93 eA-3

Author Response: Residual density is close to a heavy atom and present despite applying absorption corrections. It is not chemically significant.

PLAT971_ALERT_2_A Check Calcd Resid. Dens. 0.83Ang From U1 3.82 eA-3

Author Response: Residual density is close to a heavy atom and present despite applying absorption corrections. It is not chemically significant.

PLAT971_ALERT_2_A Check Calcd Resid. Dens. 1.05Ang From U2 3.62 eA-3

Author Response: Residual density is close to a heavy atom and present despite applying absorption corrections. It is not chemically significant.

PLAT971_ALERT_2_A Check Calcd Resid. Dens. 0.88Ang From U2 3.58 eA-3

Author Response: Residual density is close to a heavy atom and present despite applying absorption corrections. It is not chemically significant.

PLAT971_ALERT_2_A Check Calcd Resid. Dens. 1.13Ang From U2 3.58 eA-3

Author Response: Residual density is close to a heavy atom and present despite applying absorption corrections. It is not chemically significant.

PLAT972_ALERT_2_A Check Calcd Resid. Dens. 0.79Ang From U1 -5.11 eA-3

Author Response: Residual density is close to a heavy atom and present despite applying absorption corrections. It is not chemically significant.

PLAT972_ALERT_2_A Check Calcd Resid. Dens. 0.82Ang From U2 -4.23 eA-3

Author Response: Residual density is close to a heavy atom and present despite applying absorption corrections. It is not chemically significant.

PLAT972_ALERT_2_A Check Calcd Resid. Dens. 0.94Ang From U2 -4.11 eA-3

Author Response: Residual density is close to a heavy atom and present despite applying absorption corrections. It is not chemically significant.

PLAT972_ALERT_2_A Check Calcd Resid. Dens. 0.77Ang From U1 -3.75 eA-3

Author Response: Residual density is close to a heavy atom and present despite applying absorption corrections. It is not chemically significant.

PLAT976_ALERT_2_A Check Calcd Resid. Dens. 1.06Ang From N2 . -2.21 eA-3

Author Response: Residual density is close to a heavy atom and present despite applying absorption corrections. It is not chemically significant.

Alert level B

PLAT971_ALERT_2_B Check Calcd Resid. Dens. 1.07Ang From U1 3.29 eA-3

Author Response: Residual density is close to a heavy atom and present despite applying absorption corrections. It is not chemically significant.

PLAT972_ALERT_2_B Check Calcd Resid. Dens. 0.92Ang From U2 -3.41 eA-3

Author Response: Residual density is close to a heavy atom and present despite applying absorption corrections. It is not chemically significant.

PLAT972_ALERT_2_B Check Calcd Resid. Dens. 0.90Ang From U1 -3.37 eA-3

Author Response: Residual density is close to a heavy atom and present despite applying absorption corrections. It is not chemically significant.

PLAT972_ALERT_2_B Check Calcd Resid. Dens. 0.99Ang From U1 -3.17 eA-3

Author Response: Residual density is close to a heavy atom and present despite applying absorption corrections. It is not chemically significant.

PLAT972_ALERT_2_B Check Calcd Resid. Dens. 0.93Ang From U2 -3.10 eA-3

Author Response: Residual density is close to a heavy atom and present despite applying absorption corrections. It is not chemically significant.

7U

Alert level A

PLAT308_ALERT_2_A Single Bonded Metal Atom in Structure (Unusual) Sb1 Check

Author Response: Sb1 is bonded to two U centres and to another Sb atom.

PLAT308_ALERT_2_A Single Bonded Metal Atom in Structure (Unusual) Sb1A Check

Author Response: Sb1 is bonded to two U centres and to another Sb atom.

PLAT971_ALERT_2_A Check Calcd Resid. Dens. 0.81Ang From U1 3.68 eA-3

Author Response: Residual density is close to a heavy atom and present despite applying absorption corrections. It is not chemically significant.

PLAT971_ALERT_2_A Check Calcd Resid. Dens. 0.78Ang From U1 3.60 eA-3

Author Response: Residual density is close to a heavy atom and present despite applying absorption corrections. It is not chemically significant.

PLAT972_ALERT_2_A Check Calcd Resid. Dens. 0.80Ang From U1 -4.95 eA-3

Author Response: Residual density is close to a heavy atom and present despite applying absorption corrections. It is not chemically significant.

PLAT972_ALERT_2_A Check Calcd Resid. Dens. 0.76Ang From U1 -4.70 eA-3

Author Response: Residual density is close to a heavy atom and present despite applying absorption corrections. It is not chemically significant.

PLAT972_ALERT_2_A Check Calcd Resid. Dens. 0.84Ang From U1 -4.69 eA-3

Author Response: Residual density is close to a heavy atom and present despite applying absorption corrections. It is not chemically significant.

PLAT972_ALERT_2_A Check Calcd Resid. Dens. 1.01Ang From U1 -4.60 eA-3

Author Response: Residual density is close to a heavy atom and present despite applying absorption corrections. It is not chemically significant.

Alert level B

PLAT971_ALERT_2_B Check Calcd Resid. Dens. 1.27Ang From U1 2.83 eA-3

Author Response: Residual density is close to a heavy atom and present despite applying absorption corrections. It is not chemically significant.

PLAT971_ALERT_2_B Check Calcd Resid. Dens. 1.26Ang From U1 2.53 eA-3

Author Response: Residual density is close to a heavy atom and present despite applying absorption corrections. It is not chemically significant.

8U

Alert level B

PLAT971_ALERT_2_B Check Calcd Resid. Dens. 1.02Ang From U1 2.66 eA-3

Author Response: Residual density is close to a heavy atom and present despite applying absorption corrections. It is not chemically significant.

PLAT971_ALERT_2_B Check Calcd Resid. Dens. 1.02Ang From U1 2.55 eA-3

Author Response: Residual density is close to a heavy atom and present despite applying absorption corrections. It is not chemically significant.

PLAT973_ALERT_2_B Check Calcd Positive Resid. Density on U1 1.69 eA-3

Author Response: Residual density is close to a heavy atom and present despite applying absorption corrections. It is not chemically significant.

9UK

Alert level B

PLAT972_ALERT_2_B Check Calcd Resid. Dens. 0.79Ang From U1 -2.96 eA-3

Author Response: Residual density is close to a heavy atom and present despite applying absorption corrections. It is not chemically significant.

PLAT972_ALERT_2_B Check Calcd Resid. Dens. 0.89Ang From U1 -2.93 eA-3

Author Response: Residual density is close to a heavy atom and present despite applying absorption corrections. It is not chemically significant.

PLAT972_ALERT_2_B Check Calcd Resid. Dens. 0.71Ang From U1 -2.88 eA-3

Author Response: Residual density is close to a heavy atom and present despite applying absorption corrections. It is not chemically significant.

PLAT972_ALERT_2_B Check Calcd Resid. Dens. 0.71Ang From U1 -2.88 eA-3

Author Response: Residual density is close to a heavy atom and present despite applying absorption corrections. It is not chemically significant.

PLAT972_ALERT_2_B Check Calcd Resid. Dens. 0.85Ang From U1 -2.82 eA-3

Author Response: Residual density is close to a heavy atom and present despite applying absorption corrections. It is not chemically significant.

PLAT972_ALERT_2_B Check Calcd Resid. Dens. 0.86Ang From U1 -2.79 eA-3

Author Response: Residual density is close to a heavy atom and present despite applying absorption corrections. It is not chemically significant.

PLAT972_ALERT_2_B Check Calcd Resid. Dens. 0.78Ang From U1 -2.74 eA-3

Author Response: Residual density is close to a heavy atom and present despite applying absorption corrections. It is not chemically significant.

PLAT972_ALERT_2_B Check Calcd Resid. Dens. 0.99Ang From U1 -2.65 eA-3

Author Response: Residual density is close to a heavy atom and present despite applying absorption corrections. It is not chemically significant.

9UNa

Alert level A

PLAT971_ALERT_2_A Check Calcd Resid. Dens. 0.92Ang From Sb2 3.63 eA-3

Author Response: Residual density is close to a heavy atom and present despite applying absorption corrections. It is not chemically significant.

PLAT972_ALERT_2_A Check Calcd Resid. Dens. 0.89Ang From U1 -4.93 eA-3

Author Response: Residual density is close to a heavy atom and present despite applying absorption corrections. It is not chemically significant.

PLAT972_ALERT_2_A Check Calcd Resid. Dens. 0.88Ang From Sb2 -4.69 eA-3

Author Response: Residual density is close to a heavy atom and present despite applying absorption corrections. It is not chemically significant.

PLAT972_ALERT_2_A Check Calcd Resid. Dens. 0.90Ang From U2 -3.95 eA-3

Author Response: Residual density is close to a heavy atom and present despite applying absorption corrections. It is not chemically significant.

PLAT972_ALERT_2_A Check Calcd Resid. Dens. 0.91Ang From U1 -3.77 eA-3

Author Response: Residual density is close to a heavy atom and present despite applying absorption corrections. It is not chemically significant.

PLAT972_ALERT_2_A Check Calcd Resid. Dens. 0.55Ang From Sb1 -3.72 eA-3

Author Response: Residual density is close to a heavy atom and present despite applying absorption corrections. It is not chemically significant.

PLAT972_ALERT_2_A Check Calcd Resid. Dens. 0.95Ang From U1 -3.71 eA-3

Author Response: Residual density is close to a heavy atom and present despite applying absorption corrections. It is not chemically significant.

PLAT972_ALERT_2_A Check Calcd Resid. Dens. 0.56Ang From Sb1 -3.62 eA-3

Author Response: Residual density is close to a heavy atom and present despite applying absorption corrections. It is not chemically significant.

PLAT972_ALERT_2_A Check Calcd Resid. Dens. 0.89Ang From U2 -3.55 eA-3

Author Response: Residual density is close to a heavy atom and present despite applying absorption corrections. It is not chemically significant.

Alert level B

PLAT342_ALERT_3_B Low Bond Precision on C-C Bonds .... 0.02283 Ang.

Author Response: Low bond precision is due to disordered groups.

PLAT972_ALERT_2_B Check Calcd Resid. Dens. 0.50Ang From Sb1 -3.42 eA-3

Author Response: Residual density is close to a heavy atom and present despite applying absorption corrections. It is not chemically significant.

PLAT972_ALERT_2_B Check Calcd Resid. Dens. 0.90Ang From U2 -3.36 eA-3

Author Response: Residual density is close to a heavy atom and present despite applying absorption corrections. It is not chemically significant.

PLAT972_ALERT_2_B Check Calcd Resid. Dens. 0.89Ang From U2 -3.32 eA-3

Author Response: Residual density is close to a heavy atom and present despite applying absorption corrections. It is not chemically significant.

PLAT972_ALERT_2_B Check Calcd Resid. Dens. 0.31Ang From Sb2 -3.17 eA-3

Author Response: Residual density is close to a heavy atom and present despite applying absorption corrections. It is not chemically significant.

PLAT972_ALERT_2_B Check Calcd Resid. Dens. 1.13Ang From U1 -2.88 eA-3

Author Response: Residual density is close to a heavy atom and present despite applying absorption corrections. It is not chemically significant.

PLAT972_ALERT_2_B Check Calcd Resid. Dens. 0.53Ang From Sb1 -2.87 eA-3

Author Response: Residual density is close to a heavy atom and present despite applying absorption corrections. It is not chemically significant.

PLAT972_ALERT_2_B Check Calcd Resid. Dens. 0.90Ang From U1 -2.85 eA-3

Author Response: Residual density is close to a heavy atom and present despite applying absopbtion corrections. It is not chemically significant.

PLAT972_ALERT_2_B Check Calcd Resid. Dens. 1.69Ang From U1 -2.80 eA-3

Author Response: Residual density is close to a heavy atom and present despite applying absorption corrections. It is not chemically significant.

PLAT972_ALERT_2_B Check Calcd Resid. Dens. 1.18Ang From N2 -2.75 eA-3

Author Response: Residual density is close to a heavy atom and present despite applying absorption corrections. It is not chemically significant.

PLAT972_ALERT_2_B Check Calcd Resid. Dens. 1.79Ang From U2 -2.67 eA-3

Author Response: Residual density is close to a heavy atom and present despite applying absorption corrections. It is not chemically significant.

PLAT972_ALERT_2_B Check Calcd Resid. Dens. 0.99Ang From N7 -2.66 eA-3

Author Response: Residual density is close to a heavy atom and present despite applying absorption corrections. It is not chemically significant.

PLAT972_ALERT_2_B Check Calcd Resid. Dens. 1.07Ang From U1 -2.59 eA-3

Author Response: Residual density is close to a heavy atom and present despite applying absorption corrections. It is not chemically significant.

10UK

Alert level A

PLAT972_ALERT_2_A Check Calcd Resid. Dens. 0.69Ang From U1 -5.16 eA-3

Author Response: Residual density is close to a heavy atom and present despite applying absorption corrections. It is not chemically significant.

PLAT972_ALERT_2_A Check Calcd Resid. Dens. 0.68Ang From U1 -3.74 eA-3

Author Response: Residual density is close to a heavy atom and present despite applying absorption corrections. It is not chemically significant.

Alert level B

PLAT972_ALERT_2_B Check Calcd Resid. Dens. 0.77Ang From U1 -3.30 eA-3

Author Response: Residual density is close to a heavy atom and present despite applying absorption corrections. It is not chemically significant.

PLAT972_ALERT_2_B Check Calcd Resid. Dens. 0.60Ang From U1 -2.86 eA-3

Author Response: Residual density is close to a heavy atom and present despite applying absorption corrections. It is not chemically significant.

PLAT973_ALERT_2_B Check Calcd Positive Resid. Density on U1 1.53 eA-3

Author Response: Residual density is close to a heavy atom and present despite applying absorption corrections. It is not chemically significant.
