# Supplementary material for: Uranium-stibinidiide, -stibinidene, and -stibido multiple bonds and uranium-nitride formation from multimetallic diuranium-distibene-mediated dinitrogen cleavage
Source: Nat Commun. 2025 Aug 4;16:7136. doi: 10.1038/s41467-025-61612-5 (PMC12322283; doi:10.1038/s41467-025-61612-5)
Supplement: Supplementary file 3 — Source Data [file 41467_2025_61612_MOESM3_ESM.zip › Supplementary Data 10U xyz.xyz]

181Title 10U Energy: -1017.93044175 eV   1.C         1.391847    2.172278   -7.436349   2.C         2.252640    0.901357   -7.370098   3.C         0.147345    2.054233   -6.543285   4.C         2.623012    0.549260   -5.920575   5.C         0.530234    1.698928   -5.099700   6.C         1.372777    0.409955   -5.029323   7.C         2.356895   -4.294634   -4.226738   8.C         1.676992   -2.918766   -4.141668   9.C         2.530557   -1.908222   -3.352681  10.C         2.706819   -4.843938   -2.835052  11.C         4.499024    0.705851   -2.823498  12.C        -0.883444   -0.722486   -3.029358  13.C        -2.092523    0.166598   -2.790040  14.C        -2.526312   -5.404786   -2.568414  15.C         2.705840    2.440246   -2.325396  16.C         3.042380    0.936817   -2.368635  17.C        -1.022344   -5.217721   -2.326380  18.C         5.159995    2.986737   -1.950050  19.C         3.518598   -3.834996   -2.009891  20.C         5.487513    1.488480   -1.947006  21.C         2.830297   -2.465673   -1.947739  22.C         3.719517    3.225839   -1.482709  23.C        -3.268061   -5.694293   -1.255365  24.C        -0.763498   -4.134564   -1.273875  25.C        -2.944699    1.626348   -1.041714  26.C         1.796519    6.590442   -0.486517  27.C        -3.162880   -0.808325   -0.805116  28.C        -1.816438    2.633097   -0.861914  29.C         0.309080    6.917548   -0.291443  30.C        -3.005483   -4.600266   -0.208223  31.C        -0.497319    5.645429    0.010654  32.C        -1.500019   -4.390603    0.049470  33.C         2.363609    5.835511    0.723568  34.C        -2.789402   -1.058126    0.651722  35.C         1.540942    4.579861    1.042276  36.C         0.045281    4.889230    1.241145  37.C         1.334668   -4.665726    1.556323  38.C         0.796189   -3.229418    1.731550  39.C         2.832829   -4.762579    1.878763  40.C        -2.750152    3.847827    2.017488  41.C        -2.906557    5.145552    2.836159  42.C        -4.381832    5.557516    2.966520  43.C        -3.600043    2.726492    2.645812  44.C        -5.078150    3.122582    2.759787  45.C        -2.218531   -3.395398    2.825336  46.C        -0.092059    2.463977    3.110065  47.C         1.148874   -2.688225    3.133145  48.C         3.138939   -4.254352    3.292930  49.C        -1.995470   -4.770844    3.484907  50.C        -5.237335    4.426387    3.557588  51.C         2.640404   -2.814918    3.463361  52.C        -0.608154    1.071460    3.524348  53.C        -2.260982   -2.278553    3.883670  54.C        -3.042546   -5.054213    4.574187  55.C        -0.016598    3.403954    4.331791  56.C         0.240154    0.485777    4.658893  57.C        -3.313462   -2.558472    4.964435  58.C        -3.086125   -3.929442    5.620338  59.C         0.844603    2.806725    5.452837  60.C         0.319142    1.426002    5.867004  61.H         1.101623    2.388273   -8.478211  62.H         3.161756    1.025541   -7.982842  63.H         1.686571    0.058840   -7.808225  64.H         1.996403    3.030979   -7.091900  65.H        -0.511766    1.261976   -6.943842  66.H        -0.431937    2.992831   -6.571193  67.H         3.220003   -0.376762   -5.897577  68.H         3.270696    1.345971   -5.514490  69.H         0.759891   -0.398606   -5.479637  70.H         1.467849   -2.544859   -5.157370  71.H         1.714513   -5.011910   -4.766440  72.H         3.284485   -4.191520   -4.819541  73.H         1.104822    2.533140   -4.664044  74.H        -0.371755    1.601702   -4.479181  75.H         0.700003   -3.029102   -3.641488  76.H         3.496841   -1.803415   -3.887105  77.H         4.628981    1.012429   -3.877729  78.H        -0.729302   -0.781048   -4.120991  79.H         2.691444    2.856395   -3.349536  80.H         3.255145   -5.796590   -2.927527  81.H        -2.704319   -6.211183   -3.299235  82.H         5.290284    3.388012   -2.974044  83.H        -0.511633   -4.965688   -3.269566  84.H        -2.992334   -0.206916   -3.321831  85.H        -1.862691    1.166586   -3.180951  86.H        -2.933381   -4.478543   -3.014535  87.H         1.772218   -5.074238   -2.298162  88.H         4.742360   -0.366592   -2.778890  89.H         4.517467   -3.709221   -2.466596  90.H        -1.132734   -1.753472   -2.722864  91.H         6.522859    1.317137   -2.289148  92.H         1.692821    2.587786   -1.913277  93.H        -0.588163   -6.172267   -1.977271  94.H         3.481710    4.300963   -1.508511  95.H         5.863884    3.536216   -1.302612  96.H        -3.662818    1.925552   -1.833609  97.H        -4.350744   -5.798359   -1.441847  98.H        -1.090861   -3.161344   -1.671646  99.H         1.877592   -2.557501   -1.397100 100.H        -0.091940    7.424954   -1.185903 101.H        -1.230852    2.691918   -1.798970 102.H         1.905314    5.956577   -1.385107 103.H         3.430374   -1.760403   -1.353535 104.H         3.682778   -4.226023   -0.993555 105.H         3.002508    0.580896   -1.313805 106.H        -2.917688   -1.712077   -1.378703 107.H         5.412300    1.107836   -0.913268 108.H        -2.920214   -6.663018   -0.852161 109.H         0.315807   -4.024174   -1.100200 110.H         2.374319    7.510461   -0.677267 111.H        -0.437591    4.980505   -0.867665 112.H        -4.247601   -0.606339   -0.929553 113.H         3.626921    2.894868   -0.434846 114.H        -2.262307    3.634198   -0.737590 115.H        -3.455954   -3.657615   -0.566080 116.H        -3.492582    1.557625   -0.093393 117.H        -1.562829    5.897024    0.142904 118.H         0.198075    7.623186    0.552355 119.H         1.642639    3.854473    0.219221 120.H         3.415039    5.560797    0.543165 121.H        -1.109454   -5.338772    0.471617 122.H        -3.524117   -4.852864    0.729883 123.H         1.900800    1.610326    0.559132 124.H         1.160027   -5.024593    0.531178 125.H        -3.470017   -1.833727    1.045026 126.H        -3.193723    4.060038    1.022031 127.H         2.354948    6.505778    1.603016 128.H        -3.016487   -0.148580    1.239259 129.H         1.333622   -2.577253    1.007337 130.H         3.389689   -4.144655    1.152923 131.H        -4.767985    5.813819    1.962753 132.H         3.183181   -5.801184    1.751878 133.H         1.957690    4.074354    1.925974 134.H        -0.052895    5.564185    2.116210 135.H        -5.491210    3.264988    1.744231 136.H        -2.330417    5.963829    2.376484 137.H        -3.492655    1.793154    2.071038 138.H         0.781835   -5.353134    2.223437 139.H        -3.232046   -3.433554    2.373581 140.H        -2.018313   -5.570782    2.726144 141.H         3.211311   -2.152914    2.788594 142.H         0.947147    2.294300    2.759768 143.H        -0.573124    0.367366    2.672369 144.H        -4.476712    6.467489    3.583381 145.H        -5.662837    2.314219    3.230422 146.H         0.857615   -1.631917    3.192251 147.H        -6.297415    4.727279    3.600730 148.H         4.220627   -4.315747    3.498255 149.H        -2.482524    4.998816    3.845291 150.H        -2.447335   -1.307104    3.404239 151.H        -3.213943    2.505570    3.655558 152.H        -0.988264   -4.802495    3.937642 153.H        -4.036447   -5.140856    4.097375 154.H         0.571102   -3.237615    3.897681 155.H         2.636736   -4.909742    4.029808 156.H         0.384845    4.385692    4.034346 157.H        -1.664479    1.119781    3.835289 158.H        -4.917946    4.245167    4.600072 159.H        -1.273273   -2.191863    4.362463 160.H        -4.318030   -2.541002    4.502127 161.H         2.830358   -2.452106    4.486776 162.H         1.254310    0.317486    4.255155 163.H        -2.840477   -6.022505    5.063162 164.H        -1.033778    3.589184    4.724371 165.H         1.879536    2.700508    5.083915 166.H        -0.156400   -0.494136    4.963589 167.H        -3.301675   -1.763261    5.728419 168.H        -2.123496   -3.909067    6.162991 169.H        -3.867191   -4.134184    6.371580 170.H         0.875386    3.487337    6.321090 171.H        -0.687093    1.541719    6.313852 172.H         0.962446    0.986418    6.647636 173.N         0.285743   -0.207774   -2.306304 174.N        -2.357630    0.304992   -1.344511 175.N        -0.970926    2.223149    0.267718 176.N        -1.368171   -1.418003    0.768766 177.Sb        2.688600    0.244415    1.396401 178.Si        1.767688   -0.162482   -3.236966 179.Si       -1.060782   -3.036575    1.339682 180.Si       -0.944016    3.301355    1.638031 181.U         0.066954    0.206358   -0.051241
