# Supplementary material for: Uranium-stibinidiide, -stibinidene, and -stibido multiple bonds and uranium-nitride formation from multimetallic diuranium-distibene-mediated dinitrogen cleavage
Source: Nat Commun. 2025 Aug 4;16:7136. doi: 10.1038/s41467-025-61612-5 (PMC12322283; doi:10.1038/s41467-025-61612-5)
Supplement: Supplementary file 3 — Source Data [file 41467_2025_61612_MOESM3_ESM.zip › Supplementary Data 5U xyz.xyz]

233Title 5U (anion of 5UK) Energy: -1253.91313406 eV   1.C        -2.631677   -0.225776   -7.375799   2.C        -3.219721   -0.072182   -5.954327   3.C        -3.722616    1.371965   -5.758101   4.C        -2.246160   -3.456694   -5.622921   5.C         0.343589    0.717298   -5.200238   6.C        -1.263636   -2.341977   -5.206102   7.C         3.843231   -2.126849   -4.781921   8.C        -0.189573   -2.919289   -4.269357   9.C         4.638280    0.117018   -3.965012  10.C        -0.655817    0.466621   -4.053578  11.C         3.819640   -1.136871   -3.599532  12.C        -4.245410   -1.835149   -3.327145  13.C        -1.110527    1.801656   -3.445013  14.C        -5.517876   -1.027940   -3.096634  15.C         7.059849   -2.436260   -2.675420  16.C         1.760562    4.894896   -2.021505  17.C         5.793652   -3.029390   -2.021573  18.C         2.714431   -4.365996   -2.087011  19.C         2.774290    2.603952   -2.006925  20.C        -5.116118    1.851251   -1.710722  21.C        -6.184990    0.772742   -1.561186  22.C        -4.509401    5.366268   -1.339159  23.C         2.751264   -3.015107   -1.343598  24.C         2.030845    3.645425   -1.155147  25.C        -2.155083    4.491863   -1.203830  26.C        -4.727174   -5.296457   -1.010162  27.C         5.363266    4.852863   -0.770269  28.C         6.181902   -3.782321   -0.732698  29.C        -6.172401   -1.557131   -0.780381  30.C         5.608238    0.212044   -0.757994  31.C        -1.483991   -3.796458   -0.814603  32.C        -3.468459    4.686211   -0.427811  33.C         4.001605    6.757491    0.169291  34.C         4.369029    5.268477    0.330444  35.C         2.632130   -3.269137    0.167498  36.C        -4.947895   -4.829543    0.440547  37.C         6.389624    0.063389    0.544543  38.C        -5.423434   -1.548571    0.548781  39.C        -1.838180   -3.982690    0.670704  40.C         0.648082    5.893819    1.233274  41.C        -1.426751   -5.405756    1.108131  42.C        -5.173041   -6.056818    1.347749  43.C        -5.786388    3.423240    1.187614  44.C         1.542540    4.748967    1.743231  45.C         5.788663    2.022191    1.917212  46.C        -5.892650    4.762816    1.944686  47.C        -6.392443    2.304725    2.049028  48.C        -2.707846    2.645916    1.788713  49.C         4.464653    2.664433    2.320218  50.C        -2.965295    1.329064    2.540455  51.C         5.721587   -0.238299    2.893691  52.C         2.116888    5.104451    3.126969  53.C         4.669724   -1.342918    2.897868  54.C        -3.994729   -3.384073    2.998136  55.C        -2.457277    3.786976    2.799127  56.C         0.760707   -3.246566    3.596071  57.C        -3.683282   -1.989974    3.567046  58.C        -3.329399   -4.457471    3.885247  59.C         1.049371    1.571954    4.040089  60.C         3.003387   -3.856317    4.576340  61.C         0.816206    0.077109    4.311748  62.C         1.845087   -2.837019    4.603950  63.C        -0.099416   -0.105503    5.533622  64.C         3.536372   -0.614597    5.933672  65.C         3.795185    0.888884    6.183570  66.C         3.086094   -1.282081    7.252758  67.H        -3.353541    0.122096   -8.135142  68.H       -2.379031   -1.268200   -7.613781  69.H        -1.715231    0.372955   -7.502129  70.H        -4.525947    1.614107   -6.477138  71.H       -3.036411   -3.100103   -6.300902  72.H       -1.712866   -4.273872   -6.139136  73.H       -0.752153   -2.001274   -6.126885  74.H       -4.108451   -0.729037   -5.911505  75.H        -2.913060    2.100212   -5.923121  76.H        -0.116313    1.292617   -6.021012  77.H         3.556121   -1.616710   -5.718056  78.H         0.734745   -0.216972   -5.627787  79.H         4.849552   -2.549147   -4.939894  80.H        -4.114714    1.545777   -4.746945  81.H         4.256375    0.568469   -4.896973  82.H         1.207651    1.300642   -4.842283  83.H       -2.736980   -3.901087   -4.743278  84.H         3.148104   -2.964744   -4.646348  85.H         0.291859   -3.805270   -4.720022  86.H       -4.285096   -2.238156   -4.352550  87.H         5.701139   -0.119986   -4.136603  88.H        -1.577444    2.458404   -4.193345  89.H         0.601884   -2.191300   -4.044010  90.H       -5.488182   -0.147793   -3.749585  91.H         6.845456   -1.922617   -3.622329  92.H       -6.430231   -1.608430   -3.347514  93.H         2.773876   -0.800343   -3.467396  94.H         7.793235   -3.235049   -2.892019  95.H         4.587881    0.883512   -3.183466  96.H       -0.624646   -3.233287   -3.310286  97.H         2.737424   -4.254276   -3.179207  98.H       -0.115209   -0.067876   -3.246205  99.H         1.152433    4.626121   -2.899530 100.H        2.254594    2.443769   -2.964256 101.H        5.411919   -3.792352   -2.728808 102.H       -0.257637    2.340160   -3.003362 103.H      -4.258910   -2.720743   -2.665087 104.H        2.698775    5.332631   -2.400313 105.H       -1.841487    1.683262   -2.627093 106.H       -4.683295    1.785672   -2.728547 107.H       -7.010398    0.896399   -2.294721 108.H        7.566296   -1.716299   -2.013171 109.H        3.802974    2.918144   -2.239953 110.H       -4.695362    4.770872   -2.248146 111.H       -2.321005    3.864982   -2.091866 112.H        3.562350   -5.010827   -1.803570 113.H         4.932663    4.996826   -1.774581 114.H        1.791726   -4.916527   -1.836452 115.H         1.224022    5.685202   -1.479390 116.H       -4.146701    6.352876   -1.676419 117.H       -5.622935    2.834281   -1.678306 118.H      -4.579094   -4.456203   -1.703478 119.H        6.262167   -0.085202   -1.594272 120.H      -5.591339   -5.881596   -1.374882 121.H        1.846749   -2.445075   -1.633880 122.H        2.829188    1.606640   -1.540367 123.H       -1.748457    5.456391   -1.550610 124.H      -2.041295   -4.488806   -1.462844 125.H      -6.064611   -2.554032   -1.228996 126.H      -3.841993   -5.945743   -1.091485 127.H       7.009833   -4.488201   -0.924453 128.H       6.285144    5.462466   -0.725585 129.H        3.424547    6.942648   -0.750655 130.H        1.056590    3.198332   -0.885149 131.H         5.403113    1.288540   -0.917831 132.H      -0.409699   -3.976424   -0.977056 133.H      -5.479640    5.525723   -0.843546 134.H      -1.671985   -2.776204   -1.182238 135.H      -7.255366   -1.357745   -0.653851 136.H       5.659514    3.796252   -0.694037 137.H      -6.614851    0.858436   -0.557064 138.H      -1.379813    3.993723   -0.604214 139.H         4.914324    7.378805    0.109533 140.H       5.346688   -4.360869   -0.317742 141.H         6.523996   -3.091186    0.055299 142.H         0.091644    5.614104    0.327611 143.H       -0.361378   -5.577002    0.881647 144.H       -3.266099    5.380352    0.408553 145.H       -6.430988    3.519739    0.284103 146.H         7.374063    0.574296    0.502814 147.H        -5.901893   -4.264299    0.453526 148.H         1.789267   -3.948016    0.377382 149.H         3.535723   -3.724992    0.598051 150.H         3.405716    7.134287    1.013637 151.H        -5.975612   -6.698795    0.939998 152.H         6.573327   -1.005995    0.712709 153.H         1.219988    6.807341    1.006977 154.H         2.410560   -2.355948    0.746995 155.H         4.916006    5.195024    1.294759 156.H         6.127941    2.488368    0.985094 157.H        -5.497179   -0.537071    0.987584 158.H        -1.999290   -6.175858    0.567395 159.H        -5.960611   -2.204716    1.256486 160.H        -4.268664   -6.682725    1.421873 161.H        -5.510345    5.616530    1.364075 162.H        -1.221831   -3.258941    1.235421 163.H        -0.099962    6.162389    2.000230 164.H        -1.776320    2.493688    1.216684 165.H        -6.317391    1.313200    1.574933 166.H         0.886904    3.867680    1.881883 167.H        -1.564060   -5.579158    2.183571 168.H        -6.945460    4.986263    2.203440 169.H        -5.467038   -5.775952    2.369183 170.H        -7.463947    2.492683    2.256791 171.H        -3.125951    0.469533    1.869735 172.H         4.822863   -1.981410    2.011559 173.H         4.665288    3.714251    2.600330 174.H         6.577892    2.177442    2.682826 175.H        -2.235213    4.742277    2.306179 176.H         1.108063   -3.126819    2.560138 177.H        -5.329610    4.732681    2.889129 178.H         2.823971    5.949693    3.074084 179.H         6.751161   -0.639050    2.978105 180.H        -5.885754    2.233105    3.024175 181.H        -5.090579   -3.533533    3.083082 182.H         4.108828    2.179936    3.249920 183.H        -4.267021   -1.203219    3.074593 184.H         1.650643    1.757351    3.134921 185.H        -3.848153    1.392651    3.197343 186.H         2.642391    4.257287    3.590104 187.H        -2.100251    1.060361    3.162848 188.H         3.467113   -3.899422    3.579391 189.H        -3.324325    3.942881    3.459787 190.H        -2.622020   -1.729323    3.438868 191.H        -3.522426   -5.483565    3.543200 192.H         1.308712    5.403236    3.818518 193.H         0.472449   -4.304443    3.730371 194.H         0.277652   -0.317687    3.420169 195.H         5.542942    0.409071    3.766204 196.H        -1.599385    3.543858    3.444530 197.H         4.856346   -1.995737    3.764374 198.H        -0.151139   -2.639190    3.698945 199.H        -2.238096   -4.316273    3.921815 200.H         0.088439    2.089032    3.878999 201.H         2.636008   -4.873555    4.803866 202.H        -3.904602   -1.948424    4.648541 203.H        -3.697317   -4.380026    4.924448 204.H         1.555479    2.070147    4.877468 205.H         3.795314   -3.623523    5.303949 206.H         4.103337    1.417580    5.272132 207.H         1.394579   -2.882471    5.616413 208.H        -1.058948    0.421743    5.388031 209.H         4.507701   -1.065223    5.672951 210.H        -0.337104   -1.163578    5.731114 211.H         0.358187    0.306872    6.447677 212.H         2.890339    1.389383    6.560994 213.H         4.584726    1.031562    6.939620 214.H         3.017284   -2.374370    7.166150 215.H         2.099282   -0.914453    7.580223 216.H         3.800151   -1.058195    8.064467 217.N        -3.050812   -1.004225   -3.065702 218.N        -5.561396   -0.561110   -1.691733 219.N         4.353227   -0.565687   -0.687274 220.N        -4.076121    1.690594   -0.677172 221.N        -4.010368   -1.942943    0.345790 222.N         3.475617    2.518296    1.236129 223.N         5.575614    0.578471    1.666896 224.N         3.309994   -0.748344    2.898052 225.Sb        0.009163    0.000766    0.052083 226.Si       -2.077250   -0.727797   -4.514070 227.Si        4.224087   -1.882264   -1.854435 228.Si        2.875847    4.030598    0.533763 229.Si       -4.020703    3.049536    0.450809 230.Si       -3.651546   -3.511963    1.082898 231.Si        2.398054   -0.998175    4.388336 232.U        -2.896705   -0.310541   -0.859659 233.U         2.899230    0.305341    0.893083
