# Supplementary material for: Uranium-stibinidiide, -stibinidene, and -stibido multiple bonds and uranium-nitride formation from multimetallic diuranium-distibene-mediated dinitrogen cleavage
Source: Nat Commun. 2025 Aug 4;16:7136. doi: 10.1038/s41467-025-61612-5 (PMC12322283; doi:10.1038/s41467-025-61612-5)
Supplement: Supplementary file 3 — Source Data [file 41467_2025_61612_MOESM3_ESM.zip › Supplementary Data 6U xyz.xyz]

234Title 6U Energy: -1275.03358097 eV   1.C         5.611738   -1.451237   -5.583557   2.C         2.139788   -2.477231   -4.652635   3.C         4.324324    1.917414   -4.671408   4.C         3.347285    0.730552   -4.604411   5.C        -4.992564    2.284697   -4.501888   6.C        -4.400876    4.736766   -4.377644   7.C        -1.249967    2.876261   -4.421016   8.C        -3.373259   -3.265375   -4.419486   9.C         1.922819    1.221452   -4.317127  10.C         5.517256   -1.402891   -4.046565  11.C         5.865705   -2.779653   -3.456763  12.C        -4.605650    3.443293   -3.566700  13.C        -1.990546    0.611728   -3.566405  14.C         2.425916   -1.869488   -3.267530  15.C        -2.634881   -3.760136   -3.163448  16.C        -1.730176    2.076978   -3.195438  17.C        -1.189866   -3.243201   -3.157604  18.C        -5.180633   -5.651692   -2.199981  19.C         2.572114   -2.971278   -2.211414  20.C        -4.943909   -1.114601   -2.325058  21.C        -2.441781    4.654331   -1.686539  22.C        -5.216812   -4.270569   -1.519066  23.C         5.392275    0.840165   -1.663121  24.C        -6.182778   -0.836000   -1.485726  25.C         2.790211    3.266039   -1.312685  26.C        -6.178447    1.572750   -0.946130  27.C        -3.447523    5.449732   -0.835592  28.C         6.428056    0.059323   -0.871552  29.C        -1.147314    4.404028   -0.898973  30.C        -5.079365    2.513930   -0.477400  31.C        -5.861925   -4.385595   -0.127255  32.C        -2.162134   -5.335872   -0.098197  33.C        -2.361064   -3.808818   -0.111339  34.C         5.888792    4.376936    0.106218  35.C         2.176202    5.274584    0.085709  36.C         2.400272    3.750406    0.087885  37.C         5.090641   -2.519593    0.453325  38.C        -6.429039   -0.086966    0.852393  39.C         3.436317   -5.442108    0.780013  40.C         6.196868   -1.592927    0.930131  41.C         1.137471   -4.391822    0.877112  42.C         6.240867    0.812065    1.467971  43.C        -2.739417   -3.302986    1.284668  44.C         5.245001    4.256658    1.498529  45.C        -5.405832   -0.867920    1.660398  46.C         2.442315   -4.647003    1.645043  47.C         5.189076    5.638202    2.177635  48.C         5.020809    1.108156    2.327454  49.C        -2.532952    2.874089    2.195099  50.C         2.655325    3.710941    3.135871  51.C         1.217177    3.175076    3.108490  52.C        -5.849737    2.748917    3.447282  53.C         1.743722   -2.070385    3.159211  54.C         4.609623   -3.461116    3.529199  55.C        -2.418394    1.799348    3.282663  56.C         2.028395   -0.621434    3.574638  57.C        -5.520104    1.375916    4.056059  58.C         4.397528   -4.765511    4.320801  59.C         3.387057    3.222583    4.397875  60.C         4.999326   -2.319067    4.482953  61.C        -1.964034   -1.297293    4.345628  62.C         1.230556   -2.888652    4.358653  63.C        -4.378017   -1.957810    4.682405  64.C        -3.382842   -0.785656    4.624705  65.C        -2.140796    2.436071    4.655981  66.C        -5.621429    1.440522    5.591781  67.H         6.594362   -1.842905   -5.897851  68.H         5.490156   -0.459176   -6.040199  69.H         4.844628   -2.111497   -6.016813  70.H         3.342533    0.248645   -5.599798  71.H         4.034244    2.618507   -5.471783  72.H         1.960034   -1.706504   -5.415846  73.H        -4.206606    2.094929   -5.248008  74.H        -2.878567   -3.629112   -5.335330  75.H         2.983342   -3.095162   -4.996979  76.H        -3.542130    4.656934   -5.061233  77.H        -5.290502    4.952151   -4.994132  78.H        -5.919217    2.513194   -5.054998  79.H        -2.024168    2.920048   -5.202207  80.H         5.359372    1.601235   -4.871769  81.H         1.670730    2.074764   -4.967166  82.H        -0.360304    2.411841   -4.867384  83.H         1.251773   -3.125078   -4.620348  84.H        -4.420392   -3.602919   -4.455847  85.H         1.174702    0.436543   -4.493540  86.H        -3.371307   -2.166053   -4.465393  87.H        -0.986670    3.911633   -4.158749  88.H        -2.827081    0.491915   -4.267572  89.H         6.886713   -3.084971   -3.742062  90.H        -5.155920    1.343085   -3.958698  91.H         6.300047   -0.697221   -3.707806  92.H         5.179341   -3.556110   -3.827021  93.H        -0.703777   -3.441012   -4.126057  94.H        -4.228341    5.608368   -3.730586  95.H         4.322186    2.483525   -3.728788  96.H        -1.097747    0.167302   -4.033024  97.H        -4.838348   -5.591775   -3.242683  98.H         1.801915    1.551917   -3.275531  99.H        -2.601892   -4.864965   -3.206123 100.H        -5.471192    3.626907   -2.902138 101.H         1.551709   -1.256502   -2.975554 102.H        -1.147757   -2.158072   -2.980158 103.H        -5.196961   -1.821140   -3.129013 104.H         5.810938   -2.794799   -2.359458 105.H        -4.644333   -0.185251   -2.838102 106.H         5.812745    1.122464   -2.639466 107.H        -2.200683   -0.033467   -2.695388 108.H        -6.186855   -6.105061   -2.207131 109.H         3.407144   -3.648420   -2.435870 110.H        -2.191397    5.268423   -2.571588 111.H        -0.912955    2.065490   -2.449295 112.H         1.659084   -3.581203   -2.158732 113.H        -0.582993   -3.723712   -2.378431 114.H        -5.885037   -3.638501   -2.135041 115.H        -7.047803   -0.528423   -2.101252 116.H        -6.187057    1.573456   -2.042655 117.H        -4.513018   -6.349226   -1.671502 118.H         2.034169    3.571368   -2.052283 119.H         3.755259    3.674764   -1.641189 120.H        -4.395885    5.631618   -1.364095 121.H         6.606957   -0.894533   -1.382578 122.H         2.747642   -2.579947   -1.197344 123.H        -0.339997    4.027342   -1.541821 124.H         5.187617    1.791201   -1.146434 125.H         2.862896    2.169098   -1.397756 126.H        -6.455556   -1.756496   -0.956455 127.H        -1.806871   -5.715462   -1.067340 128.H        -3.034456    6.432503   -0.553803 129.H         7.393519    0.593929   -0.803321 130.H        -5.334378    3.544443   -0.767432 131.H        -7.181209    1.880718   -0.595490 132.H         0.107619   -1.406215   -0.717464 133.H        -6.884933   -4.792706   -0.197725 134.H         1.435761    5.558907   -0.676573 135.H         5.307589    5.045266   -0.546963 136.H        -0.792073    5.330939   -0.420303 137.H         5.059452   -2.514642   -0.646190 138.H         5.965800    3.408455   -0.407533 139.H         3.108105    5.813263   -0.144747 140.H        -1.392142   -3.341638   -0.366313 141.H        -3.680463    4.916004    0.097667 142.H        -1.299536    3.659948   -0.104808 143.H         3.664221   -4.905412   -0.152456 144.H         6.906974    4.795696    0.177082 145.H        -5.927104   -3.417554    0.388497 146.H        -3.101424   -5.858460    0.139130 147.H        -5.287674   -5.061056    0.524665 148.H         1.272225   -3.636532    0.090722 149.H         7.196260   -1.911333    0.578132 150.H         1.436278    3.267199    0.333443 151.H        -5.044612    2.512960    0.623178 152.H         0.780446   -5.313956    0.390925 153.H        -1.423680   -5.627555    0.663421 154.H        -7.391902   -0.621845    0.757970 155.H         3.015128   -6.421191    0.497404 156.H         5.329682   -3.554036    0.742843 157.H         6.516303    1.729023    0.932884 158.H         1.811542    5.642021    1.056010 159.H        -6.618656    0.864705    1.363758 160.H         4.388324   -5.632039    1.299113 161.H        -5.193086   -1.820907    1.149827 162.H        -2.792209   -2.203693    1.351319 163.H         4.515129    6.327232    1.646130 164.H         0.336843   -4.029241    1.536219 165.H        -3.710711   -3.690496    1.621283 166.H        -2.662640    2.461136    1.181115 167.H         6.189753    6.103489    2.187927 168.H         6.205812   -1.600191    2.026726 169.H         7.111593    0.498076    2.072726 170.H         5.922656    3.635561    2.115090 171.H        -1.987550   -3.612540    2.026888 172.H        -5.794047    2.745748    2.349875 173.H         2.202354   -5.264552    2.530594 174.H        -5.842870   -1.147179    2.630372 175.H         0.611493    3.663209    2.332959 176.H        -1.621409    3.488055    2.158650 177.H        -3.379396    3.552624    2.366579 178.H         4.732202    0.193950    2.870959 179.H         0.935605   -2.024622    2.403837 180.H         5.476414   -3.638940    2.865201 181.H         4.843841    5.575131    3.219259 182.H         5.289405    1.839863    3.104604 183.H         2.308605    0.040291    2.736894 184.H         2.607340    4.814953    3.180525 185.H         1.189763    2.092422    2.911641 186.H        -1.551361    1.164427    3.016867 187.H        -6.866560    3.072829    3.726780 188.H         4.230623   -5.628475    3.661024 189.H        -1.846362   -1.634489    3.306249 190.H        -5.152562    3.521199    3.805939 191.H        -6.308087    0.674437    3.719944 192.H        -4.377429   -2.521521    3.738062 193.H         5.162541   -1.368621    3.955482 194.H         0.719214    3.348332    4.075660 195.H         4.427425    3.578598    4.447144 196.H         1.127670   -0.160468    4.008292 197.H         0.948415   -3.911226    4.067917 198.H         2.834116   -0.546695    4.316902 199.H         3.405384    2.123399    4.438432 200.H         5.282381   -4.988885    4.941240 201.H         5.926928   -2.558495    5.029707 202.H        -1.206110   -0.521292    4.519387 203.H        -5.409580   -1.626580    4.876385 204.H        -1.237813    3.063431    4.622997 205.H         3.533502   -4.694992    4.998812 206.H         0.346076   -2.415791    4.806038 207.H         2.875774    3.573564    5.309516 208.H        -1.724826   -2.150780    5.000478 209.H         1.995093   -2.968275    5.146533 210.H         4.215414   -2.142214    5.234342 211.H        -2.975598    3.080203    4.972787 212.H        -4.104375   -2.665456    5.482718 213.H        -1.989975    1.679645    5.439825 214.H        -3.377577   -0.305155    5.620904 215.H        -6.601928    1.843525    5.898123 216.H        -4.850341    2.098054    6.022023 217.H        -5.510582    0.451659    6.058162 218.N         4.154484    0.040429   -1.780991 219.N        -3.849434   -1.598196   -1.456106 220.N        -3.792122    2.052732   -1.030112 221.N        -5.854363    0.203978   -0.481690 222.N         5.883532   -0.222995    0.474230 223.N         3.806078   -2.042255    0.998783 224.N         3.913181    1.560030    1.461544 225.N        -4.170809   -0.068533    1.795082 226.Sb       -0.005594    0.147573    0.065791 227.Si        3.873157   -0.640136   -3.381121 228.Si       -3.159731    3.017580   -2.361562 229.Si       -3.531879   -3.326865   -1.534068 230.Si        3.575299    3.288642    1.516310 231.Si        3.168434   -3.016319    2.324860 232.Si       -3.883734    0.592612    3.399945 233.U        -3.141349    0.091884   -0.177554 234.U         3.115138   -0.088524    0.182366
