# Supplementary material for: Uranium-stibinidiide, -stibinidene, and -stibido multiple bonds and uranium-nitride formation from multimetallic diuranium-distibene-mediated dinitrogen cleavage
Source: Nat Commun. 2025 Aug 4;16:7136. doi: 10.1038/s41467-025-61612-5 (PMC12322283; doi:10.1038/s41467-025-61612-5)
Supplement: Supplementary file 3 — Source Data [file 41467_2025_61612_MOESM3_ESM.zip › Supplementary Data 7U xyz.xyz]

234Title 7U Energy: -1256.13720287 eV   1.C         5.756799    1.603049   -5.594712   2.C         4.417308   -1.743520   -4.894508   3.C        -4.478467   -4.666565   -4.584106   4.C         2.237991    2.678658   -4.847792   5.C        -5.038885   -2.211948   -4.651350   6.C         3.432797   -0.564377   -4.761487   7.C        -1.244253   -2.926609   -4.666566   8.C         2.008071   -1.095884   -4.547995   9.C        -3.472847    3.551988   -4.328392  10.C         5.631962    1.496180   -4.057815  11.C        -4.640563   -3.386435   -3.734713  12.C         6.046732    2.840183   -3.425515  13.C        -1.941254   -0.659997   -3.788288  14.C         2.524765    2.095207   -3.449468  15.C        -1.698310   -2.135102   -3.422189  16.C        -2.769871    4.054137   -3.051647  17.C        -1.265136    3.754625   -3.139675  18.C        -5.386706    5.756095   -2.060754  19.C         2.696194    3.229418   -2.423851  20.C        -4.870393    1.234547   -2.218137  21.C        -2.517580   -4.735312   -1.923691  22.C         5.291265   -0.786516   -1.724891  23.C        -5.356603    4.364761   -1.389618  24.C        -6.121496    0.881567   -1.421966  25.C         2.836250   -3.592978   -1.440873  26.C        -3.556862   -5.575700   -1.154097  27.C        -6.100855   -1.547500   -0.974342  28.C        -1.216114   -4.649412   -1.110882  29.C         6.334558   -0.049082   -0.894863  30.C        -4.970869   -2.492359   -0.589459  31.C        -6.021534    4.455489   -0.000970  32.C         2.372300   -5.663002   -0.066592  33.C         6.021463   -4.455277    0.000903  34.C         2.491070   -4.125428   -0.041318  35.C        -2.371798    5.662554    0.065453  36.C        -2.491385    4.125067    0.040914  37.C         4.971248    2.492377    0.589237  38.C        -6.335012    0.048432    0.894615  39.C         6.101045    1.547140    0.973834  40.C         3.557271    5.575835    1.154813  41.C         1.216443    4.649565    1.112325  42.C         5.356263   -4.364894    1.389404  43.C         6.120852   -0.881863    1.421940  44.C        -2.836807    3.593391    1.440679  45.C         5.386273   -5.756352    2.060310  46.C        -5.292043    0.786312    1.724612  47.C         2.518281    4.735284    1.924578  48.C         4.869478   -1.234625    2.217794  49.C        -2.696501   -3.229163    2.423365  50.C         2.769579   -4.054751    3.051324  51.C         1.264752   -3.755721    3.139657  52.C        -6.046895   -2.840103    3.425566  53.C         1.699197    2.134847    3.422401  54.C        -2.525086   -2.095003    3.449083  55.C         4.641610    3.385934    3.734757  56.C         1.942122    0.659876    3.788811  57.C        -5.632246   -1.496087    4.057781  58.C         3.472634   -3.552779    4.328095  59.C         4.479580    4.666105    4.584172  60.C         5.039887    2.211517    4.651387  61.C        -2.007941    1.095608    4.547595  62.C         1.244563    2.926446    4.666650  63.C        -4.417031    1.743969    4.893888  64.C        -3.432797    0.564555    4.761192  65.C        -2.238124   -2.678584    4.847288  66.C        -5.756766   -1.602835    5.594657  67.H         5.620021    0.636262   -6.098697  68.H         6.756681    1.980005   -5.872441  69.H         5.019854    2.303495   -6.017179  70.H         4.136346   -2.388476   -5.744625  71.H         3.442227   -0.022319   -5.725863  72.H        -4.275463   -2.030512   -5.423642  73.H        -5.386020   -4.843953   -5.187115  74.H        -3.634843   -4.588464   -5.286707  75.H         2.046477    1.899062   -5.599738  76.H        -2.024598   -2.941467   -5.442999  77.H        -5.983641   -2.428874   -5.178918  78.H        -3.022234    4.010011   -5.225626  79.H         3.077934    3.291220   -5.210855  80.H         1.761032   -1.857864   -5.306232  81.H         5.455188   -1.418689   -5.061431  82.H        -0.349413   -2.467815   -5.113977  83.H         1.350640    3.331391   -4.817296  84.H         1.253263   -0.300194   -4.621557  85.H        -0.993989   -3.970914   -4.428112  86.H        -4.547203    3.789206   -4.347401  87.H        -3.365886    2.462134   -4.437662  88.H        -2.783650   -0.534937   -4.482558  89.H        -4.313880   -5.563112   -3.969954  90.H        -5.176394   -1.268543   -4.102807  91.H         6.380891    0.745787   -3.739967  92.H         4.403701   -2.377120   -3.995435  93.H         7.094014    3.082093   -3.674791  94.H         5.427972    3.666784   -3.807249  95.H        -1.050055   -0.229426   -4.271776  96.H        -0.842110    4.164830   -4.072905  97.H         1.895635   -1.567556   -3.560073  98.H        -5.499290   -3.572833   -3.061218  99.H        -5.067884    5.724080   -3.111941 100.H        -2.880380    5.155372   -3.032290 101.H         1.626526    1.523893   -3.140168 102.H        -1.069607    2.671555   -3.135919 103.H        -5.127712    1.960953   -3.003769 104.H        -2.288186   -5.279008   -2.860335 105.H         5.726495   -1.044375   -2.703233 106.H        -4.533606    0.334197   -2.762411 107.H        -2.137813   -0.015474   -2.913254 108.H         5.957282    2.848348   -2.329851 109.H        -0.862726   -2.151080   -2.695976 110.H         3.553711    3.873676   -2.662798 111.H        -6.410711    6.168393   -2.041571 112.H         1.799921    3.870146   -2.406806 113.H        -0.701279    4.192162   -2.303517 114.H        -6.958487    0.576538   -2.076830 115.H        -5.999243    3.712539   -2.011720 116.H        -6.157513   -1.500563   -2.069300 117.H         2.095723   -3.937742   -2.180662 118.H        -4.738797    6.478100   -1.540255 119.H        -4.512229   -5.675068   -1.690689 120.H         3.822285   -3.936215   -1.784763 121.H        -0.395708   -4.194360   -1.684337 122.H         6.537877    0.919852   -1.367833 123.H         5.067265   -1.750635   -1.241363 124.H         2.832679    2.857570   -1.397605 125.H        -3.174891   -6.595148   -0.975589 126.H         2.837301   -2.492477   -1.489373 127.H        -5.228988   -3.512191   -0.916137 128.H        -6.443055    1.768220   -0.862733 129.H         7.290939   -0.602684   -0.839402 130.H        -2.058724    6.075515   -0.905052 131.H        -7.082610   -1.895051   -0.600098 132.H         1.625354   -5.978037   -0.813353 133.H         5.484986   -5.157756   -0.655432 134.H        -0.883671   -5.653436   -0.795383 135.H         3.325194   -6.141086   -0.340434 136.H        -7.058660    4.823586   -0.086959 137.H         6.057292   -3.487739   -0.521161 138.H         4.904710    2.544918   -0.509187 139.H        -3.769357   -5.137477   -0.167513 140.H         7.058511   -4.823466    0.086925 141.H        -1.352652   -4.045548   -0.201556 142.H        -1.491025    3.723720   -0.206873 143.H        -6.057161    3.488193    0.521408 144.H         3.769402    5.137867    0.168016 145.H        -3.324476    6.141303    0.338958 146.H         1.490549   -3.724713    0.206648 147.H        -5.485019    5.158299    0.654991 148.H        -4.904383   -2.544775    0.508918 149.H         1.352384    4.045441    0.203097 150.H         7.082796    1.894319    0.599404 151.H        -7.291653    0.601579    0.839210 152.H         0.884132    5.653649    0.796717 153.H         2.059570   -6.076584    0.903764 154.H        -1.624766    5.977569    0.812156 155.H         6.442414   -1.768639    0.862860 156.H         3.175305    6.595328    0.976749 157.H         5.229635    3.512045    0.915984 158.H        -6.537899   -0.920637    1.367531 159.H         4.738476   -6.478278    1.539571 160.H        -5.068377    1.750424    1.241052 161.H         4.512869    5.674989    1.691078 162.H        -2.839107    2.492869    1.489577 163.H        -2.832852   -2.857227    1.397163 164.H         6.410322   -6.168648    2.041160 165.H         0.396179    4.194935    1.686270 166.H        -3.822397    3.937813    1.784684 167.H         5.998728   -3.712749    2.011817 168.H         6.957665   -0.576841    2.076936 169.H         6.157899    1.500268    2.068871 170.H        -2.095726    3.937557    2.180202 171.H        -5.957848   -2.848234    2.329813 172.H         0.700938   -4.193131    2.303398 173.H        -1.800272   -3.869911    2.406397 174.H        -3.554096   -3.873377    2.662187 175.H        -5.727407    1.044055    2.702954 176.H         2.289296    5.278811    2.861458 177.H         5.067368   -5.724549    3.111436 178.H         4.532529   -0.333973    2.761600 179.H         5.126509   -1.960645    3.003878 180.H         0.863713    2.150851    2.696087 181.H         5.500373    3.572340    3.061258 182.H         2.880360   -5.155974    3.031709 183.H         2.138430    0.015108    2.913759 184.H         1.068878   -2.672734    3.136363 185.H        -1.626942   -1.523634    3.139741 186.H        -7.094051   -3.082267    3.675154 187.H        -5.427837   -3.666595    3.807024 188.H        -6.381337   -0.745753    3.739992 189.H        -1.895133    1.566474    3.559341 190.H         4.317335    5.562943    3.970008 191.H         0.841986   -4.166464    4.072773 192.H        -4.403266    2.377331    3.994642 193.H         5.177632    1.268019    4.102732 194.H         4.547073   -3.789671    4.346838 195.H         1.050488    0.229371    4.272196 196.H         3.365386   -2.462950    4.437672 197.H         2.784092    0.534713    4.483040 198.H         0.995331    3.970902    4.428305 199.H        -1.350752   -3.331227    4.816610 200.H         5.386340    4.842108    5.188912 201.H        -1.253291    0.299760    4.621951 202.H         5.984523    2.428448    5.179021 203.H        -5.454924    1.419497    5.060983 204.H         3.022331   -4.011131    5.225292 205.H         3.634648    4.588960    5.285296 206.H        -3.077995   -3.291270    5.210387 207.H         0.349721    2.468126    5.113525 208.H        -1.760889    1.858074    5.305315 209.H         4.276402    2.029844    5.423511 210.H         2.025010    2.940803    5.443602 211.H        -2.046620   -1.899060    5.599316 212.H        -4.135798    2.389137    5.743845 213.H        -6.756388   -1.980536    5.872595 214.H        -3.442435    0.022675    5.725641 215.H        -5.019277   -2.302667    6.017160 216.H        -5.620706   -0.635871    6.098498 217.N         4.057197    0.041183   -1.839958 218.N        -3.806093    1.728356   -1.303052 219.N        -3.692188   -2.002635   -1.170765 220.N        -5.784916   -0.194899   -0.459859 221.N         5.784565    0.194566    0.459602 222.N         3.692529    2.002748    1.170475 223.N         3.805447   -1.728676    1.302638 224.N        -4.057645   -0.040995    1.839884 225.Sb        0.001553    1.201070   -0.606423 226.Sb       -0.001583   -1.200987    0.606873 227.Si        3.917586    0.777585   -3.462670 228.Si       -3.150192   -3.015686   -2.532448 229.Si       -3.604062    3.503550   -1.395359 230.Si        3.603613   -3.503912    1.395048 231.Si        3.150971    3.015611    2.532741 232.Si       -3.917975   -0.777340    3.462343 233.U        -3.002575   -0.070889   -0.175748 234.U         3.002543    0.071101    0.175711
