# Supplementary material for: Uranium-stibinidiide, -stibinidene, and -stibido multiple bonds and uranium-nitride formation from multimetallic diuranium-distibene-mediated dinitrogen cleavage
Source: Nat Commun. 2025 Aug 4;16:7136. doi: 10.1038/s41467-025-61612-5 (PMC12322283; doi:10.1038/s41467-025-61612-5)
Supplement: Supplementary file 3 — Source Data [file 41467_2025_61612_MOESM3_ESM.zip › Supplementary Data 9UK xyz.xyz]

224Title 9UK Energy: -1252.42606590 eV     1.C         2.554270    5.198562   -5.914927   2.C         1.342856    4.254671   -5.916895   3.C        -3.744691   -2.117062   -5.341186   4.C        -0.235088   -6.552235   -4.543509   5.C         2.606978    6.043875   -4.633532   6.C        -4.598003   -1.061070   -4.627477   7.C        -2.373614   -2.251106   -4.665456   8.C         1.319253    3.386089   -4.651212   9.C         0.967695   -7.151075   -3.799717  10.C        -0.964064   -5.496235   -3.697345  11.C         1.922178   -6.055388   -3.302888  12.C         2.258170   -1.512113   -3.724177  13.C         0.765762   -1.219896   -3.745778  14.C         2.565549    5.166857   -3.371265  15.C         1.330134    4.241025   -3.367541  16.C        -0.002571   -4.384169   -3.223689  17.C        -4.724954   -1.354138   -3.125540  18.C        -2.507239   -2.624060   -3.174650  19.C         3.252896    0.658146   -3.204594  20.C        -3.774081    3.297371   -2.675866  21.C        -1.550250    2.063900   -2.713894  22.C        -3.071123    1.940079   -2.574308  23.C         1.172245   -5.019244   -2.455828  24.C        -3.363962   -1.574500   -2.446740  25.C         3.091023    1.696107   -2.105557  26.C        -1.692634    4.430970   -1.814688  27.C         4.028325   -1.343601   -2.032345  28.C        -3.207870    4.274338   -1.640415  29.C        -0.969014    3.072516   -1.703212  30.C         3.525494   -2.174901   -0.863339  31.C        -2.225393   -4.434101   -0.353090  32.C        -1.221014   -3.268859   -0.483807  33.C        -6.211491    1.327928   -0.409298  34.C         1.431425    4.389707   -0.280298  35.C         7.372958    0.740024    0.147314  36.C         2.930296    4.222963    0.052889  37.C        -6.261917    2.716971    0.182320  38.C        -6.246238   -0.971576    0.128657  39.C        -0.035421   -3.488687    0.478615  40.C         6.384721   -0.348144    0.590814  41.C        -2.717084   -4.591129    1.090422  42.C         3.376957    5.137719    1.200861  43.C         0.591048    4.143804    0.987918  44.C         7.767120    1.644663    1.323731  45.C        -6.400292   -1.956872    1.261032  46.C        -4.935208    4.213593    1.496411  47.C         5.123515    0.252473    1.247507  48.C        -1.540309   -4.801742    2.052963  49.C        -0.509567   -3.672686    1.926271  50.C         6.530562    2.229441    2.020555  51.C         1.026666    5.044246    2.152683  52.C        -3.599064    4.270576    2.198152  53.C         3.574347   -3.894840    2.056433  54.C         4.529715   -2.686455    2.140526  55.C         4.282759   -5.203820    2.428908  56.C         2.525916    4.910226    2.457061  57.C         5.546420    1.129588    2.445733  58.C         2.032536    1.038126    2.582331  59.C        -5.276470   -2.769518    3.197818  60.C         2.674204   -0.308674    2.956275  61.C         5.177649   -2.599909    3.538309  62.C         4.932069   -5.111926    3.818376  63.C         5.884113   -3.910736    3.917207  64.C         1.605618   -1.231088    3.567613  65.C        -3.992243   -2.607750    3.973201  66.C        -2.410335    3.414088    4.052883  67.C         1.407345    1.713320    3.808979  68.C         0.403378    0.788438    4.507101  69.C         0.990202   -0.592645    4.819115  70.C        -2.564004    2.572818    5.296947  71.C        -2.732842   -1.096755    5.264807  72.C        -2.752846    0.264180    5.918412  73.H         2.538792    5.848995   -6.804060  74.H         1.354708    3.620770   -6.818970  75.H         0.413865    4.851271   -5.960091  76.H        -3.629385   -1.856142   -6.406092  77.H         3.477018    4.593869   -5.983466  78.H         0.118043   -6.075296   -5.475800  79.H        -4.259761   -3.094126   -5.305922  80.H        -0.932622   -7.351601   -4.843416  81.H        -5.596914   -0.990613   -5.088423  82.H        -1.759583   -2.992634   -5.201534  83.H         1.502064   -7.866230   -4.445579  84.H         1.739193    6.727763   -4.616289  85.H         3.508831    6.677713   -4.632211  86.H        -4.120684   -0.074755   -4.764158  87.H         0.452110    2.710396   -4.667562  88.H        -1.845928   -1.285322   -4.747110  89.H         2.212980    2.740693   -4.659375  90.H        -1.797414   -5.071523   -4.276979  91.H         2.739697   -1.349119   -4.708729  92.H         2.377878   -5.546727   -4.171938  93.H         0.302644   -1.814057   -4.550175  94.H         0.425116   -3.935041   -4.144660  95.H         2.555316    0.906655   -4.012592  96.H        -3.630968    3.714211   -3.689571  97.H         0.600976   -0.163149   -4.021115  98.H        -1.305495    2.377240   -3.742951  99.H         0.601695   -7.725284   -2.929385 100.H         0.454627    4.919958   -3.438236 101.H         4.277834    0.651297   -3.625130 102.H        -3.468168    1.250589   -3.332329 103.H         2.395804   -2.565600   -3.449668 104.H        -3.042636   -3.594439   -3.130433 105.H        -5.343383   -2.260762   -2.989580 106.H        -1.417266   -5.988076   -2.817940 107.H         2.749154   -6.501013   -2.725974 108.H         3.489779    4.561763   -3.332660 109.H         2.575086    5.805237   -2.474644 110.H        -1.496070    4.879178   -2.804892 111.H        -5.264914   -0.528511   -2.634641 112.H        -4.862194    3.174041   -2.542637 113.H        -1.093450    1.068982   -2.573979 114.H         4.601974   -1.951061   -2.760108 115.H        -2.803568   -0.629585   -2.402351 116.H         3.421471    2.670832   -2.498907 117.H         1.866349   -4.243746   -2.099793 118.H        -3.710030    5.254369   -1.707014 119.H         0.784005   -5.517369   -1.550785 120.H        -3.275352    1.489641   -1.585927 121.H         4.697229   -0.567014   -1.641810 122.H        -3.511979   -1.860959   -1.393610 123.H        -1.295788    5.140387   -1.070918 124.H        -6.995032    1.227752   -1.183704 125.H         3.789926    1.464850   -1.280736 126.H        -3.084697   -4.276818   -1.022588 127.H         2.850688   -2.957784   -1.245982 128.H         3.552566    4.411067   -0.834379 129.H        -3.403911    3.861453   -0.638543 130.H        -1.746949   -5.376031   -0.676984 131.H        -5.226014    1.170255   -0.879398 132.H        -1.192521    2.678763   -0.691627 133.H        -6.243402    3.454300   -0.641371 134.H         6.897076    1.353177   -0.639396 135.H         1.271305    5.441523   -0.594780 136.H         8.271149    0.286359   -0.303133 137.H        -7.001855   -1.186057   -0.649469 138.H         4.383388   -2.716343   -0.430249 139.H        -5.242414   -1.080006   -0.316473 140.H         6.123987   -0.986824   -0.266369 141.H        -1.734741   -2.346733   -0.139040 142.H         0.545573   -4.373174    0.172122 143.H         3.107342    3.171782    0.337184 144.H        -7.193523    2.861688    0.761478 145.H         0.696157   -2.659702    0.454944 146.H        -4.932394    4.924219    0.650737 147.H         8.435420    2.451516    0.982572 148.H         3.282062    6.190237    0.877875 149.H        -0.478191    4.302854    0.781152 150.H        -3.432422   -5.427327    1.168634 151.H         4.660891    0.935315    0.504515 152.H        -6.444661   -2.979703    0.842089 153.H         6.883590   -1.012137    1.319581 154.H         4.442308    4.971175    1.430656 155.H        -3.258403   -3.669241    1.363318 156.H         0.669113    3.083074    1.278271 157.H         3.132722   -3.968851    1.052759 158.H         6.011189    2.915203    1.327216 159.H         5.356919   -2.882656    1.428314 160.H         5.064870   -5.413789    1.676706 161.H         8.341358    1.047835    2.055413 162.H        -2.825074    3.812779    1.554963 163.H        -1.057375   -5.768566    1.820968 164.H         0.803642    6.095611    1.893718 165.H        -7.337448   -1.765902    1.817182 166.H        -5.750383    4.493002    2.189489 167.H        -0.964426   -2.721571    2.253709 168.H        -3.325943    5.324500    2.394720 169.H         3.574914   -6.048090    2.392883 170.H         1.204256    0.876488    1.854246 171.H        -1.409447   -0.749087    2.039419 172.H         0.350802   -3.861774    2.585969 173.H         2.752721    1.714831    2.100459 174.H         6.833227    2.832167    2.892387 175.H         2.725103    3.896894    2.841591 176.H        -1.896697   -4.875826    3.094363 177.H         6.735291   -4.066614    3.230040 178.H        -5.327162   -3.800268    2.800858 179.H         0.435565    4.812214    3.054948 180.H         2.819740    5.611828    3.254750 181.H         2.725956   -3.741063    2.743052 182.H         6.021477    0.495156    3.215587 183.H         4.669799    1.589159    2.923431 184.H         0.812581   -1.408218    2.823392 185.H         5.893540   -1.762793    3.578714 186.H         5.466707   -6.045564    4.056365 187.H        -3.136590   -2.643021    3.277203 188.H        -1.706099    2.920163    3.356075 189.H        -6.150479   -2.602916    3.855105 190.H         3.418641   -0.105765    3.754911 191.H         0.898163    2.642044    3.511364 192.H         2.032146   -2.211278    3.823575 193.H         4.399745   -2.378191    4.291459 194.H         4.136624   -5.001729    4.577849 195.H        -2.015425    4.410431    4.326995 196.H         6.306544   -3.840391    4.933105 197.H        -0.468962    0.671351    3.842573 198.H        -3.892040   -3.440195    4.695079 199.H        -1.944686   -1.118789    4.491270 200.H         2.214874    1.996463    4.509613 201.H         0.212290   -1.251617    5.241478 202.H         0.047834    1.265176    5.435681 203.H        -3.360326    2.977788    5.949045 204.H         1.775599   -0.500132    5.592277 205.H        -1.610699    2.585130    5.854891 206.H        -2.508436   -1.867631    6.026130 207.H        -3.589217    0.342987    6.637819 208.H        -1.802912    0.402185    6.465719 209.K        -4.332630    0.764351    2.614808 210.N         2.888270   -0.673388   -2.684115 211.N         0.205027   -1.499492   -2.413760 212.N         1.689682    1.717073   -1.642747 213.N         2.820140   -1.323062    0.113122 214.O        -6.417047    0.361165    0.629869 215.O        -5.116882    2.880356    1.015272 216.O        -5.269870   -1.828178    2.124029 217.O        -3.695130    3.543727    3.428047 218.O        -4.007774   -1.352515    4.661138 219.O        -2.876492    1.241371    4.888673 220.Sb       -1.541083    0.523614    0.847981 221.Si       -0.830130   -2.909237   -2.309042 222.Si        0.916411    3.281844   -1.751716 223.Si        3.738682   -1.040136    1.579231 224.U         0.989115   -0.223283   -0.721249
