# Supplementary material for: Uranium-stibinidiide, -stibinidene, and -stibido multiple bonds and uranium-nitride formation from multimetallic diuranium-distibene-mediated dinitrogen cleavage
Source: Nat Commun. 2025 Aug 4;16:7136. doi: 10.1038/s41467-025-61612-5 (PMC12322283; doi:10.1038/s41467-025-61612-5)
Supplement: Supplementary file 3 — Source Data [file 41467_2025_61612_MOESM3_ESM.zip › Supplementary Data 9UNa xyz.xyz]

2179UNa Energy: -1213.25085476 eV   1.C         2.226111    0.824479   -5.778721   2.C         2.704320   -1.542174   -5.641725   3.C        -1.554766   -0.351630   -5.285728   4.C        -2.554459    0.650671   -4.698574   5.C         2.229977    2.037772   -4.872074   6.C         3.061182   -2.570150   -4.587998   7.C        -0.682718   -0.946069   -4.176153   8.C        -3.418287    0.000174   -3.611572   9.C         3.642423    3.036649   -3.213159  10.C        -5.188452   -5.016717   -2.891618  11.C        -1.520345   -1.582726   -3.063129  12.C        -3.300117    4.197137   -2.999783  13.C        -1.770446    4.151870   -3.102922  14.C        -4.776989   -3.541730   -2.761210  15.C         4.609815   -2.919373   -2.800648  16.C        -3.988526   -5.956123   -2.699162  17.C         5.069300    2.995543   -2.690246  18.C        -2.570757   -0.614442   -2.474344  19.C         5.828394   -2.300563   -2.149267  20.C        -3.743863    4.987257   -1.760628  21.C         6.565618   -0.142270   -1.370400  22.C        -1.138539    3.584796   -1.825638  23.C        -4.056193   -3.266521   -1.425375  24.C        -3.280069   -5.693086   -1.361675  25.C        -2.866006   -4.222027   -1.233940  26.C        -6.616002    1.660020   -0.993131  27.C         6.044463    1.276590   -1.260750  28.C        -5.274833    0.952404   -0.766522  29.C        -5.338544   -0.544050   -1.113158  30.C         0.783252   -4.040590   -1.108108  31.C         1.701014   -5.253695   -0.908904  32.C        -3.111358    4.410981   -0.485760  33.C        -1.571238    4.355970   -0.562207  34.C        -7.726806    0.983745   -0.175327  35.C        -7.804078   -0.522631   -0.466411  36.C         2.923179   -4.903331   -0.049883  37.C        -6.445269   -1.215709   -0.273652  38.C         3.118321    4.895324    0.014487  39.C         0.367486   -3.415029    0.228634  40.C         1.661907    4.923384    0.492612  41.C         1.085840    3.501341    0.676074  42.C         3.992327    4.063392    0.960467  43.C         3.414828    2.659151    1.169646  44.C         1.599248   -3.016480    1.060447  45.C         2.509927   -4.247791    1.272402  46.C        -3.301156   -1.987461    1.554625  47.C         1.959248    2.689333    1.652202  48.C        -2.854786    2.028799    1.802537  49.C         3.830610   -0.818989    2.143553  50.C        -1.148391    6.209461    2.343255  51.C        -3.701390   -0.943253    2.589893  52.C        -1.197227    4.679993    2.533106  53.C         5.221961   -0.344263    2.591059  54.C        -2.913809    1.314484    3.140609  55.C         3.064938   -1.537507    3.271895  56.C        -1.640497    6.939736    3.603543  57.C         5.176529    0.489656    3.880525  58.C        -0.383225    4.270049    3.777493  59.C        -0.481800   -4.210132    3.602784  60.C        -0.284632   -0.246744    3.848932  61.C        -1.744100   -0.628147    4.062000  62.C         0.681469   -3.324568    4.091103  63.C         3.015769   -0.648003    4.531065  64.C        -0.855818    6.510037    4.852886  65.C         4.419828   -0.240302    4.997930  66.C        -1.135380   -4.982759    4.755835  67.C         1.725598   -4.185043    4.831522  68.C        -0.879264    4.985420    5.040670  69.C        -0.096992   -5.824905    5.512021  70.C         1.085436   -4.968276    5.988746  71.H         2.991371    0.926216   -6.570207  72.H         3.514587   -1.451438   -6.389020  73.H         1.778438   -1.855366   -6.157758  74.H         1.233109    0.716279   -6.249603  75.H        -0.928106    0.137650   -6.051128  76.H        -2.109244   -1.156419   -5.803735  77.H        -3.195416    1.075268   -5.488414  78.H         1.899536    2.930268   -5.433408  79.H         3.142408   -3.573019   -5.043633  80.H        -1.998175    1.491209   -4.249867  81.H         0.012097   -1.696999   -4.586383  82.H        -4.035805   -0.792869   -4.071640  83.H        -5.663138   -5.198197   -3.869943  84.H         1.545560    1.859425   -4.020493  85.H        -3.738968    4.629740   -3.913180  86.H         2.271683   -2.586959   -3.815299  87.H         3.373568    4.083816   -3.447585  88.H        -1.463048    3.554895   -3.976596  89.H        -0.056280   -0.158066   -3.724376  90.H        -4.107959   -3.280368   -3.601067  91.H         5.754753    3.494917   -3.393777  92.H        -4.122285    0.743504   -3.212524  93.H        -2.035157   -2.472935   -3.463022  94.H        -3.269804   -5.792694   -3.523021  95.H        -1.388889    5.175927   -3.268991  96.H         6.649360   -2.220926   -2.885232  97.H        -5.664455   -2.895736   -2.860920  98.H         4.811540   -3.980150   -3.034452  99.H        -3.683926    3.164031   -2.924494 100.H        -4.309860   -7.007951   -2.766203 101.H         7.319717   -0.200656   -2.176145 102.H        -5.950074   -5.242448   -2.123238 103.H         2.940181    2.642930   -2.453234 104.H        -0.844174   -1.932433   -2.269033 105.H        -1.998948    0.226289   -2.012174 106.H        -6.870421    1.619120   -2.067330 107.H         3.751762   -2.867215   -2.107790 108.H         5.102662    3.523518   -1.726015 109.H        -3.437552    6.042844   -1.873163 110.H        -2.101788   -4.003593   -1.995054 111.H        -4.843053    4.982033   -1.677060 112.H         1.408645   -1.244970   -1.901578 113.H        -5.658398   -0.619390   -2.172967 114.H        -0.043099    3.576293   -1.924228 115.H         6.164019   -2.938682   -1.312471 116.H        -4.469518    1.461478   -1.317561 117.H        -1.421502    2.521942   -1.716750 118.H         2.017032   -5.656460   -1.885699 119.H         1.307553   -3.260309   -1.687264 120.H        -2.399659   -6.348784   -1.259431 121.H        -0.103760   -4.326893   -1.690222 122.H         7.037063   -0.438987   -0.416631 123.H        -6.537832    2.726158   -0.725184 124.H         6.880499    1.942856   -0.983335 125.H        -8.128640   -0.671744   -1.511860 126.H        -4.782751   -3.512731   -0.623141 127.H         3.140768    4.448115   -0.993544 128.H        -3.964187   -5.949384   -0.532418 129.H        -8.699836    1.461841   -0.371425 130.H        -1.202546    5.397475   -0.655750 131.H         3.566885   -4.196933   -0.599392 132.H        -2.388122   -4.037172   -0.261718 133.H        -3.495402    3.387973   -0.345656 134.H         5.260209    1.337190   -0.486729 135.H         1.131645   -6.058805   -0.409802 136.H        -5.018321    1.047642    0.300408 137.H         1.168156    2.991363   -0.305202 138.H        -6.537630   -2.281795   -0.530891 139.H         3.514612    5.919923   -0.081148 140.H         3.530813   -5.803838    0.139611 141.H         1.048600    5.502592   -0.215400 142.H         3.428102    2.095527    0.218861 143.H        -3.436145    4.991706    0.392922 144.H         5.026842    3.998942    0.579588 145.H        -0.287017   -2.551566    0.006420 146.H        -8.569437   -0.994580    0.171517 147.H        -7.515083    1.131194    0.899679 148.H         2.153549   -2.277533    0.448484 149.H        -6.171193   -1.176338    0.796518 150.H        -0.270168   -4.119036    0.786392 151.H        -4.187069   -2.603540    1.319418 152.H        -3.540702    1.521803    1.109080 153.H         4.041762    2.098198    1.875863 154.H         3.937969   -1.473363    1.265424 155.H         1.616169    5.460576    1.455985 156.H         1.586265    1.654070    1.734745 157.H        -1.755826    6.509729    1.474399 158.H         4.058034    4.580856    1.934863 159.H        -2.570449   -2.682160    2.004549 160.H         5.697227    0.238306    1.783225 161.H         3.408221   -3.963973    1.842129 162.H         3.232232    0.033786    1.787767 163.H        -3.262527    3.043637    1.918700 164.H         1.977760   -4.994219    1.887531 165.H        -0.114690    6.527956    2.123921 166.H        -4.466052   -0.297955    2.137616 167.H         1.912702    3.122268    2.663984 168.H         5.864059   -1.228010    2.761351 169.H        -2.255672    4.445511    2.765512 170.H        -0.102836   -4.932728    2.859350 171.H        -4.138910   -1.400588    3.498244 172.H        -1.570725    8.031666    3.468665 173.H        -1.232789   -3.600699    3.076461 174.H        -3.913300    1.388623    3.610227 175.H         4.662933    1.445731    3.678231 176.H         3.634993   -2.450319    3.541778 177.H        -2.710632    6.707149    3.752646 178.H         0.677267    4.529430    3.617014 179.H        -2.186131    1.779060    3.817408 180.H        -0.191764    0.852979    3.825003 181.H        -0.412079    3.176904    3.913172 182.H         6.197573    0.746366    4.207277 183.H        -1.818271   -1.723038    4.054673 184.H         2.438188    0.263938    4.301042 185.H         2.191194   -4.896640    4.127257 186.H        -1.949034   -5.624972    4.380589 187.H         0.193996    6.838722    4.747067 188.H         0.298864   -0.574747    4.724089 189.H        -2.141584   -0.262826    5.030124 190.H         0.255349   -2.646744    4.859092 191.H         0.281149   -6.614026    4.837099 192.H        -1.913610    4.659863    5.254421 193.H         2.483582   -1.160477    5.349260 194.H         4.978259   -1.148053    5.289959 195.H         2.541199   -3.554016    5.217582 196.H        -1.253791    7.014333    5.748074 197.H        -1.596813   -4.260611    5.453902 198.H         4.359252    0.397973    5.894893 199.H        -0.269587    4.698549    5.913297 200.H        -0.566456   -6.340121    6.365533 201.H         1.837192   -5.601865    6.487260 202.H         0.726434   -4.248521    6.746958 203.N        -2.725997   -1.320739    0.377301 204.N        -1.476163    2.012648    1.273927 205.N         0.187013   -0.827234    2.579213 206.N        -2.534845   -0.100045    2.932358 207.Na        3.810987    0.057239   -3.045401 208.O         2.513174   -0.309326   -4.957475 209.O         3.573669    2.212090   -4.387011 210.O         4.312512   -2.181973   -3.995150 211.O         5.511575    1.630292   -2.545002 212.O         5.461002   -1.000689   -1.676964 213.Sb        1.439263    0.360338   -1.219992 214.Si       -3.637627   -1.427887   -1.109766 215.Si       -0.781676    3.598150    1.006162 216.Si        1.336325   -2.139701    2.728641 217.U      -0.852480  -0.066096    0.732616
